# Mechanistic Study on Steric Activity Interplay of Olefin/Polar Monomers for Industrially Selective Late Transition Metal Catalytic Reactions

**DOI:** 10.3390/molecules28207148

**Published:** 2023-10-18

**Authors:** Andleeb Mehmood, Ayyaz Mahmood, Najla AlMasoud, Arzoo Hassan, Taghrid S. Alomar, Zeinhom M. El-Bahy, Nadeem Raza, Xiaoqing Tian, Naeem Ullah

**Affiliations:** 1 College of Physics and Optoelectronic Engineering, Shenzhen University, Shenzhen 518000, China; 2 Department of Chemistry, College of Science, Princess Nourah bint Abdulrahman University, P.O. Box 84428, Riyadh 11671, Saudi Arabia; 3 Department of Chemistry, Faculty of Science, Al-Azhar University, Nasr City, Cairo 11884, Egypt; 4 Chemistry Department, Imam Mohammad Ibn Saud Islamic University (IMSIU), Riyadh 11623, Saudi Arabia

**Keywords:** computational study, metal catalysts, electronics and steric, copolymerization mechanisms, plastic materials

## Abstract

A significant issue in developing metal-catalyzed plastic polymer materials is obtaining distinctive catalytic characteristics to compete with current plastics in industrial commodities. We performed first-principle DFT calculations on the key insertion steps for industrially important monomers, vinyl fluoride (VF) and 3,3,3-trifluoropropene (TFP), to explain how the ligand substitution patterns affect the complex’s polymerization behaviors. Our results indicate that the favorable 2,1-insertion of TFP is caused by less deformation in the catalyst moiety of the complexes in contrast to the 1,2-insertion mode. In contrast to the VF monomer, the additional interaction between the fluorine atoms of 3,3,3-trifluoropropene and the carbons of the catalyst ligands also contributed to favor the 2,1-insertion. It was found that the regioselectivity of the monomer was predominated by the progressive alteration of the catalytic geometry caused by small dihedral angles that were developed after the ligand–monomer interaction. Based on the distribution of the 1,2- and 2,1-insertion products, the activity and selectivity were influenced by the steric environment surrounding the palladium center; thus, an increased steric bulk visibly improved the selectivity of the bulkier polar monomer (TFP) during the copolymerization mechanism. In contrast, better activity was maintained through a sterically less hindered Pd metal center; the calculated moderate energy barriers showed that a catalyst with less steric hindrance might provide an opportunity for a wide range of prospective industrial applications.

## 1. Introduction

The introduction of halogen groups into polyolefin enhances the polymers’ essential qualities and generates substantial revenue for the chemical, petrochemical, automotive, metallurgical, and food industries, and other industries [1,2]. Among halogenated polymers, fluorinated ethylene propylene (FEP) can be produced through a range of approaches including the radical copolymerization of ethylene and vinyl halides; however, additional synthetic methods, such as ring-opening polymerization, acyclic diene metathesis polymerization processes, and post-polymerization fluorination or defluorination, can also be used to produce partially fluorinated polyolefins. After polymerization, poly(acrylic acid) or its esters were fluorinated using SF_4_ and HF to produce CF_3_ side groups [3,4,5,6,7]. It is well known that neutral (phosphine–sulfonate) Pd-based catalysts produce linear copolymers with limited CH_3_ branching (<10 CH_3_/10^3^ C) and have outstanding functional group selectivity in copolymerization of ethylene (C_2_H_4_) processes [8]. Jordan et al. utilized a palladium phosphine–sulfonate system to create the first coordination–insertion copolymerization of vinyl fluoride (VF) and C_2_H_4_, and a very low VF content (0.09–0.55 mol%) was obtained with reduced molecular weights (Mn) of up to 2 × 10^4^ g/mol compared to ethylene homopolymerization under the same conditions [9,10,11,12,13,14]. Further, an upsurge in the vinyl fluoride (VF) incorporation level (3.6 mol%) was reported by utilizing the phosphine-bis(arenesulfonate) ligand PPh(2-SO_3_Li-4-MePh)(2-SO_3_-4-Me-Ph)([Li-OPO]) unit as a result of the ligand modification procedure.

In a different investigation, high fluorine contents of up to 15 wt. % (8.9 mol% TFP and 4.6 mol% of F) were attained by copolymerizing ethylene and 3,3,3-trifluoropropene (TFP) using the palladium phosphine–sulfonate system [15]. However, 4-fluorostyrene and fluorinated acrylates were used as fluorinated comonomers for C_2_H_4_ insertion copolymerization, but TFP appears to be the ideal comonomer for C_2_H_4_ coordination–insertion copolymerization to access significantly fluorinated, well-defined, and linear polyolefins, which cannot be produced using conventional methods like the radical polymerization process, etc. [16,17]. However, sterically demanding phosphine–sulfonate palladium complexes were the subjects of experimental and theoretical studies on the insertion of methacrylate (MA), and it was discovered that steric interactions between the ligand and the monomer could completely invert the regioselectivity, resulting in 1,2-insertion [18,19,20,21,22]. A comprehensive study of the copolymerization of ethylene and TFP revealed that while 1,2-insertion is considerably more prevalent, TFP may incorporate in a Pd-Me bond via both 1,2 and 2,1 modes. It also demonstrates that when following the β-H transfer to the palladium center, chain walking becomes less challenging compared to direct chain release in the case of 2,1-insertion of TFP. It is intriguing to compute the reported complexes that behave differently in halogenated polymerization because the marginal activation energies for 2,1- and 1,2-insertion of TFP suggest that 2,1-insertion may have a considerably lower chain-propagation-to-chain-termination ratio. These observations sparked our interest in the mechanistic investigation of C_2_H_4_ and 3,3,3-trifluoropropene (TFP) copolymerization in order to provide some mechanistic reasons for how ligands in phosphine–sulfonate Pd complexes can influence the fluorine incorporation content in the copolymer chain. We conducted some theoretical investigations [23,24,25,26,27] on halogenated and non-halogenated polymers, but the reported catalytic activities have remained a challenge, and the yield has not been sufficient for commercialization; thus, we demonstrated some newly designed complexes to deal with these limitations.

**Scheme 1:**
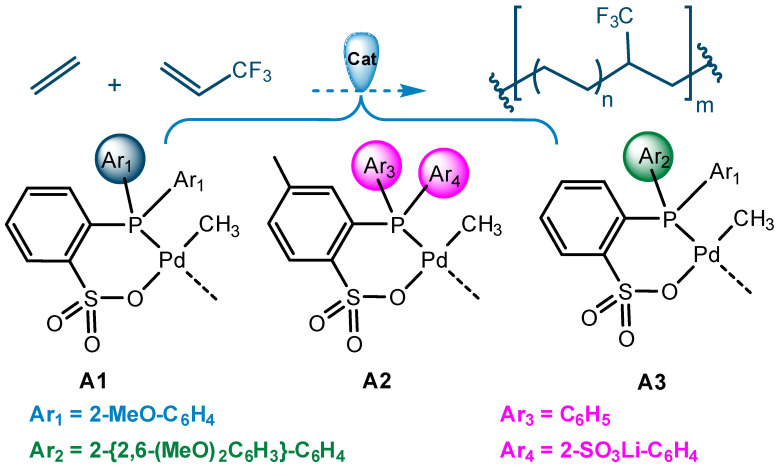
Presentation of ethylene-3,3,3-Trifluropropene copolymerization using phosphine–sulfonate PdMe bearing a range of different ligands as **Ar_1_** (blue), **Ar_2_** (green), **Ar_3_** and, **Ar_4_** (Pink).

The catalysts that were the subjects of this investigation are depicted in Scheme 1 and Figure S1. The selected catalyst system, **A1** (PdII complex [PO]Pd(Me)(py), where [PO] = 2-PAr2-4-Me-benzenesulfonate; Ar = 2-OMe-Ph), experimentally proved that the copolymerization of ethylene with vinyl fluoride in toluene at 80 °C results in fluorinated polyethylene. Low incorporation rates of vinyl fluoride (0.1–0.5 mol%) are noted with the synthesis of a linear copolymer with Mw = 35,000, Mw/Mn = 3.0, and 0.17 mol% vinyl fluoride incorporation at 80 psi vinyl fluoride and 220 psi ethylene pressures. Increasing the amount of vinyl fluoride in the substrate yields an upsurge in the level of vinyl fluoride incorporation (to 0.45 mol%) and decreases in the polymer yield and molecular weight at a total pressure of 300 psi [13]. In the same context, **A1** has also demonstrated a higher fluorine content (4.6 mol%) in E/TFP copolymer formation in comparison to the reported E/VF copolymerization with a low fluorine content (0.34 mol%) [15,28,29]. 

A unique system, **A2** [(phosphinoarenesulfonate)Pd complex as (PO^Bp,OMe^)Pd, where PO^Bp,OMe^ = (2-MeOC_6_H_4_)(2-{2,6-(MeO)_2_C_6_H_3_}C_6_H_4_)(2-SO_3_-5-MeC_6_H_3_)P], is reported to be the most effective catalyst for E/VF copolymerization because it introduces vinyl fluoride (VF) to create (PO^Bp,OMe^)PdCH_2_CHF_2_(lutidine) and multiple ethylene (E) units to develop polyethylene that contains CH_2_F chain ends, ultimately resulting in a polymer backbone chain with more fluorine when compared to **A1** [14], whereas the **A3** (phosphine-bis(arenesulfonate) ligand, PPh(2-SO_3_Li-4-MePh)(2-SO_3_-4-Me-Ph)([Li-OPO]), was the most efficient catalyst studied to copolymerize ethylene and VF and create linear copolymers with a substantially higher amount of VF incorporation (up to 3.6 mol%) than what is typically seen with (PO)PdMe(L) catalysts (<0.5 mol%). Our estimations will emphasize the activities for E/TFP copolymerization due to the high levels of VF integration and the continuous effectiveness of this complex for vinyl fluoride copolymers. Herein, a detailed analysis could also aid in the process of reaching the requisite activity of catalysts in order to create a high-yield polymeric material [14,20,30].

## 2. Results and Discussion

### 2.1. Chain Initiation and Propagation Using ***A1***, ***A2***, and ***A3*** Complexes

Phosphine–sulfonate-based catalysts, **A1**, **A2**, and **A3**, have been selected as computational models for our comparative study, and previous research has shown that the polymerization reaction via phosphine–sulfonate-based complexes begins with viable isomerization from the more stable trans complex to its’ cis-isomer, followed by the more kinetically preferred cis-isomer insertion; as a result, a thorough mechanism of E-TFP copolymerization was investigated using the species A_trans_ (Figure S1) [31,32]. 

For the chain initiation step, the coordination of ethylene and TFP occurs at the trans site on the available position formed by the release of the ligand (DMSO) moiety in the pre-catalyst; the unsymmetrical nature of the palladium phosphine–sulfonate catalyst allows for the monomer insertion on both the *cis* and *trans* sides, but *trans* coordination is more stable with low energy as compared to the *cis* coordination shown on the *trans* site (Figures S1 and S2) [33]. Prior to beginning the computation of complex **A1**, we looked at the experimentally obtained findings that 3,3,3-trifluoropropene (TFP) is substantially integrated into the polymer backbone rather than being mostly located at the chain ends. In a detailed observation, we postulate that functionalities other than CH_2_ groups from the linear backbone, such as CF_3_–, CH_3_–, or olefinic groups, are the primary sources of prominent signals. The strongest peak in the ^19^F NMR spectra is by far the dominating signal, demonstrating that TFP is primarily integrated into the polymer backbone rather than being primarily found at the chain ends [15]. For all of the complexes (**A1**, **A2**, and **A3**) employed in our investigation, the initial ethylene insertion in the Pd-Me bond will occur for this purpose in order to produce a polyethylene chain (as a Pd-C bond). The feasibility of ethylene (E) and polar monomer 3,3,3-trifluoropropene (TFP) insertion will also be compared in the Pd-Me bond to estimate the activity of all complexes in detail. 

Figure S2a–c dictate the computed chain initiation step and indicate two possible routes for the ethylene monomers and TFP insertions (**A1_Coor1_** → **A1_Coor2_** → **A1_TS1_** → **A1_P1_**, and **A1**:**12/21_Coor1_** → **12/21_Coor2_** → **12/21_TS1_** → **12/21_P1_**); in Pd-Me bonds, both of these insertion pathways (for E and TFP) exhibit highly exergonic natures and show nearly identical energy barriers (ΔG_A1_^‡^ = 19.9, 19.1, and 19.0 kcal mol^−1^ for E, 1,2-, and 2,1-TFP, respectively). Through this step, we were able to identify poor regioselectivity utilizing direct TFP insertion into the Pd-Me bond in the chain initiation stage, with a difference of 0.1 kcal mol^−1^. According to theoretical studies on the insertion regiochemistry of different alkenes to cationic (α-Me)diimine)palladium(II), the difference between the activation energies for 2,1- and 1,2-insertion is quite smaller for TFP than that of MA and AN (ΔΔ*E* = 4.8, 6.4, and 5.4 kcal mol^−1^, respectively) [19]. The abovementioned details suggest that the experimental findings, earlier research, and our computed results are all in conformity.

As further indicated in Figure S2d, it is interesting that the regioselectivity of TFP is reversed when compared to the vinyl fluoride insertion. Through a geometric analysis of the involved transition state structures (**12/21_TS1_**), it was disclosed that the additional fluorine (F1 among F2 and F3) in the case of the 3,3,3-trifluoropropene (TFP) monomer establishes some more interactions with the carbons (C4, C5, and C6) of one of the Ar ligands in the **A1** complex, which is not apparent in the case of the VF monomer structure, and these fluorine atoms are inducing a minor favorability for the 2,1-insertion mode for TFP. Meanwhile, in addition to geometric considerations, the electronic and steric factors may also be able to affect regioselectivity. Experimental and theoretical investigations on the insertion of MA in sterically demanding phosphine–sulfonate palladium complexes revealed that steric interactions between the ligand and the monomer could completely invert the regioselectivity, leading to 1,2-insertion [18].

**Figure 1:**
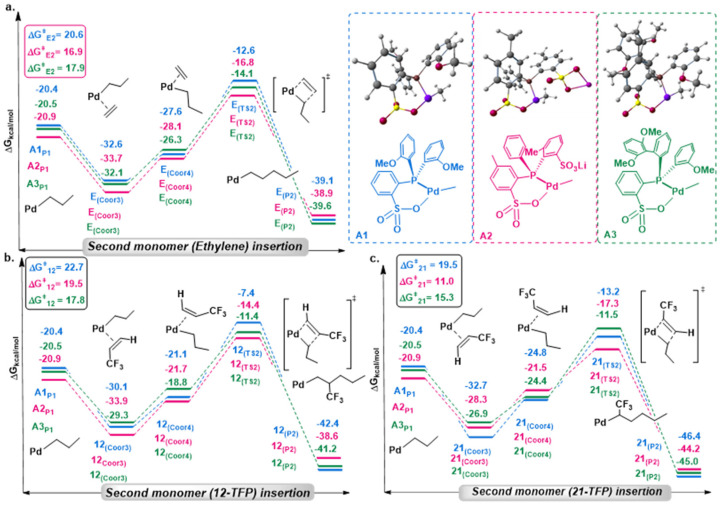
Calculated energy profiles for (**a**) E, (**b**) E/1,2-TFP, and (**c**) E/2,1-TFP copolymerization mediated by **A1** (blue), **A2** (pink), and **A3** (green). Free energies are relative to the energy sum of catalysts and monomers (E/TFP).

In order to achieve copolymer –CH_2_–CHCF_3_–CH_2_– units, further insertions of monomers (E, 1,2-TFP, and 2,1-TFP) based on the product **A1_P1_** are calculated, and the obtained energy barriers (ΔG_E2_^‡^ = 20.6, ΔG_12_^‡^ = 22.7, and ΔG_21_^‡^ = 19.5 kcal mol^−1^ in Figure 1 and Figure S3) demonstrate that 2,1-TFP-insertion is preferred over 1,2-TFP-insertion, which is in contrast to the VF findings, where the 1,2-insertion is slightly favorable during VF insertion in the polymer chain. Therefore, via the 1,2- and 2,1- pathways, thermodynamically stable copolymer products might be obtained, aiding in the synthesis of–CH_2_–CHCF_3_–CH_2_–units [15]. Similar to the aforementioned, the activities of the analogs (**A2** and **A3**) were also evaluated, and the first E-insertion in the Pd-Me bonds was successfully accomplished with moderate total energy barriers (ΔG_A2_^‡^ =20.9 kcal mol^−1^, and ΔG_A3_^‡^ =20.0 kcal mol^−1^, Figure S2) and produced stable precursors, **A2_P1_** and **A3_P1_** (**A2_P1_**—20.9, and **A3_P1_**—20.5 kcal mol^−1^). 

The subsequent TFP insertions into these precursors that occurred with moderate energy berries of 19.5 and 11.0 kcal mol^−1^ (for 1,2-insertions in **A2**) and of 17.8 and 15.3 kcal mol^−1^ (for 2,1-insertions in **A3**) indicate that a 2,1-insertion pathway was most favorable through **AX_P1_** (**X** = **A1**, **A2**, and **A3** complexes). However, if the **12_P2_** and **21_P2_** go through the β-H elimination, the polymeric olefin with an internal double bond may lead to saturated terminating end groups (Figure S3); thus, we did not consider this route for further insertions. The fact that the ^19^F NMR spectra show that TFP is mainly incorporated into the polymer backbone instead of being mostly located at the chain ends, and according to our computed data, the **AX_P1_** → **21_Coor3_** → **21_Coor4_** → **21_TS2_** → **21_P2_** pathway is the most advantageous, with complex **A2** displaying superior selectivity in regard to E/TFP copolymerization.

**Figure 2:**
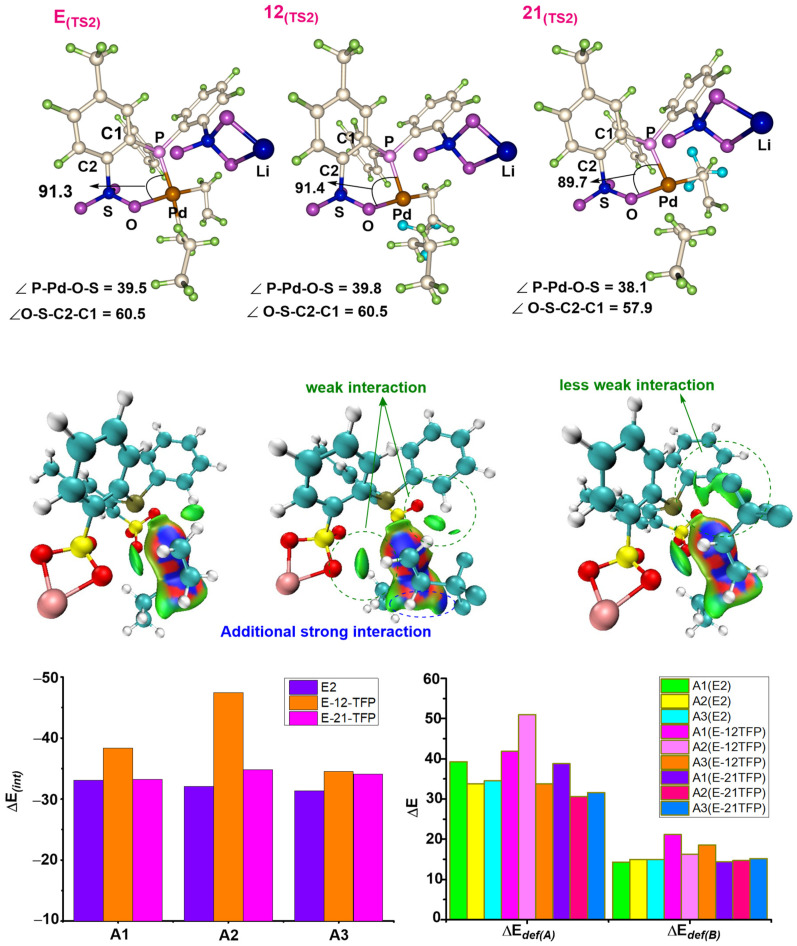
EDA and NCI analysis for key transition state; all energies are calculated in kcal/mol.

**Figure 3:**
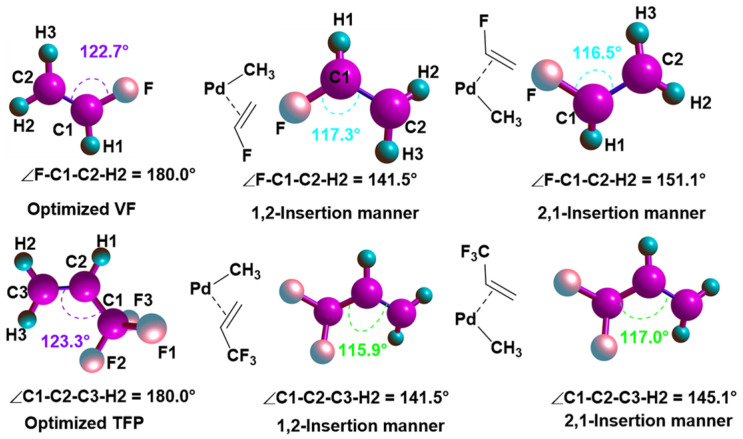
Geometrical parameter analysis of 1,2/2,1-insertion manners for vinyl fluoride (VF) and 3,3,3-Trifluropropene (TFP) in Pd-Me bond as 1st monomer mediated by **A1**.

Based on the above results, to access the origin of selectivity in the prominent **A2** complex, we performed a non-covalent interaction analysis (NCI) via an independent gradient model (IGM) [34,35,36] to investigate the crucial interaction zone between two specific fragments (shown in Figure 2) (all details can be seen in Figure S4 and Table S1). In the preceding geometries, Δ*E*_def(A)_ and Δ*E*_def(B)_ indicate the deformation energies of the catalyst (A) and monomer moiety (B). The interaction energy between the two fragments (A and B) is shown by the *E*_int_. The relation Δ*E* = Δ*E*_int_ + Δ*E*_def(A)_ + Δ*E*_def(B)_ is used to calculate the energy of the Δ*E*. Among all insertions (shown in Figure 1) the stability of **21_(TS2)_** could be ascribed to the least total deformation energy +45.3 (30.6 + 14.7) kcal mol^−1^ in comparison to +67.2 (51 + 16.2) kcal mol^−1^ for the **12_(TS2)_** of **A2_._** A significantly small deformation created a stable transition state (−14.4 vs. −17.3 kcal mol^−1^), which offset the 2,1-TFP-insertion’s less total interaction (Δ*E*_int_) energy (−47.4 vs. −34.8 kcal mol^−1^). The same phenomena were observed during the EDA of **A1** and **A3** (Δ*E*_def(**A1**)_ = +63.0 vs. 53.1 and Δ*E*_def(**A3**)_ = +52.4 vs. 46.6 for 1,2/2,1-insertions); consequently, the TFP 2,1-insertion mode was preferred due to less deformation in the catalysts and monomer moieties than in the vinyl fluoride insertions ^23^ (**A2** insertion modes and geometrical parameters are given in Figure 3 and Figure S5). A thorough geometrical investigation also confirmed that the smaller dihedral (∠P-Pd-O-S = 38.1 vs. 39.8, and ∠O-S-C2-C1 = 57.9 vs. 60.5) and bite angles (∠P-Pd-O = 89.7 vs. 91.4) of **21_(TS2)_** induce the rigidity in the catalyst, which may have an impact on the chelate ring size and favors the overall stability [23,26,37]. Furthermore, the dihedral angles of the monomer (TFP) differed on a substantial scale after insertion in **A1** (dihedral angle is ∠C1-C2-C3-H1 = 141.5 vs. 145.1 for 1,2/2,1-insertion), while 2,1-insertion was discovered to be close to the ideal monomer bonding (∠C1-C2-C3-H1 = 180.0 vs. 141.5 vs. 145.1 for 1,2/2,1-insertion), and an analysis of the monomer moiety (TFP) was determined to be beneficial to execute insertion activity in a specific manner. It might be inferred that 2,1-insertion selectivity happens as a result of a significant deviation from optimal geometrical parameters and steric bulk surrounding the metal center, which significantly affects the chain propagation rates because of its vast catalytic flexibility. Thus, the chain propagation through catalyst **A1** is slower than the catalysts **A2** and **A3**.

**Figure 4:**
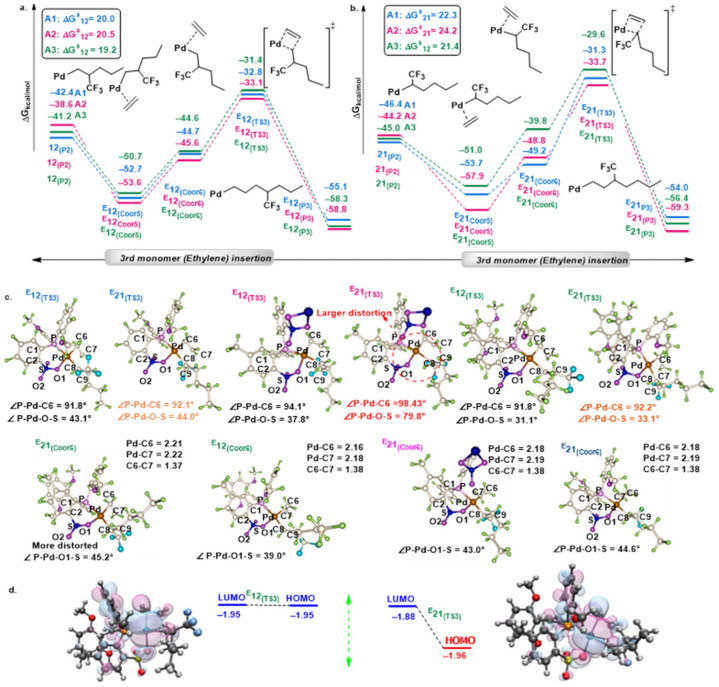
Calculated energy profiles for E-insertion as 3rd monomer for copolymerization mediated by **A1**, **A2**, and **A3** in sections (**a**,**b**). Section (**c**) presents the geometrical and natural bond orbital analysis (NBO), and (**d**) shows orbital analysis of important structures. Free energies are relative to the energy sum of catalysts and monomers (E/TFP). All energies are calculated in kcal mol^−1^.

Regardless, the activity of each complex (**A1**, **A2**, and **A3**) is further assessed via third monomer insertion (as E) into ethylene and the TFP pre-inserted intermediates (**12_(P2)_** and **21_(P2)_**) to investigate the fact that the subsequent insertion of C_2_H_4_ is reportedly prevented by electron-withdrawing substituents in the growing polymer chain’s α-position [20,21]. As indicated in Figure 4a,b, the direct E-insertion in **AX12_(P2)_** overcomes the moderate total energy barriers (20.0, 20.5, and 19.2 kcal mol^−1^) and yields the copolymer –CH_2_–CHCF_3_–CH_2_–unit. In contrast, E-insertion in **AX21_(P2)_** consumes more energy (22.3, 24.2, and 21.4 kcal mol^−1^). 

Meanwhile, the third ethylene coordination in **E_(P2)_** shows normal energy variations with total energy barriers of 20.3, 20.0, and 19.6 kcal mol^−1^ (**A1**, **A2**, and **A3**, respectively) (Figure 4a,b and Figure S6). In this study, a lower negative energy value (−39.8 kcal mol^−1^) was noticed for the **^E^21_(Coor6)_** formation via the 2,1-TFP-insertion pathway in **A3**, which is due to the larger distortion (18.0 vs. 16.4 kcal mol^−1^) in the catalytic moiety (Figure 4c,d and Figure S6). Such poor stability is further justified by larger dihedral angles (45.2 vs. 39.0) and it is anticipated that a more positive Pd center (NBO = +0.258 vs. +0.240 in Figure S7) with a stable coordination complex (**^E^12_(Coor6)_**) will undergo an easy bonding with C6 and C7, in contrast of the **^E^21_(Coor6)_** species in the same situation. Meanwhile, a larger HOMO/LUMO energy gap of the transition state structure (**^E^21_(TS3)_**) was identified as an indicator of previous instability that occurred in **A3**, which resulted in a high energy barrier at the same route and restricted complex activity to some extent (Figure 4d). A comparative analysis was conducted to figure out the overall situation, which suggested that the distortion in the catalytic moiety occurred after the insertion of TFP, whereas the geometric structures showed that E-inserted complexes were not significantly distorted (Figure 4c, Figures S6 and S7).

### 2.2. Effects of Electronic and Steric Parameters on the Polymerization Activity

**Figure 5:**
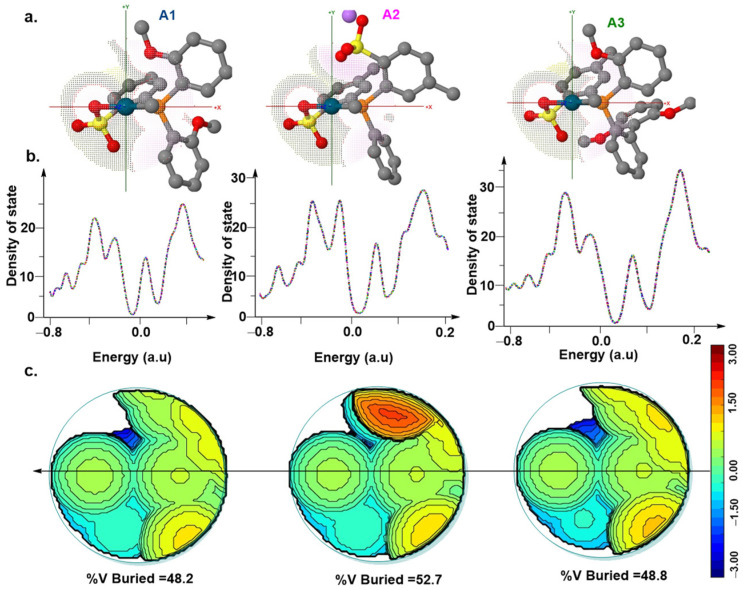
Steric and electronic state analysis. (**a**) Optimized orientation of used structures, (**b**) density of state graphs, and (**c**) steric maps.

The aforementioned findings prompted us to sterically examine the relevant complexes in order to confirm the potential electron-withdrawing substituent’s impact on structural variation and catalytic activity. To accomplish this, we analyzed the steric changes that occurred during the monomer insertion. In the steric map analysis of the catalysts prior to the insertion of any type of monomer, we found that **A2** is sterically more crowded with a %V_bur_ of 52.7 in contrast to **A1** and **A3** (48.2 and 48.8); after that, the chain initiation step was carried out and we observed the highest energy barrier (20.9 kcal mol^−1^) in a comparison of analog (**A1** 19.9 and **A3** 20.0 kcal mol^−1^) insertions at the same level in Figure 5 and Figure S2b. Our findings imply that steric bulk around the metal center is important for incoming TFP, and consequently, the energy barrier for ethylene insertion in the **A2** complex is higher and lowest for **A1** in an overall comparison.

Following that, the E-insertion in precursor **21_(P2)_** showed the highest energy barrier (24.2 kcal mol^−1^) with complex **A2**, and likewise, the steric analysis presented more crowded (%V_bur_ of **12/21_(P2)_** is 50.0 and 51.1) space around the Pd metal center compared to **12_(P2)_**. In the same context, ethylene insertion occurred in complex **A1** with energy barriers of 20.0 and 22.3 kcal mol^−1^, which was observed to be sterically least crowded (%V_bur_ of **12/21_(P2)_** is 42.4 and 46.7). Similarly, the steric map of **A3** shows that %V_bur_ of **12/21_(P2)_** is 47.4 and 47.7, with obtained energy barriers 21.4 kcal mol^−1^ (Figure 5 and Figure S9). These findings pointed out the interplay of sterics and the activities of the complexes; thus, an electronic state analysis was performed further.

In the computed data of the total density of states (TDOS) for each complex, it was shown that among the chosen complexes, complex **A3** is determined to be the most active in terms of electronic activity, with a peak density of state value of 35.5 at the highest level (**A1** is 25.6 and **A2** is 28.7). This analysis establishes that the relative tendency to insert ethylene and TFP can be affected by both steric and electronic factors, which ultimately renders the incorporation of TFP less advantageous; these results are consistent with earlier findings from late transition metal complexes [18,38]. 

Our results show that, in addition to the possible impact of electron-withdrawing substituents on structural variation and catalytic efficiency, the adverse steric impact is also a significant cause of the low activity. Although the possibility of β-F elimination has been reduced to minimize the activity when using the Pd complexes (**A1**, **A2**, and **A3**) for the insertion of the TFP monomer, it participated in improving the fluorine content during polymer chain formation. To deal with desirable features in the polymer sector, it still needs to be addressed with regard to a low incorporation and molecular weight. Precisely, a functionalized polyethylene synthesis and a property analysis will remain compelling areas of research in the future. A few special polar functionalized polyethylenes could eventually be commercialized as a result of rigorous and continuous study.

## 3. Computational Methods and Software Details

The Gaussian 16 program was used for all of the calculations [39]. B3PW91 functional was used to conduct geometric optimization and frequency analysis for all structures [40]. The effective core potentials (ECPs) of Hay and Wadt with a double-ζ valence basis set (LanL2DZ) were used for Pd atom [41,42,43], while the 6-31G* basis set was used for the rest of the atoms; such basis sets are denoted as BSI. In order to obtain accurate relative energies, single-point calculations of optimized structures were also carried out, including a higher-level dispersion-corrected calculation [44], the density functional method, B3PW91-D3, together with BSII. In BSII, the Stuttgart/Dresden ECP and associated basis sets were applied to the Pd atom, while 6-311G(d,p) was used for the rest of the atoms [45,46,47,48]. 

To approximate the experimental results, a designated solvent, toluene, was utilized. Thus, in this single-point calculation, the solvation effect of toluene was taken into account using the SMD model [49]. The relative free energy in the solution phase (ΔG, kcal/mol) was used to calculate the energy profiles of the insertion mechanism [50]. Using Multiwfn and VMD software (VMD for WIN32 version 1.9.3), a non-covalent interaction analysis (NCI) was carried out for a few transition states (TSs) to observe the weak, strong, and Vdw interactions between the catalysts and monomers [51,52]. Cavallo’s SambVca 2.0 program was also used to visualize the steric maps [53]. CLYview and Chemcraft were used to see the clear structural and geometrical parameters in detail [54].

## 4. Conclusions

In this study, three asymmetric catalysts (**A1**, **A2**, and **A3**) are used in combination with their unique electronic environments to produce linear copolymers of ethylene with polar olefins like VF and TFP. The executed computational study assesses that both the insertion modes (1,2/2,1-TFP insertion) contributed to the activity; however, during the chain initiation step, the 2,1-insertion was marginally favored due to the presence of additional fluorine atoms in the monomer moiety as compared to the one fluorine atom in the vinyl fluoride insertion case. A highly favorable 2,1-insertion during chain propagation step was observed as a result of less distorted complexes (A) and monomer (B) moieties, which induced the stability in the overall complex structure. The moderately crowded species (**A3**), evidenced by the steric distribution maps of the 1,2/2,1-insertion products, have shown greater incorporation rates for incoming monomers with moderate energy barriers and superior selectivity. These results demonstrate that steric implications are obvious, leading to narrow dihedral angles and improved catalytic stability, which encouraged the insertion of ethylene after 1,2-TFP insertion and ultimately increased the copolymerization activities for **A1** and **A3**. These findings imply that if the steric bulk is correctly managed, copolymer molecular weights can be enhanced. Thus, we are currently developing more catalytic systems by modifying electronic and steric factors that could lead to the creation of demanding plastic polymers with novel features for potential industrial applications.

## Data Availability

The data presented in this study are available upon request from the corresponding authors.

## Supplementary Materials

The following supporting information can be downloaded at: https://www.mdpi.com/article/10.3390/molecules28207148/s1.

## Sample Availability

Not available.

## References

[1] Kharitonov A., Taege R., Ferrier G., Teplyakov V., Syrtsova D., Koops G.-H. (2005). Direct fluorination—Useful tool to enhance commercial properties of polymer articles. J. Fluor. Chem..

[2] Kharitonov A., Moskvin Y.L., Teplyakov V., Le Roux J. (1999). Direct fluorination of poly (vinyl trimethylsilane) and poly (phenylene oxide). J. Fluor. Chem..

[3] Boz E., Nemeth A.J., Ghiviriga I., Jeon K., Alamo R.G., Wagener K.B. (2007). Precision ethylene/vinyl chloride polymers via condensation polymerization. Macromolecules.

[4] Boz E., Nemeth A.J., Alamo R.G., Wagener K.B. (2007). Precision ethylene/vinyl bromide polymers. Adv. Synth. Catal..

[5] Boz E., Wagener K.B., Ghosal A., Fu R., Alamo R.G. (2006). Synthesis and crystallization of precision ADMET polyolefins containing halogens. Macromolecules.

[6] Yang H., Islam M., Budde C., Rowan S.J. (2003). Ring-opening metathesis polymerization as a route to controlled copolymers of ethylene and polar monomers: Synthesis of ethylene–vinyl chloride-like copolymers. J. Polym. Sci. Part A Polym. Chem..

[7] Sun Y., Wang C., Tanaka R., Shiono T., Cai Z. (2019). Copolymerization of Ethylene and Fluoroalkylnorbornene Using Highly Active ansa-(Fluorenyl)(amido)titanium-Based Catalysts. Macromol. Chem. Phys..

[8] Nakamura A., Anselment T.M., Claverie J., Goodall B., Jordan R.F., Mecking S., Rieger B., Sen A., Van Leeuwen P.W., Nozaki K. (2013). Ortho-phosphinobenzenesulfonate: A superb ligand for palladium-catalyzed coordination–insertion copolymerization of polar vinyl monomers. Acc. Chem. Res..

[9] Chen E.Y.-X. (2009). Coordination polymerization of polar vinyl monomers by single-site metal catalysts. Chem. Rev..

[10] Drent E., van Dijk R., van Ginkel R., van Oort B., Pugh R.I. (2002). Palladium catalysed copolymerisation of ethene with alkylacrylates: Polar comonomer built into the linear polymer chain. Chem. Commun..

[11] Vela J., Lief G.R., Shen Z., Jordan R.F. (2007). Ethylene polymerization by palladium alkyl complexes containing bis (aryl) phosphino-toluenesulfonate ligands. Organometallics.

[12] Guironnet D., Roesle P., Rünzi T., Göttker-Schnetmann I., Mecking S. (2009). Insertion polymerization of acrylate. J. Am. Chem. Soc..

[13] Weng W., Shen Z., Jordan R.F. (2007). Copolymerization of ethylene and vinyl fluoride by (phosphine-sulfonate) Pd (Me)(py) catalysts. J. Am. Chem. Soc..

[14] Wada S., Jordan R.F. (2017). Olefin Insertion into a Pd–F Bond: Catalyst Reactivation Following β-F Elimination in Ethylene/Vinyl Fluoride Copolymerization. Angew. Chem. Int. Ed..

[15] Lanzinger D., Giuman M.M., Anselment T.M., Rieger B. (2014). Copolymerization of ethylene and 3, 3, 3-trifluoropropene using (phosphine-sulfonate) Pd (Me)(DMSO) as catalyst. ACS Macro Lett..

[16] Borkar S., Newsham D.K., Sen A. (2008). Copolymerization of Ethene with Styrene Derivatives, Vinyl Ketone, and Vinylcyclohexane Using a (Phosphine–sulfonate) palladium (II) System: Unusual Functionality and Solvent Tolerance. Organometallics.

[17] Rünzi T., Mecking S. (2014). Saturated Polar-Substituted Polyethylene Elastomers from Insertion Polymerization. Adv. Funct. Mater..

[18] Wucher P., Roesle P., Falivene L., Cavallo L., Caporaso L., Göttker-Schnetmann I., Mecking S. (2012). Controlled acrylate insertion regioselectivity in diazaphospholidine-sulfonato palladium (II) complexes. Organometallics.

[19] Von Schenck H., Strömberg S., Zetterberg K., Ludwig M., Åkermark B., Svensson M. (2001). Insertion Aptitudes and Insertion Regiochemistry of Various Alkenes Coordinated to Cationic (σ-R)(diimine) palladium (II)(R = –CH3,–C6H5). A Theoretical Study. Organometallics.

[20] Shen Z., Jordan R.F. (2010). Copolymerization of ethylene and vinyl fluoride by (phosphine-bis (arenesulfonate)) PdMe (pyridine) catalysts: Insights into inhibition mechanisms. Macromolecules.

[21] Wu F., Foley S.R., Burns C.T., Jordan R.F. (2005). Acrylonitrile insertion reactions of cationic palladium alkyl complexes. J. Am. Chem. Soc..

[22] Groux L.F., Weiss T., Reddy D.N., Chase P.A., Piers W.E., Ziegler T., Parvez M., Benet-Buchholz J. (2005). Insertion of acrylonitrile into palladium methyl bonds in neutral and anionic Pd (II) complexes. J. Am. Chem. Soc..

[23] Mehmood A., Xu X., Kang X., Luo Y. (2020). Origin of different chain-end microstructures in ethylene/vinyl halide copolymerization catalysed by phosphine–sulfonate palladium complexes. New J. Chem..

[24] Mehmood A., Mahmood A., Xu X., Raza W., Ahmed S., Ullah N., Luo Y., Tian X. (2023). Mechanistic study to reveal steric and electronic aspects involved in the formation of microstructures during Pd-catalyzed olefin/divinyl formal copolymerization: Reactivity to catalyst choice. Phys. Chem. Chem. Phys..

[25] Mehmood A., Xu X., Raza W., Kukkar D., Kim K.-H., Luo Y. (2021). Computational study of the copolymerization mechanism of ethylene with methyl 2-acetamidoacrylate catalyzed by phosphine-sulfonate palladium complexes. New J. Chem..

[26] Mehmood A., Xu X., Raza W., Kim K.-H., Luo Y. (2020). Mechanistic studies for palladium catalyzed copolymerization of ethylene with vinyl ethers. Polymers.

[27] Xu X., Luo G., Mehmood A., Zhao Y., Zhou G., Hou Z., Luo Y. (2018). Theoretical mechanistic studies on redox-switchable polymerization of trimethylene carbonate catalyzed by an indium complex bearing a ferrocene-based ligand. Organometallics.

[28] Piche L., Daigle J.C., Rehse G., Claverie J.P. (2012). Structure–Activity Relationship of Palladium Phosphanesulfonates: Toward Highly Active Palladium-Based Polymerization Catalysts. Chem. A Eur. J..

[29] Skupov K.M., Marella P.R., Simard M., Yap G.P., Allen N., Conner D., Goodall B.L., Claverie J.P. (2007). Palladium aryl sulfonate phosphine catalysts for the copolymerization of acrylates with ethene. Macromol. Rapid Commun..

[30] Shen Z., Jordan R.F. (2010). Self-assembled tetranuclear palladium catalysts that produce high molecular weight linear polyethylene. J. Am. Chem. Soc..

[31] Rünzi T., Fröhlich D., Mecking S. (2010). Direct synthesis of ethylene− acrylic acid copolymers by Insertion polymerization. J. Am. Chem. Soc..

[32] Nakano R., Chung L.W., Watanabe Y., Okuno Y., Okumura Y., Ito S., Morokuma K., Nozaki K. (2016). Elucidating the key role of phosphine–sulfonate ligands in palladium-catalyzed ethylene polymerization: Effect of ligand structure on the molecular weight and linearity of polyethylene. ACS Catal..

[33] Rezabal E., Ugalde J.M., Frenking G. (2017). The trans Effect in Palladium Phosphine Sulfonate Complexes. J. Phys. Chem. A.

[34] Lefebvre C., Rubez G., Khartabil H., Boisson J.-C., Contreras-García J., Hénon E. (2017). Accurately extracting the signature of intermolecular interactions present in the NCI plot of the reduced density gradient versus electron density. Phys. Chem. Chem. Phys..

[35] Chen S., Huang X., Meggers E., Houk K. (2017). Origins of Enantioselectivity in Asymmetric Radical Additions to Octahedral Chiral-at-Rhodium Enolates: A Computational Study. J. Am. Chem. Soc..

[36] Bickelhaupt F.M., Houk K.N. (2017). Analyzing reaction rates with the distortion/interaction-activation strain model. Angew. Chem. Int. Ed..

[37] Zou C., Pang W., Chen C. (2018). Influence of chelate ring size on the properties of phosphine-sulfonate palladium catalysts. Sci. China Chem..

[38] Tan C., Zou C., Chen C. (2022). Material Properties of Functional Polyethylenes from Transition-Metal-Catalyzed Ethylene–Polar Monomer Copolymerization. Macromolecules.

[39] Frisch M., Trucks G., Schlegel H., Scuseria G., Robb M., Cheeseman J., Scalmani G., Barone V., Petersson G., Nakatsuji H. (2016). Gaussian 16 Rev. C. 01.

[40] Avcı D., Bahçeli S., Tamer Ö., Atalay Y. (2015). Comparative study of DFT/B3LYP, B3PW91, and HSEH1PBE methods applied to molecular structures and spectroscopic and electronic properties of flufenpyr and amipizone. Can. J. Chem..

[41] Hay P.J., Wadt W.R. (1985). Ab initio effective core potentials for molecular calculations. Potentials for the transition metal atoms Sc to Hg. J. Chem. Phys..

[42] Wadt W.R., Hay P.J. (1985). Ab initio effective core potentials for molecular calculations. Potentials for main group elements Na to Bi. J. Chem. Phys..

[43] Friesner R.A., Murphy R.B., Beachy M.D., Ringnalda M.N., Pollard W.T., Dunietz B.D., Cao Y. (1999). Correlated ab initio electronic structure calculations for large molecules. J. Phys. Chem. A.

[44] Grimme S., Antony J., Ehrlich S., Krieg H. (2010). A consistent and accurate ab initio parametrization of density functional dispersion correction (DFT-D) for the 94 elements H-Pu. J. Chem. Phys..

[45] Martin J.M., Sundermann A. (2001). Correlation consistent valence basis sets for use with the Stuttgart–Dresden–Bonn relativistic effective core potentials: The atoms Ga–Kr and In–Xe. J. Chem. Phys..

[46] Lee C., Yang W., Parr R.G. (1988). Development of the Colle-Salvetti correlation-energy formula into a functional of the electron density. Phys. Rev. B.

[47] Steinbrenner U., Bergner A., Dolg M., Stoll H. (1994). On the transferability of energy adjusted pseudopotentiais: A calibration study for XH4 (X = C, Si, Ge, Sn, Pb). Mol. Phys..

[48] Kaupp M., Schleyer P.v.R., Stoll H., Preuss H. (1991). Pseudopotential approaches to Ca, Sr, and Ba hydrides. Why are some alkaline earth MX2 compounds bent?. J. Chem. Phys..

[49] Marenich A.V., Cramer C.J., Truhlar D.G. (2009). Universal solvation model based on solute electron density and on a continuum model of the solvent defined by the bulk dielectric constant and atomic surface tensions. J. Phys. Chem. B.

[50] Kozuch S. (2012). A refinement of everyday thinking: The energetic span model for kinetic assessment of catalytic cycles. WIREs Comput. Mol. Sci..

[51] Lu T., Chen F. (2012). Multiwfn: A multifunctional wavefunction analyzer. J. Comput. Chem..

[52] Humphrey W., Dalke A., Schulten K. (1996). VMD: Visual molecular dynamics. J. Mol. Graph..

[53] Falivene L., Credendino R., Poater A., Petta A., Serra L., Oliva R., Scarano V., Cavallo L. (2016). SambVca 2. A web tool for analyzing catalytic pockets with topographic steric maps. Organometallics.

[54] Zhurko G., Zhurko D. Chemcraft—Graphical Software for Visualization of Quantum Chemistry Computations. Version 1.8, 2009, Build 654. https://www.chemcraftprog.com.

