# Supplementary material for: Mechanistic Study on Steric Activity Interplay of Olefin/Polar Monomers for Industrially Selective Late Transition Metal Catalytic Reactions
Source: Molecules. 2023 Oct 18;28(20):7148. doi: 10.3390/molecules28207148 (PMC10609194; doi:10.3390/molecules28207148)
Supplement: Supplementary file 1 [file molecules-28-07148-s001.zip › molecules-2647198-supplementary.pdf]

Supporting information

Mechanistic Study on Steric Activity Interplay of Olefin/Polar Monomers for Industrially Selective Late Transition Metal Catalytic Reactions

Andleeb Mehmood [1], Ayyaz Mahmood [1], Najla AlMasoud [2], Arzoo Hassan [1], Taghrid S. Alomar[2], Zeinhom M. El-Bahy[3], Nadeem Raza[4], Xiaoqing Tian[1,*], and Naeem Ullah [1,*]

[1] College of Physics and Optoelectronic Engineering, Shenzhen University, Shenzhen 518000, China
[2] Department of Chemistry, College of Science, Princess Nourah bint Abdulrahman University, P.O. Box 84428, Riyadh 11671, Saudi Arabia
[3] Department of Chemistry, Faculty of Science, Al-Azhar University, Nasr City, Cairo 11884, Egypt
[4] Chemistry Department, Imam Mohammad Ibn Saud Islamic University (IMSIU), Riyadh 11623, Saudi Arabia
* Correspondence: xqtian@szu.edu.cn (X .T); naeeman259@szu.edu.cn(N.U.)

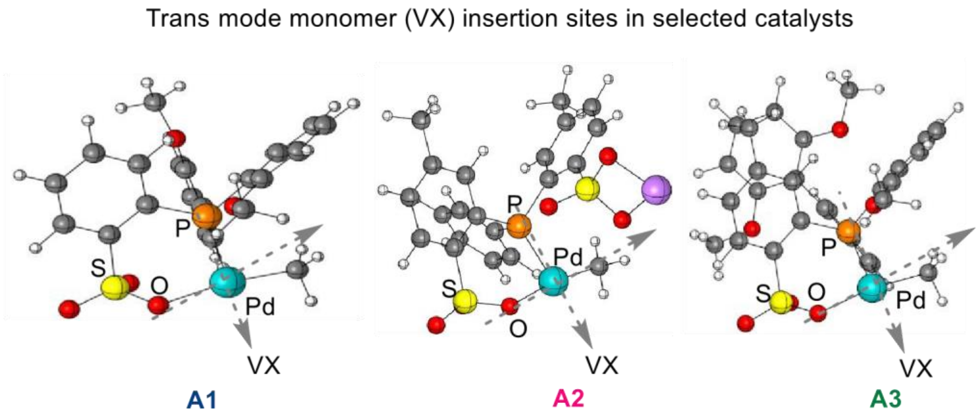

Figure. S1 The favorable coordination manners (trans) for 1st step coordination in Pd complexes **A1** (blue), **A2** (pink), and **A3** (green).

The asymmetric nature of the phosphine sulfonate-based catalyst causes a monomer to coordinate with the metal center from two positions, namely, cis and trans, (as shown in Fig. S1) Previous research has shown that the polymerization reaction by phosphine-sulfonate-based complexes begins with viable isomerization from the more stable trans complex to its' cis-isomer, followed by the more kinetically preferred cis-isomer insertion [1, 2]. As a result, a thorough mechanism of ETFP copolymerization was investigated using the species A_{trans}.

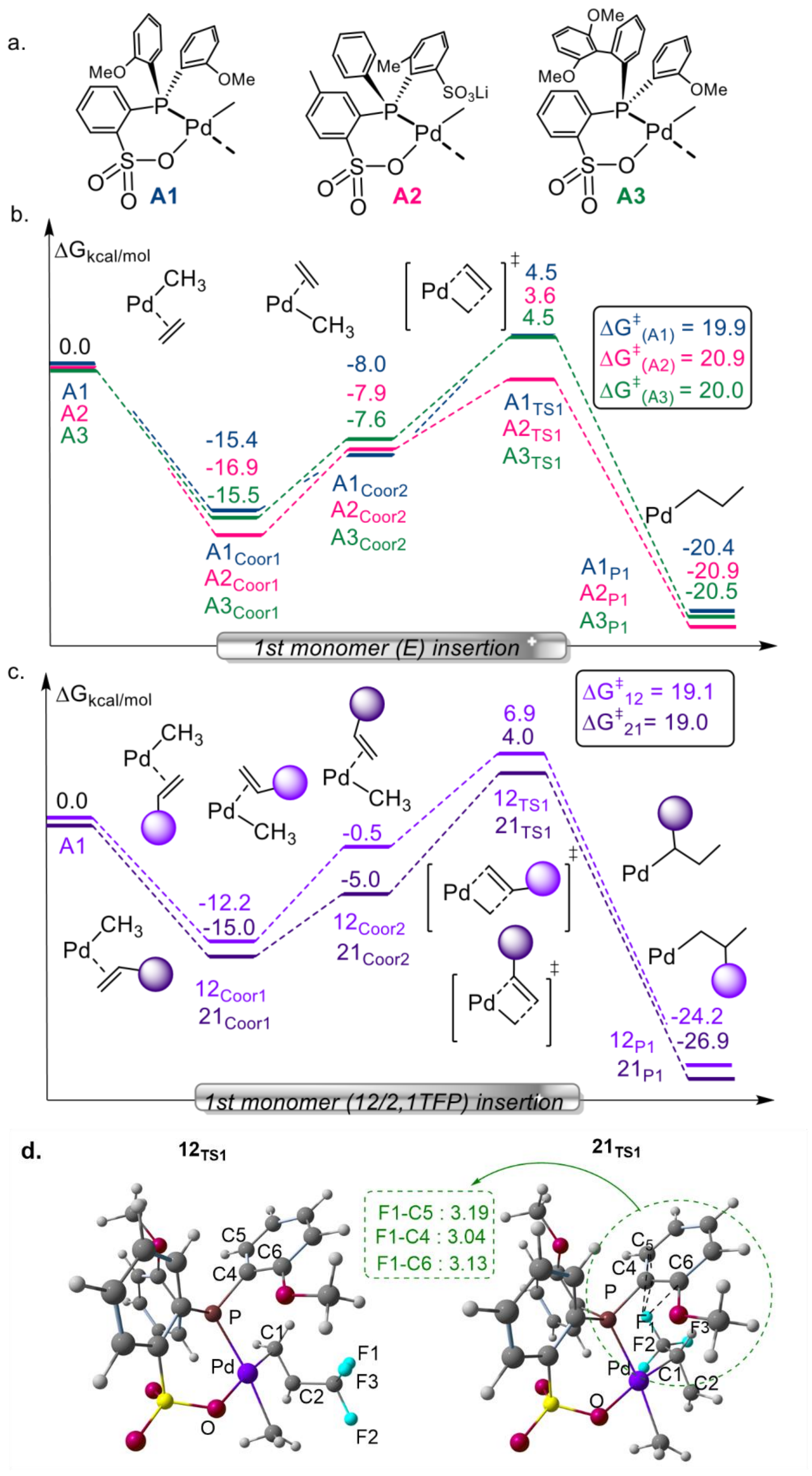
a.
A1
A2
A3
b.
ΔG kcal/mol
0.0
-15.4
-16.9
-15.5
-8.0
-7.9
-7.6
4.5
3.6
4.5
-20.4
-20.9
-20.5
ΔG‡(A1) = 19.9
ΔG‡(A2) = 20.9
ΔG‡(A3) = 20.0
A1 A2 A3
A1Coor1 A2Coor1 A3Coor1
A1Coor2 A2Coor2 A3Coor2
A1TS1 A2TS1 A3TS1
A1P1 A2P1 A3P1
1st monomer (E) insertion
c.
ΔG kcal/mol
0.0
-12.2
-15.0
-0.5
-5.0
6.9
4.0
-24.2
-26.9
ΔG‡12 = 19.1
ΔG‡21= 19.0
A1
12Coor1 21Coor1
12Coor2 21Coor2
12TS1 21TS1
12P1 21P1
1st monomer (12/2,1TFP) insertion
d.
12TS1
21TS1
F1-C5 : 3.19
F1-C4 : 3.04
F1-C6 : 3.13

Figure. S2 a. Types of catalysts used, **b.** calculated energy profiles for E- insertion as 1st monomer insertion (chain initiation) mediated by **A1**, **A2**, and **A3**, and **c.** calculated energy profiles for TFP-insertion as 1st monomer insertion in Pd-Me bond of complex **A1**. **d.** Geometric analysis of transition state structure of TFP-1,2 and 2,1-insertion step. Free energies are relative to the energy sum of catalysts (**A1**, **A2**, and **A3**) and monomers (E). All energies are calculated in kcal mol^{-1}.

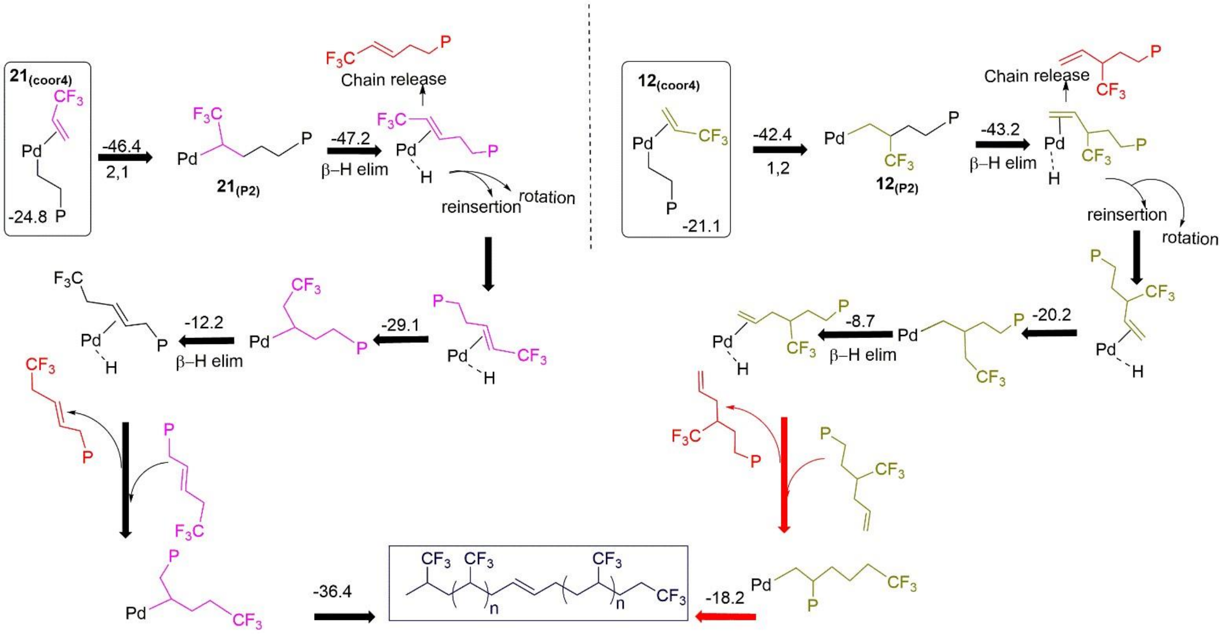

Figure. S3 Calculated energy profiles for chain termination reaction pathway as proposed TFP insertion for copolymerization mediated by **A1**. All energies are calculated in kcal mol^{-1}.

A polymeric olefin with an internal double bond is created during chain release. Chain walking results in a saturated terminating end group if TFP is the final monomer introduced. This suggests that chain walking is favorably promoted in the case of 2,1-insertion of TFP as opposed to direct chain release after β-H transfer to the palladium center.

Table. S1 Energy decomposition analysis (EDA) for E/TFP insertion as a second monomer for copolymerization mediated by **A1**, **A2**, and **A3**. All energies are calculated in kcal mol^{-1}.

Catalysts with monomer insertion manners	$\Delta E_{def\,(A)}$	$\Delta E_{def\,(B)}$	ΔE_{int}	ΔE	$\Delta G^{\ddagger}$
A1(E2)	39.29929	14.34223	-33.07	20.57	20.55
A2(E2)	33.81285	14.9998	-32.06	16.74	16.94
A3(E2)	34.51302	14.91214	-31.32	18.10	17.91

A1(E-12-tfp)	41.8867	21.17205	-38.33	24.71	22.74
A2(E-12-tfp)	51.01947	16.24986	-47.44	19.82	19.53
A3(E-12-tfp)	33.82684	18.57284	-34.52	17.87	17.84
A1(E-21-tfp)	38.77682	14.39005	-33.21	19.95	19.51
A2(E-21-tfp)	30.63821	14.74202	-34.80	10.57	11.02
A3(E-21-tfp)	31.60207	15.20926	-34.11	12.69	15.34

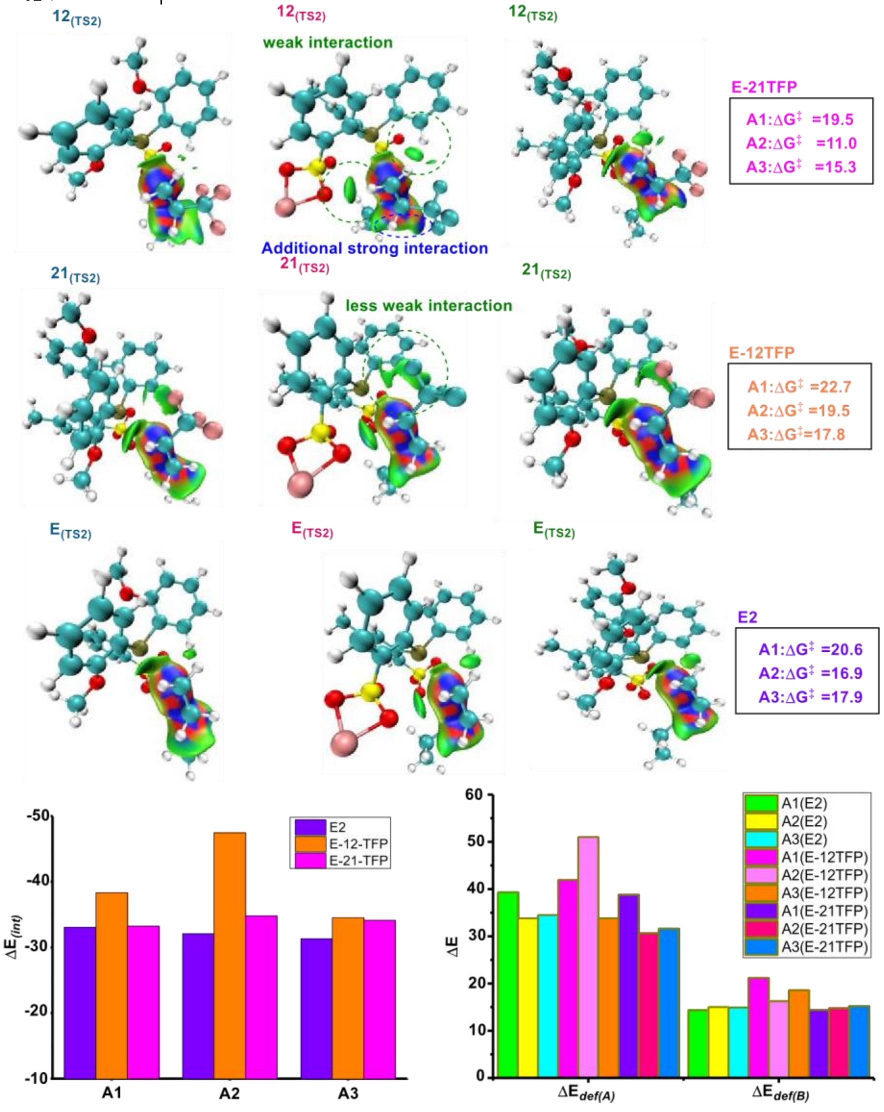

Figure. S4 Graphical presentation of energy decomposition analysis (EDA) for E/TFP insertion as second monomer for copolymerization mediated by **A1**, **A2**, and **A3**. All energies are calculated in kcal mol^{-1}.

For energy decomposition analysis (EDA), energies of two fragments, the monomer moiety (A) and the monomer (B), in the TS geometries were analyzed using single-point calculations. The

fragments' single-point energies and the energy of TS were utilized to calculate the interaction energy ΔE_{int}. These energies, coupled with the energies of the respective fragments in their ideal geometry, allow for the determination of the two fragments' deformation energies, $\Delta E_{def(A)}$ and $\Delta E_{def(B)}$. When the energy of the TS, ΔE_{TS}, is compared to the energy of the two separated pieces, the relationship $\Delta E_{TS} = \Delta E_{int} + \Delta E_{def(A)} + \Delta E_{def(B)}$ holds.

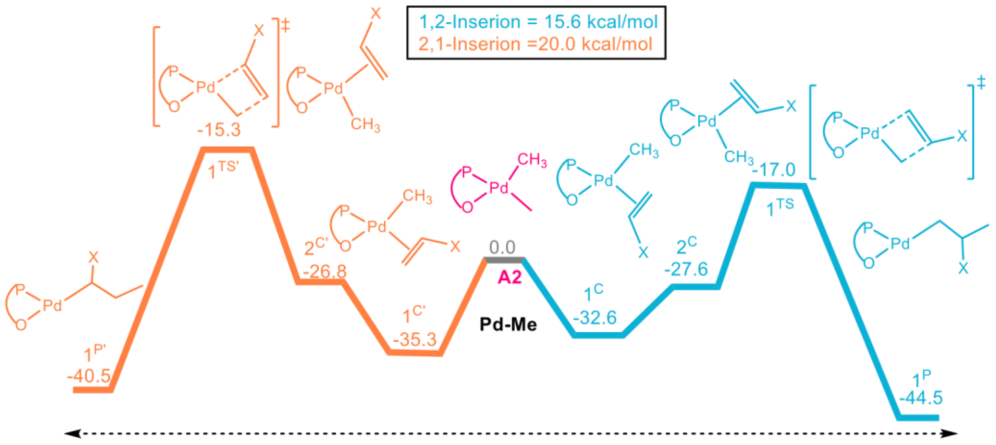

Figure. S5 Calculated energy profiles for Vinyl fluoride (VF)- insertion in Pd-Me bond as 1st monomer mediated by **A2.** All energies are calculated in kcal mol^{-1}.

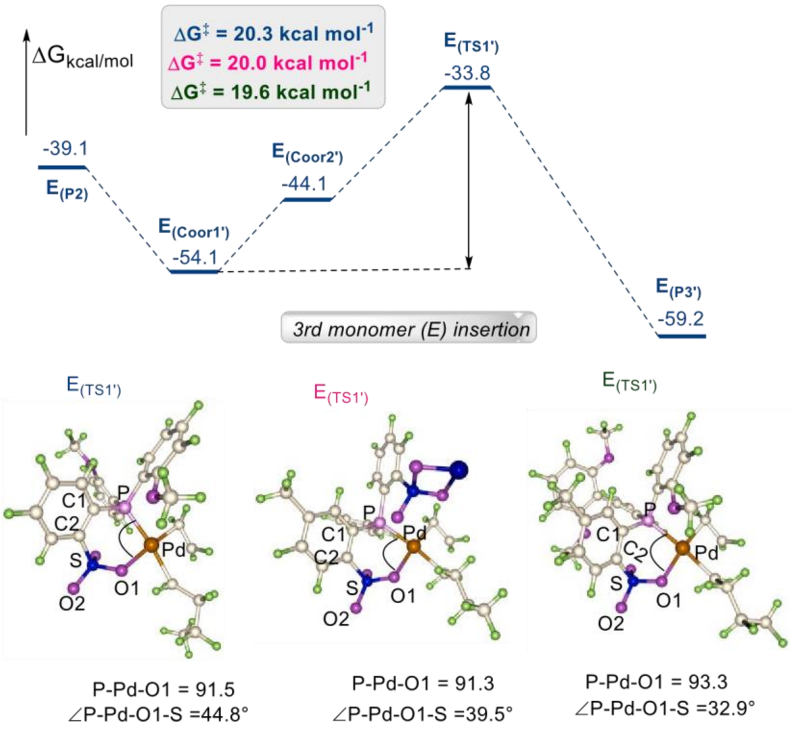

Figure. S6 Calculated energy profiles for E- insertion as third monomer mediated by **A1** is shown but energy barriers of **A2**, and **A3** are also given with geometric structures for all complexes in the case of third ethylene insertion. All energies are calculated in kcal mol^{-1}.

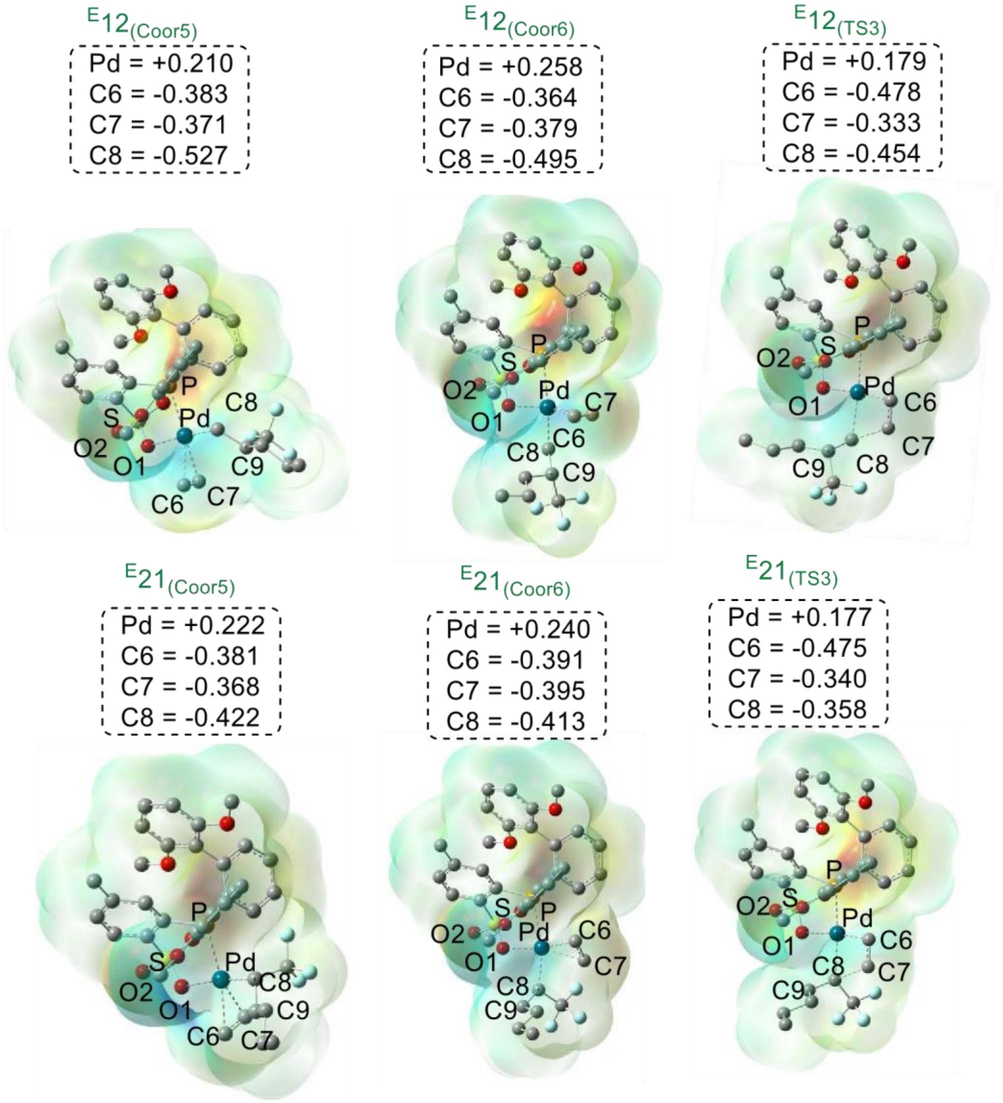

Figure. S7 Natural bond orbital analysis (NBO charges) for copolymerization key structures using complex **A3**.

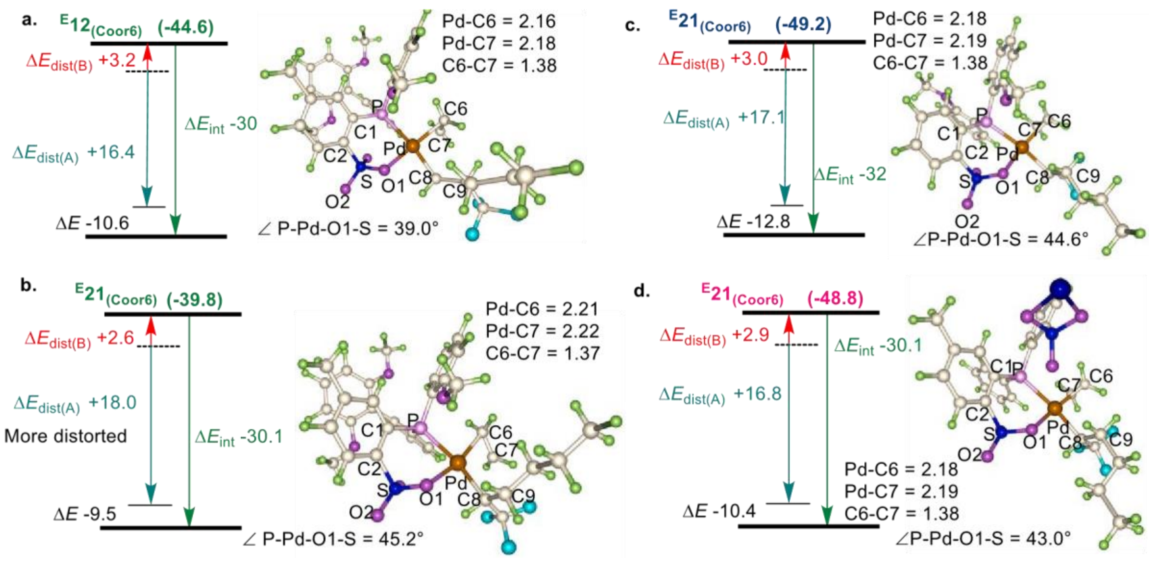

Figure. S8 Energy decomposition analysis (EDA) for E-TFP-E π-coordination insertion for copolymerization mediated by **A1**, **A2**, and **A3**. All energies are calculated in kcal mol^{-1}.

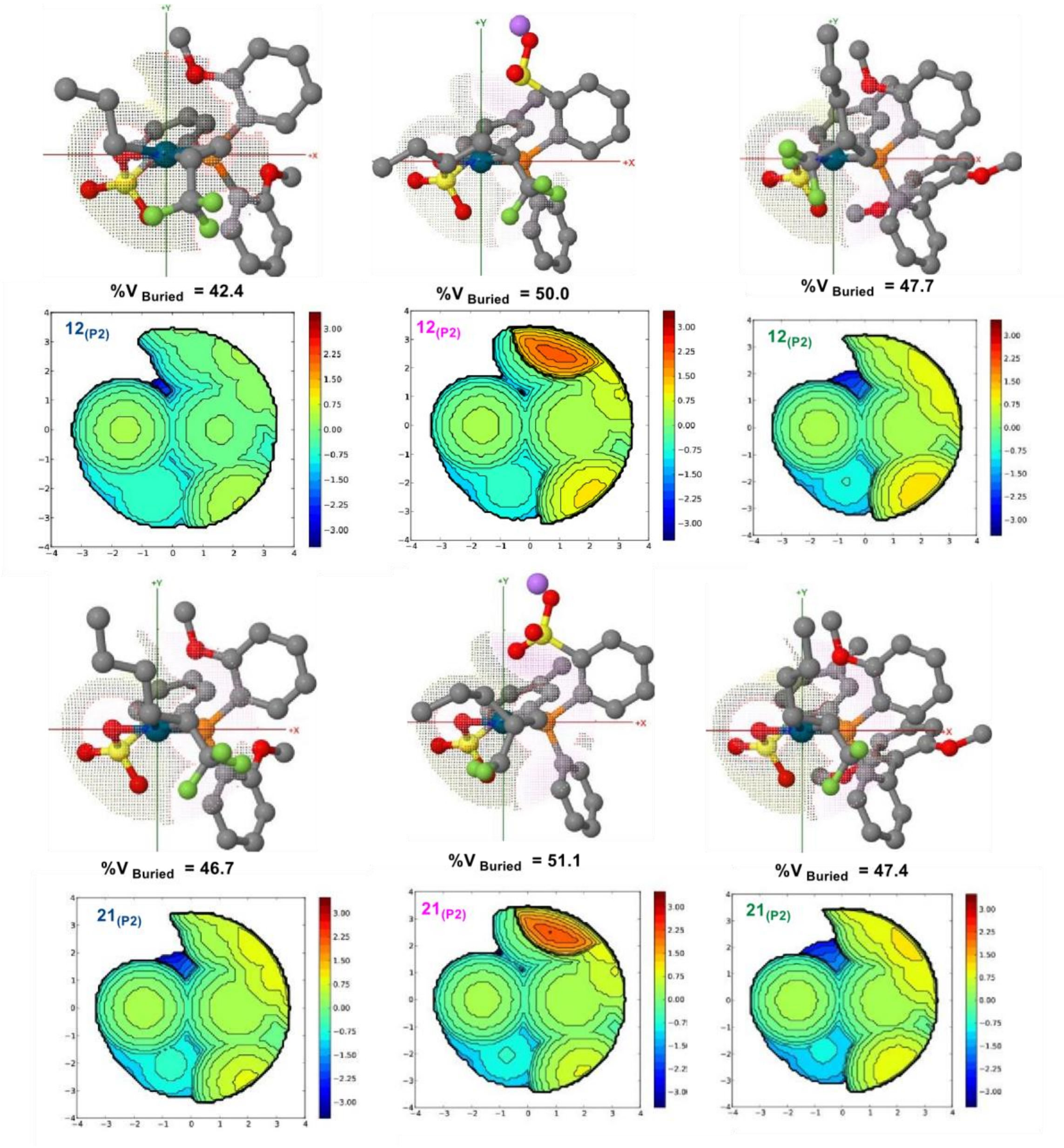

Figure. S9 Steric map analysis for key precursors using complex **A1, A2, and A3**.

References

1. T. Rünzi, D. Fröhlich and S. Mecking, *Journal of the American Chemical Society*, 2010, **132**, 17690-17691.
2. R. Nakano, L. W. Chung, Y. Watanabe, Y. Okuno, Y. Okumura, S. Ito, K. Morokuma and K. Nozaki, *ACS Catalysis*, 2016, **6**, 6101-6113.

50

Cartesian coordinates for all-important optimized structures.

50

A1trans

Pd	-0.881591707	-0.952753389	-1.833492221
P	0.292218945	0.064684191	-0.236591101
C	-0.549287011	-0.186502189	1.381260022
C	-1.949663095	-0.234784793	1.507883030
S	-3.084180254	-0.065552266	0.105899925
O	-2.698484717	-1.255199190	-0.776570142
O	-2.737566780	1.218462980	-0.528512123
O	-4.434787292	-0.219346857	0.646804964
C	-2.532630058	-0.426894058	2.758939127
C	-1.739074929	-0.585814730	3.892267215
C	0.235524123	-0.360185866	2.528156108
C	0.469171191	1.857108402	-0.528831124
C	-0.077123084	2.401091210	-1.695532214
H	-0.580719838	1.735269944	-2.389269268
C	-0.014663665	3.766907342	-1.956274234
H	-0.451144869	4.166329186	-2.866397305
C	0.589014019	4.609347663	-1.028932164
H	0.639453565	5.679516747	-1.212979175
C	1.128058281	4.099410855	0.150826928
H	1.588319025	4.773733104	0.864497980
C	1.068682863	2.727005725	0.409446948
O	1.576612148	2.154353898	1.527749032
C	2.087315830	2.989285184	2.549243112
H	2.972155659	3.543604607	2.210699083
H	2.370984136	2.321657254	3.364218172
H	1.326243469	3.694211913	2.905379136
C	1.971126371	-0.626179143	-0.062366088
C	3.118055134	0.131858407	-0.308915107
H	3.016648675	1.186271445	-0.543892123
C	4.386152474	-0.445259094	-0.265137103
H	5.265893283	0.161980330	-0.456727118
C	4.511461064	-1.800666144	0.023897918
H	5.494794358	-2.262188760	0.064629921
C	3.383016320	-2.586627685	0.254611936
H	3.497422780	-3.644040719	0.466979952
C	2.112146975	-2.007839186	0.201442932
O	0.959165176	-2.688854734	0.380163946
C	1.007324777	-4.080440819	0.636569967
H	1.483380044	-4.621693654	-0.190579098
H	-0.031922167	-4.397198287	0.729268973
H	1.540298910	-4.294076606	1.571600035

C	-0.352114837	-0.557355133	3.775107202
C	0.712026372	-0.850380701	-3.073270318
H	1.365366786	-1.707753484	-2.876331306
H	0.235363375	-0.948388910	-4.062396393
H	1.304980021	0.068189625	-3.054451315
H	-2.205711900	-0.739168942	4.861952286
H	1.316870194	-0.336766401	2.442194105
H	-3.615983127	-0.458710524	2.815797133
H	0.277503267	-0.689388873	4.651466272

52

A2trans

Pd	-0.686166000	-0.677530000	-2.014778000
P	-0.110723000	0.450637000	-0.198187000
C	-0.828570000	-0.300491000	1.311018000
C	-2.097568000	-0.903049000	1.296680000
S	-3.132362000	-1.003693000	-0.184501000
O	-2.270473000	-1.806985000	-1.160253000
O	-3.294454000	0.392984000	-0.629899000
O	-4.333942000	-1.743884000	0.201410000
C	-2.617092000	-1.430514000	2.475361000
C	-1.891645000	-1.366930000	3.661331000
C	-0.110247000	-0.251367000	2.510775000
C	-0.735400000	2.173229000	-0.198583000
C	-0.972463000	2.829029000	-1.411550000
H	-0.808302000	2.302627000	-2.346272000
C	-1.453517000	4.135962000	-1.420132000
H	-1.644435000	4.631794000	-2.368115000
C	-1.711588000	4.795413000	-0.219634000
H	-2.098506000	5.811156000	-0.228013000
C	-1.491624000	4.142180000	0.992442000
H	-1.710159000	4.643861000	1.931550000
C	-1.008384000	2.836034000	1.006114000
C	1.713701000	0.606439000	0.114722000
C	2.219872000	1.889018000	0.361562000
H	1.532095000	2.728237000	0.378407000
C	3.576738000	2.144472000	0.596139000
C	4.455807000	1.063240000	0.596098000
H	5.512840000	1.220072000	0.798104000
C	3.989245000	-0.223243000	0.344823000
H	4.670191000	-1.067236000	0.371888000
C	2.640171000	-0.460505000	0.087828000
C	-0.624840000	-0.779502000	3.698608000
C	0.182758000	-0.748026000	4.969434000
H	0.715184000	-1.696654000	5.117091000
H	0.933757000	0.048647000	4.951198000

H	-0.455762000	-0.593095000	5.845928000
H	-2.316216000	-1.787234000	4.570678000
H	0.881274000	0.194333000	2.525068000
H	-3.598231000	-1.893278000	2.438633000
H	-0.862459000	2.326457000	1.954144000
C	4.059075000	3.551124000	0.827591000
H	3.311291000	4.150363000	1.357009000
H	4.985492000	3.566516000	1.410469000
H	4.262474000	4.054575000	-0.126545000
S	2.198399000	-2.170786000	-0.262102000
O	0.911715000	-2.433889000	0.367268000
O	2.242586000	-2.350497000	-1.768890000
O	3.362907000	-3.006682000	0.235403000
Li	3.616934000	-3.602444000	-1.536763000
C	0.731660000	0.262399000	-3.113957000
H	1.288487000	1.101590000	-2.688641000
H	1.432500000	-0.542723000	-3.361373000
H	0.182303000	0.607990000	-4.002996000

67

A3trans

Pd	-2.845312115	0.519507120	0.728837063
P	-0.639813950	0.518313008	0.332513031
C	-0.322359987	-0.714315101	-0.994837072
C	-1.038221103	-1.922321158	-1.086812079
S	-2.260910225	-2.452562138	0.135911016
O	-3.359418253	-1.400200000	-0.018540995
O	-1.587513168	-2.353206162	1.442272116
O	-2.722482329	-3.774996213	-0.292440016
C	-0.826265130	-2.766286235	-2.172536159
C	0.080319956	-2.421754253	-3.173119239
C	0.584286096	-0.389930123	-2.008234148
C	0.359058113	0.243029936	1.853414146
C	-0.327260927	0.496555990	3.051275240
H	-1.389982995	0.712504060	3.008352234
C	0.314228120	0.454156955	4.283871333
H	-0.249213913	0.649660997	5.192052403
C	1.669556210	0.147872862	4.338245338
H	2.190827244	0.110736833	5.291436410
C	2.352761245	-0.141775194	3.162291245
H	3.402260311	-0.420110269	3.201154250
C	1.726268200	-0.116129161	1.908404151
C	-0.086683826	2.123776100	-0.343703020
C	0.855150286	2.918878114	0.312924030
H	1.353007307	2.533403059	1.195072097
C	1.173773374	4.193766196	-0.156062006

H	1.907762465	4.796331206	0.370875034
C	0.541885351	4.684735265	-1.293142093
H	0.781818422	5.676394337	-1.668575121
C	-0.413489759	3.918909257	-1.961287146
H	-0.904990777	4.319932313	-2.841017212
C	-0.739181849	2.646330177	-1.486240109
O	-1.671610963	1.846302163	-2.048832150
C	-2.380690993	2.301245231	-3.185867235
H	-2.946712991	3.214882330	-2.964668218
H	-3.071876084	1.497429208	-3.441442256
H	-1.704091932	2.484290214	-4.030254299
C	0.802021070	-1.229609199	-3.105919231
C	1.798556167	-0.850331222	-4.169110314
H	1.712565128	-1.499024264	-5.046659377
H	1.658590208	0.185545866	-4.499596339
H	2.823446242	-0.933227278	-3.787641284
H	0.221112933	-3.089365313	-4.020776301
H	1.140514187	0.541202918	-1.945246143
H	-1.398337222	-3.687565272	-2.222355164
C	2.564333242	-0.567003236	0.757747064
C	2.484399166	-1.915241338	0.354108033
C	3.567648358	0.243089773	0.202366021
C	3.344612207	-2.422279419	-0.627553041
C	4.422990397	-0.251013308	-0.790573056
C	4.297174320	-1.579293404	-1.189460082
H	3.279119146	-3.457637493	-0.940986064
H	5.188728487	0.375305701	-1.233145088
H	4.970942352	-1.969992466	-1.948370141
O	3.653129428	1.505982866	0.705484059
O	1.551643057	-2.642353347	0.997193081
C	4.663551548	2.356354877	0.205881022
H	4.530089548	2.559594904	-0.864734062
H	4.570139588	3.291115954	0.761925067
H	5.663929620	1.936145797	0.373736035
C	1.375676974	-4.005295437	0.659426058
H	0.530426890	-4.342233424	1.259126103
H	1.131796950	-4.124083437	-0.402885025
H	2.271744013	-4.592697530	0.901268073
C	-2.760831015	2.412328262	1.432289113
H	-2.988975997	3.070955321	0.585279049
H	-3.583518077	2.427493302	2.165745174
H	-1.844567927	2.759042243	1.915744151

56

A1(P1)

Pd	1.876024000	0.482047000	-0.455568000

P	-0.308364000	0.147012000	-0.113806000
C	-0.539998000	-1.082249000	1.236478000
C	0.337187000	-2.166107000	1.425988000
S	1.782771000	-2.474588000	0.377883000
O	2.631308000	-1.222766000	0.599071000
O	1.261099000	-2.543002000	-0.999603000
O	2.435685000	-3.667589000	0.918963000
C	0.119916000	-3.067778000	2.466256000
C	-0.955423000	-2.900025000	3.335057000
C	-1.609677000	-0.920256000	2.125615000
C	-1.182514000	-0.457831000	-1.598522000
C	-0.449515000	-0.626416000	-2.777271000
H	0.609539000	-0.388621000	-2.763553000
C	-1.041379000	-1.122493000	-3.935460000
H	-0.449369000	-1.250573000	-4.836380000
C	-2.386971000	-1.473084000	-3.914303000
H	-2.863710000	-1.869512000	-4.807230000
C	-3.140559000	-1.332591000	-2.750300000
H	-4.186468000	-1.618931000	-2.752185000
C	-2.545380000	-0.830354000	-1.589329000
O	-3.212773000	-0.658202000	-0.422409000
C	-4.547278000	-1.116892000	-0.326499000
H	-5.212893000	-0.562954000	-1.001109000
H	-4.850033000	-0.935791000	0.705977000
H	-4.616491000	-2.190113000	-0.542225000
C	-1.155679000	1.673196000	0.419181000
C	-2.141598000	2.289272000	-0.355065000
H	-2.475461000	1.808305000	-1.268638000
C	-2.697622000	3.510693000	0.022717000
H	-3.465884000	3.970706000	-0.591849000
C	-2.260285000	4.130021000	1.189418000
H	-2.688874000	5.080173000	1.497963000
C	-1.262986000	3.549056000	1.972127000
H	-0.924868000	4.050123000	2.872685000
C	-0.700229000	2.329720000	1.585618000
O	0.290878000	1.703823000	2.258101000
C	0.811526000	2.293772000	3.434639000
H	1.246056000	3.279886000	3.227783000
H	1.592891000	1.616538000	3.780831000
H	0.038734000	2.387421000	4.208039000
C	-1.818693000	-1.820668000	3.167063000
H	-1.112033000	-3.608186000	4.144783000
H	-2.285891000	-0.081463000	1.996973000
H	0.817626000	-3.891423000	2.579176000
H	-2.656324000	-1.674760000	3.844612000

C	4.055988000	2.188840000	-1.408882000
H	4.970991000	2.330616000	-1.995209000
H	4.068991000	2.904998000	-0.579164000
H	4.142724000	1.176177000	-0.975029000
C	2.801044000	2.366229000	-2.278702000
H	2.831404000	1.651648000	-3.112253000
H	2.823267000	3.367814000	-2.737599000
C	1.510365000	2.189861000	-1.487211000
H	1.372829000	2.975799000	-0.732066000
H	0.626273000	2.164588000	-2.131916000

62

A1,E(Coor3)

Pd	-1.886046000	0.393962000	-0.016679000
P	0.384738000	-0.227681000	-0.013289000
C	1.490963000	1.130561000	0.557061000
C	1.205671000	2.479093000	0.279180000
S	-0.271131000	2.989065000	-0.622722000
O	-1.398600000	2.503660000	0.286359000
O	-0.247579000	2.236227000	-1.892555000
O	-0.231533000	4.451046000	-0.698864000
C	2.056466000	3.488153000	0.726669000
C	3.192879000	3.172595000	1.466584000
C	2.628456000	0.832070000	1.316624000
C	0.974411000	-0.775537000	-1.655085000
C	0.057557000	-0.756036000	-2.711045000
H	-0.954808000	-0.420538000	-2.507802000
C	0.426359000	-1.120557000	-4.003148000
H	-0.303666000	-1.091106000	-4.806248000
C	1.741322000	-1.497870000	-4.252564000
H	2.049977000	-1.778595000	-5.256465000
C	2.683460000	-1.512391000	-3.225583000
H	3.705738000	-1.802230000	-3.442244000
C	2.309137000	-1.149780000	-1.927986000
O	3.167526000	-1.146816000	-0.879039000
C	4.535650000	-1.411812000	-1.117416000
H	4.690904000	-2.436383000	-1.479948000
H	5.033681000	-1.292394000	-0.153898000
H	4.961055000	-0.700590000	-1.836192000
C	0.684932000	-1.617156000	1.139067000
C	1.099870000	-2.880644000	0.713828000
H	1.333328000	-3.036562000	-0.334330000
C	1.214334000	-3.943768000	1.609797000
H	1.541435000	-4.917708000	1.257396000
C	0.907820000	-3.744092000	2.951735000
H	0.997534000	-4.562628000	3.661464000

C	0.473373000	-2.497782000	3.403195000
H	0.227431000	-2.360117000	4.450632000
C	0.349338000	-1.439241000	2.500028000
O	-0.090483000	-0.203665000	2.832776000
C	-0.437030000	0.069142000	4.176570000
H	-1.263390000	-0.569511000	4.514023000
H	-0.752677000	1.112860000	4.192262000
H	0.423946000	-0.062260000	4.844298000
C	3.474325000	1.842177000	1.767905000
C	-2.378339000	-1.602244000	-0.174174000
H	-1.504823000	-2.161300000	-0.527381000
H	-2.551722000	-1.903959000	0.869068000
H	3.851148000	3.965493000	1.812630000
H	2.853166000	-0.203002000	1.551876000
H	1.799701000	4.515546000	0.488928000
H	4.353486000	1.585758000	2.354007000
C	-3.926376000	1.264442000	-0.526907000
H	-4.367975000	0.632422000	-1.291119000
H	-3.606547000	2.258473000	-0.825705000
C	-3.938950000	0.910037000	0.794783000
H	-3.649742000	1.627810000	1.558552000
H	-4.398702000	-0.014597000	1.133985000
C	-3.572366000	-1.948640000	-1.054647000
H	-4.490362000	-1.480704000	-0.675880000
H	-3.421088000	-1.558554000	-2.070700000
C	-3.797403000	-3.463690000	-1.134370000
H	-4.662502000	-3.704293000	-1.764352000
H	-2.922088000	-3.970759000	-1.557543000
H	-3.977127000	-3.888449000	-0.139235000

62

A1,E(Coor4)

Pd	1.762769000	0.244725000	-0.861405000
P	-0.652116000	0.100994000	-0.152535000
C	-0.854010000	-0.845186000	1.416784000
C	-0.012014000	-1.933695000	1.706711000
S	1.295863000	-2.466203000	0.584417000
O	2.246347000	-1.258941000	0.553914000
O	0.654942000	-2.659058000	-0.729969000
O	1.944377000	-3.618476000	1.210046000
C	-0.148737000	-2.640436000	2.900167000
C	-1.116191000	-2.265223000	3.828624000
C	-1.808521000	-0.471999000	2.369590000
C	-1.820025000	-0.632663000	-1.360236000
C	-1.269937000	-1.147861000	-2.539323000
H	-0.192711000	-1.112412000	-2.664966000

C	-2.062203000	-1.747057000	-3.515983000
H	-1.606839000	-2.145132000	-4.417752000
C	-3.433006000	-1.849066000	-3.308087000
H	-4.068001000	-2.317668000	-4.055846000
C	-4.010805000	-1.366739000	-2.134717000
H	-5.080287000	-1.468214000	-1.986285000
C	-3.212216000	-0.766447000	-1.155913000
O	-3.705923000	-0.280626000	0.009516000
C	-5.078919000	-0.459248000	0.293789000
H	-5.713139000	0.072907000	-0.427305000
H	-5.234461000	-0.036876000	1.287894000
H	-5.350700000	-1.522183000	0.304799000
C	-1.294531000	1.781580000	0.204045000
C	-2.277804000	2.404563000	-0.568648000
H	-2.768721000	1.839747000	-1.354840000
C	-2.638268000	3.734984000	-0.351499000
H	-3.408872000	4.195025000	-0.963515000
C	-2.009053000	4.460440000	0.654815000
H	-2.285810000	5.495225000	0.840006000
C	-1.009077000	3.873814000	1.431164000
H	-0.518491000	4.453891000	2.205506000
C	-0.641690000	2.545687000	1.198948000
O	0.343562000	1.901767000	1.868870000
C	1.048719000	2.583774000	2.887715000
H	1.568413000	3.466524000	2.493387000
H	1.780866000	1.869910000	3.266373000
H	0.377631000	2.887127000	3.701383000
C	-1.941646000	-1.174602000	3.564894000
H	-1.218719000	-2.820202000	4.757674000
H	-2.452993000	0.377127000	2.167836000
H	0.518807000	-3.476651000	3.081712000
H	-2.693350000	-0.868404000	4.288469000
C	3.778555000	0.201826000	-1.272571000
H	3.925215000	-0.863129000	-1.487169000
H	3.998470000	0.790756000	-2.172404000
C	1.739140000	2.268000000	-1.630493000
H	2.750188000	2.654085000	-1.534520000
H	0.967964000	2.801037000	-1.080348000
C	1.400124000	1.400586000	-2.658002000
H	0.359542000	1.255287000	-2.935888000
H	2.136087000	1.082374000	-3.392159000
C	4.596147000	0.655410000	-0.077453000
H	4.405310000	1.717136000	0.136274000
H	4.284924000	0.088299000	0.806742000
C	6.101003000	0.454653000	-0.304134000

H	6.454182000	1.020026000	-1.175602000
H	6.677559000	0.786538000	0.567977000
H	6.331854000	-0.603071000	-0.475597000

62

A1,E(TS2)

Pd	-1.678518000	0.315040000	0.506511000
P	0.629119000	0.146675000	0.150203000
C	1.072501000	-0.892231000	-1.305861000
C	0.308016000	-2.018494000	-1.657856000
S	-1.160239000	-2.517082000	-0.735008000
O	-2.071160000	-1.294234000	-0.880576000
O	-0.720991000	-2.699403000	0.660274000
O	-1.718806000	-3.676987000	-1.432583000
C	0.652803000	-2.789128000	-2.766639000
C	1.753170000	-2.443932000	-3.546888000
C	2.165312000	-0.550045000	-2.110562000
C	1.538131000	-0.527824000	1.588996000
C	0.785275000	-0.918757000	2.700963000
H	-0.294229000	-0.814004000	2.653285000
C	1.383182000	-1.473434000	3.829697000
H	0.773569000	-1.775091000	4.676031000
C	2.761232000	-1.657771000	3.845844000
H	3.245973000	-2.095112000	4.715156000
C	3.538639000	-1.297256000	2.746594000
H	4.610753000	-1.457890000	2.774975000
C	2.935007000	-0.738853000	1.615460000
O	3.627759000	-0.362707000	0.512663000
C	5.011187000	-0.645672000	0.447732000
H	5.572154000	-0.095647000	1.214679000
H	5.336031000	-0.315652000	-0.540338000
H	5.204901000	-1.720363000	0.552968000
C	1.357144000	1.794097000	-0.189575000
C	2.249226000	2.424348000	0.680099000
H	2.600972000	1.889755000	1.556497000
C	2.689204000	3.727843000	0.447276000
H	3.386062000	4.196437000	1.136265000
C	2.231030000	4.416015000	-0.671329000
H	2.570490000	5.429854000	-0.867980000
C	1.324797000	3.820005000	-1.548668000
H	0.967635000	4.372119000	-2.411395000
C	0.878367000	2.518533000	-1.304743000
O	-0.022783000	1.867200000	-2.075660000
C	-0.556484000	2.513818000	-3.214492000
H	-1.096753000	3.428698000	-2.938898000
H	-1.251523000	1.800221000	-3.658187000

H	0.230533000	2.757752000	-3.939515000
C	2.505803000	-1.318031000	-3.221450000
H	2.015324000	-3.049111000	-4.411080000
H	2.754848000	0.325813000	-1.860151000
H	0.035215000	-3.649605000	-3.003559000
H	3.360423000	-1.033905000	-3.830709000
C	-3.940314000	0.175053000	0.845038000
H	-3.779182000	-0.531051000	0.024936000
H	-4.008740000	-0.380998000	1.783630000
C	-2.865391000	1.877647000	1.671642000
H	-3.421926000	1.627544000	2.570202000
H	-3.364017000	2.585911000	1.017276000
C	-1.444623000	1.943663000	1.751329000
H	-0.926323000	2.719158000	1.189676000
H	-0.955274000	1.656003000	2.681293000
C	-5.180592000	0.999513000	0.550814000
H	-5.409883000	1.696982000	1.367611000
H	-5.014811000	1.607095000	-0.349401000
C	-6.392744000	0.088737000	0.322617000
H	-6.600570000	-0.523099000	1.208252000
H	-7.288802000	0.682836000	0.106738000
H	-6.225444000	-0.591296000	-0.519876000

62

A1,E(P2)

Pd	-1.635382000	-0.014051000	0.085601000
P	0.597573000	0.200944000	0.151298000
C	1.370723000	-0.902072000	-1.105107000
C	0.838134000	-2.163166000	-1.430168000
S	-0.661484000	-2.834706000	-0.669674000
O	-1.725273000	-1.816490000	-1.076718000
O	-0.411477000	-2.809711000	0.783541000
O	-0.894968000	-4.136204000	-1.297635000
C	1.461330000	-2.961062000	-2.388009000
C	2.607117000	-2.516106000	-3.042323000
C	2.516292000	-0.463771000	-1.780982000
C	1.312942000	-0.212280000	1.780713000
C	0.444707000	-0.601357000	2.805373000
H	-0.618494000	-0.642482000	2.592075000
C	0.920866000	-0.970298000	4.060439000
H	0.226177000	-1.274955000	4.837073000
C	2.291427000	-0.967151000	4.295105000
H	2.681635000	-1.259086000	5.266855000
C	3.184141000	-0.602412000	3.288844000
H	4.249860000	-0.615235000	3.488816000
C	2.703844000	-0.229663000	2.029969000

O	3.508893000	0.140260000	1.004213000
C	4.910090000	0.038765000	1.166647000
H	5.275982000	0.722408000	1.943696000
H	5.341800000	0.322818000	0.205680000
H	5.210194000	-0.987776000	1.410240000
C	1.141387000	1.896781000	-0.255484000
C	1.790690000	2.717015000	0.670010000
H	2.053362000	2.313997000	1.642587000
C	2.098339000	4.043154000	0.368411000
H	2.606827000	4.662581000	1.101427000
C	1.749074000	4.561058000	-0.874897000
H	1.987405000	5.591835000	-1.124658000
C	1.081290000	3.772438000	-1.811350000
H	0.804828000	4.194548000	-2.771449000
C	0.764623000	2.446959000	-1.502033000
O	0.090379000	1.615147000	-2.326587000
C	-0.325803000	2.083148000	-3.595714000
H	-1.012749000	2.933689000	-3.501919000
H	-0.845055000	1.244847000	-4.061199000
H	0.533338000	2.371136000	-4.214668000
C	3.132011000	-1.262111000	-2.741522000
H	3.082400000	-3.146102000	-3.789926000
H	2.930496000	0.511713000	-1.548398000
H	1.019829000	-3.927336000	-2.610118000
H	4.020949000	-0.900276000	-3.252310000
C	-4.289885000	0.707870000	0.890960000
H	-3.917153000	-0.120108000	0.248261000
H	-4.237214000	0.326851000	1.920290000
C	-3.407323000	1.963895000	0.765815000
H	-3.823707000	2.754146000	1.412919000
H	-3.471163000	2.350517000	-0.261134000
C	-1.951783000	1.694346000	1.130739000
H	-1.286507000	2.515389000	0.850781000
H	-1.826358000	1.476926000	2.199475000
C	-5.748846000	0.949536000	0.500732000
H	-6.145226000	1.761037000	1.128488000
H	-5.788275000	1.316600000	-0.534371000
C	-6.626343000	-0.291657000	0.640858000
H	-6.632494000	-0.659123000	1.674152000
H	-7.663115000	-0.079881000	0.356174000
H	-6.264562000	-1.106392000	0.002203000

55

A2(P1)

Pd	-0.123711000	-1.789319000	-0.627245000
P	0.221662000	0.058629000	0.557033000

C	0.974828000	1.394075000	-0.444053000
C	1.893983000	1.104062000	-1.465769000
S	2.398122000	-0.578751000	-1.907552000
O	1.087263000	-1.249545000	-2.314524000
O	2.934938000	-1.156142000	-0.659441000
O	3.311074000	-0.446673000	-3.044011000
C	2.479228000	2.150395000	-2.174094000
C	2.160294000	3.472303000	-1.880365000
C	0.657490000	2.728984000	-0.168958000
C	1.390543000	-0.164536000	1.951888000
C	1.693140000	-1.453564000	2.401982000
H	1.250426000	-2.309267000	1.902283000
C	2.591128000	-1.639515000	3.450583000
H	2.826354000	-2.646521000	3.784479000
C	3.202595000	-0.541080000	4.051421000
H	3.909951000	-0.687760000	4.863617000
C	2.922654000	0.746386000	3.593708000
H	3.413548000	1.605543000	4.043408000
C	2.025931000	0.935914000	2.545219000
C	-1.313280000	0.770054000	1.326446000
C	-1.303193000	1.014552000	2.707660000
H	-0.399731000	0.815406000	3.272715000
C	-2.417476000	1.512238000	3.381734000
H	-2.359084000	1.689173000	4.452302000
C	-3.584529000	1.788362000	2.682305000
H	-4.455319000	2.193734000	3.190116000
C	-3.631284000	1.541712000	1.312428000
H	-4.524668000	1.768612000	0.740918000
C	-2.524871000	1.022364000	0.640573000
C	1.240229000	3.784279000	-0.875866000
C	0.861343000	5.212590000	-0.584994000
H	0.169133000	5.596215000	-1.345562000
H	0.366691000	5.309125000	0.386997000
H	1.739615000	5.867965000	-0.584972000
H	2.629381000	4.273856000	-2.447347000
H	-0.074964000	2.958719000	0.601439000
H	3.184820000	1.901894000	-2.960512000
C	-1.221460000	-4.452219000	-0.944641000
H	-1.122805000	-5.532307000	-1.102742000
H	-2.176144000	-4.128398000	-1.374129000
H	-0.413245000	-3.998635000	-1.546505000
C	-1.315874000	-2.603779000	0.802327000
H	-2.320437000	-2.253333000	0.539308000
H	-1.097493000	-2.327471000	1.838591000
C	-1.132550000	-4.095708000	0.548420000

H	-0.165669000	-4.433929000	0.944842000
H	-1.901389000	-4.663435000	1.097602000
H	1.834716000	1.940255000	2.177461000
S	-2.771902000	0.708419000	-1.120401000
O	-1.567519000	1.121742000	-1.824619000
O	-4.043574000	1.447426000	-1.494040000
O	-3.152033000	-0.755680000	-1.264235000
Li	-4.807271000	-0.213717000	-1.956680000

61

A2, E(Coor3)

Pd	-1.331767000	-1.035248000	-0.843355000
P	0.261775000	-0.045375000	0.565102000
C	1.944582000	0.059570000	-0.158682000
C	2.423445000	-0.955816000	-1.003133000
S	1.440859000	-2.406949000	-1.434041000
O	0.233671000	-1.817414000	-2.155623000
O	1.048741000	-3.006209000	-0.142758000
O	2.268266000	-3.212742000	-2.335097000
C	3.710481000	-0.867187000	-1.526233000
C	4.525247000	0.218466000	-1.220363000
C	2.777255000	1.144847000	0.134085000
C	0.505773000	-0.994753000	2.117304000
C	-0.540765000	-1.792556000	2.592789000
H	-1.461954000	-1.859804000	2.022039000
C	-0.390457000	-2.525904000	3.767358000
H	-1.207729000	-3.148771000	4.121532000
C	0.811094000	-2.478940000	4.472269000
H	0.932209000	-3.060259000	5.382802000
C	1.864287000	-1.698845000	3.996153000
H	2.810347000	-1.673152000	4.530839000
C	1.715275000	-0.960795000	2.823979000
C	-0.112538000	1.679043000	1.170203000
C	-0.087423000	1.911522000	2.554828000
H	0.162994000	1.094587000	3.221792000
C	-0.364215000	3.161501000	3.104034000
H	-0.328417000	3.290491000	4.182453000
C	-0.667880000	4.234590000	2.274787000
H	-0.865547000	5.219710000	2.688146000
C	-0.712766000	4.034250000	0.899398000
H	-0.921818000	4.864241000	0.232498000
C	-0.459015000	2.776002000	0.351136000
C	4.070930000	1.244319000	-0.387824000
C	4.934782000	2.439914000	-0.082596000
H	4.868274000	3.186130000	-0.885166000
H	4.629167000	2.931788000	0.846769000

H	5.989103000	2.158235000	0.013184000
H	5.528820000	0.269190000	-1.637808000
H	2.413298000	1.942254000	0.777182000
H	4.054137000	-1.666382000	-2.175330000
C	-2.554127000	-2.654626000	-1.855597000
H	-3.290992000	-3.011135000	-1.142738000
H	-1.739318000	-3.327550000	-2.106509000
C	-2.732039000	-1.490981000	-2.555476000
H	-2.069727000	-1.236387000	-3.379282000
H	-3.619832000	-0.877268000	-2.427073000
H	2.548248000	-0.371850000	2.450549000
S	-0.648811000	2.667091000	-1.434333000
O	0.156531000	1.546606000	-1.904031000
O	-0.273819000	4.030588000	-1.982525000
O	-2.138699000	2.562576000	-1.709222000
Li	-2.029377000	4.176429000	-2.656429000
C	-2.860841000	-0.199605000	0.265448000
H	-3.153385000	0.650828000	-0.363953000
H	-2.461825000	0.213516000	1.199437000
C	-4.055486000	-1.087360000	0.592037000
H	-4.548814000	-1.431809000	-0.326019000
H	-3.733866000	-1.993156000	1.125400000
C	-5.090841000	-0.348721000	1.449607000
H	-5.457132000	0.548695000	0.936282000
H	-5.955026000	-0.987512000	1.670032000
H	-4.657263000	-0.028991000	2.405012000

61

A2, E(Coor4)

Pd	1.761614000	-0.419469000	-0.705806000
P	-0.753581000	-0.086233000	-0.421812000
C	-1.358179000	-0.687832000	1.216406000
C	-0.730292000	-1.766835000	1.863333000
S	0.641415000	-2.665785000	1.119840000
O	1.767237000	-1.634496000	1.077011000
O	0.168908000	-3.004587000	-0.239111000
O	0.962411000	-3.770077000	2.025692000
C	-1.184006000	-2.196977000	3.107312000
C	-2.257394000	-1.562951000	3.724087000
C	-2.435187000	-0.066080000	1.858161000
C	-1.790441000	-0.958754000	-1.665512000
C	-1.698820000	-0.565463000	-3.010180000
H	-1.078830000	0.284201000	-3.289440000
C	-2.415063000	-1.237145000	-3.997876000
H	-2.336940000	-0.916029000	-5.033624000
C	-3.224928000	-2.320458000	-3.656582000

H	-3.779743000	-2.850679000	-4.426378000
C	-3.313885000	-2.722729000	-2.325639000
H	-3.935169000	-3.571842000	-2.052996000
C	-2.602371000	-2.048067000	-1.334239000
C	-1.479306000	1.638840000	-0.495015000
C	-2.748103000	1.819772000	-1.071039000
H	-3.273162000	0.956472000	-1.465163000
C	-3.361844000	3.068153000	-1.143111000
H	-4.347251000	3.154393000	-1.593392000
C	-2.723451000	4.189706000	-0.624441000
H	-3.198374000	5.166270000	-0.659400000
C	-1.468334000	4.046284000	-0.043327000
H	-0.967065000	4.899415000	0.401696000
C	-0.850715000	2.795318000	0.012097000
C	-2.899624000	-0.485551000	3.109007000
C	-4.045319000	0.223585000	3.782398000
H	-3.678811000	0.954616000	4.515020000
H	-4.663925000	0.766216000	3.060093000
H	-4.690406000	-0.479609000	4.320611000
H	-2.600427000	-1.912414000	4.695783000
H	-2.932441000	0.771966000	1.378319000
H	-0.680261000	-3.038188000	3.573040000
C	3.787414000	-0.685749000	-0.617176000
H	3.888842000	-1.765176000	-0.465024000
H	4.263395000	-0.390035000	-1.560140000
C	1.968225000	0.734952000	-2.513383000
H	2.917125000	1.261705000	-2.462863000
H	1.094586000	1.374087000	-2.600367000
C	1.914679000	-0.619262000	-2.833414000
H	0.985864000	-1.092329000	-3.143544000
H	2.818364000	-1.182113000	-3.050714000
H	-2.664588000	-2.382229000	-0.304369000
S	0.785428000	2.769470000	0.751704000
O	0.961607000	1.473205000	1.401096000
O	0.865999000	3.977726000	1.663287000
O	1.773400000	3.083625000	-0.359495000
Li	2.278800000	4.620725000	0.592140000
C	4.298586000	0.120924000	0.560346000
H	3.736718000	-0.152132000	1.460172000
H	4.123939000	1.191220000	0.384607000
C	5.795944000	-0.119518000	0.803808000
H	6.152534000	0.468347000	1.658650000
H	5.991611000	-1.176024000	1.020123000
H	6.395997000	0.159692000	-0.071506000

61

A2,E(TS2)

Pd	1.608248000	-0.351657000	-0.782415000
P	-0.684888000	-0.171787000	-0.463832000
C	-1.254513000	-0.596530000	1.229657000
C	-0.636736000	-1.638478000	1.940797000
S	0.720072000	-2.613727000	1.257672000
O	1.810235000	-1.575492000	0.996504000
O	0.197527000	-3.175418000	-0.001418000
O	1.124103000	-3.551269000	2.308082000
C	-1.089458000	-1.966953000	3.215559000
C	-2.147685000	-1.270720000	3.791061000
C	-2.313288000	0.094772000	1.827090000
C	-1.676748000	-1.288632000	-1.533971000
C	-1.100535000	-1.804145000	-2.699655000
H	-0.066393000	-1.569320000	-2.932772000
C	-1.826547000	-2.654785000	-3.530340000
H	-1.362601000	-3.056707000	-4.427307000
C	-3.132849000	-3.008800000	-3.198348000
H	-3.696034000	-3.680751000	-3.841011000
C	-3.708371000	-2.515884000	-2.027486000
H	-4.719213000	-2.805435000	-1.751717000
C	-2.984763000	-1.663099000	-1.197257000
C	-1.422689000	1.510678000	-0.801559000
C	-2.494694000	1.599042000	-1.703871000
H	-2.871921000	0.693046000	-2.164146000
C	-3.102415000	2.811674000	-2.023222000
H	-3.931959000	2.823979000	-2.725175000
C	-2.659544000	3.989191000	-1.433398000
H	-3.139250000	4.938607000	-1.654639000
C	-1.587783000	3.939665000	-0.548103000
H	-1.237322000	4.842234000	-0.058575000
C	-0.962253000	2.727852000	-0.249955000
C	-2.774936000	-0.225842000	3.107708000
C	-3.894877000	0.557017000	3.741624000
H	-4.524134000	1.043648000	2.988979000
H	-4.535531000	-0.085864000	4.355202000
H	-3.500266000	1.344752000	4.396778000
H	-2.488287000	-1.542132000	4.788226000
H	-2.788151000	0.913662000	1.292148000
H	-0.593210000	-2.776253000	3.741852000
C	3.850033000	-0.650485000	-0.774461000
H	4.627612000	-0.760623000	-1.530782000
H	3.554119000	-1.655575000	-0.455297000
C	1.643224000	0.847753000	-2.460651000
H	1.394278000	1.861356000	-2.154120000

H	1.009673000	0.428062000	-3.240794000
C	3.012910000	0.449640000	-2.415185000
H	3.347351000	-0.282153000	-3.145806000
H	3.750130000	1.205705000	-2.159611000
H	-3.433878000	-1.301301000	-0.276334000
S	0.480853000	2.857918000	0.818325000
O	0.603438000	1.623122000	1.581410000
O	0.292046000	4.123740000	1.632897000
O	1.654034000	3.186879000	-0.092502000
Li	1.852205000	4.807324000	0.828934000
C	4.304208000	0.227664000	0.375654000
H	4.514010000	1.244351000	0.014400000
H	3.510142000	0.311046000	1.124502000
C	5.561500000	-0.349484000	1.038867000
H	6.388213000	-0.444629000	0.323307000
H	5.898620000	0.296383000	1.858513000
H	5.358255000	-1.341251000	1.456906000

61

A2,E(P2)

Pd	-1.470753000	-0.360791000	-0.426526000
P	0.514611000	-0.172863000	0.560107000
C	1.898984000	-0.551185000	-0.577598000
C	1.766706000	-1.526503000	-1.579677000
S	0.248660000	-2.475230000	-1.851285000
O	-0.791079000	-1.404196000	-2.174648000
O	-0.030554000	-3.122847000	-0.554205000
O	0.506900000	-3.340946000	-3.003078000
C	2.854199000	-1.814632000	-2.399786000
C	4.062983000	-1.145424000	-2.236394000
C	3.118942000	0.118663000	-0.433306000
C	0.758608000	-1.333323000	1.958772000
C	-0.349178000	-1.951925000	2.546994000
H	-1.340473000	-1.757234000	2.150301000
C	-0.177905000	-2.845741000	3.601651000
H	-1.045598000	-3.327870000	4.043894000
C	1.101305000	-3.138400000	4.070240000
H	1.234684000	-3.843234000	4.886922000
C	2.212532000	-2.542072000	3.474546000
H	3.214131000	-2.783323000	3.820927000
C	2.044898000	-1.647462000	2.420480000
C	0.856984000	1.510423000	1.271129000
C	1.224054000	1.590054000	2.622668000
H	1.326772000	0.676185000	3.196713000
C	1.468562000	2.808025000	3.255550000
H	1.755661000	2.817117000	4.303565000

C	1.355824000	3.994266000	2.543071000
H	1.560709000	4.950215000	3.017062000
C	0.976288000	3.949926000	1.203940000
H	0.900267000	4.861581000	0.621388000
C	0.710230000	2.733743000	0.575077000
C	4.214932000	-0.164177000	-1.253270000
C	5.508426000	0.592345000	-1.102657000
H	5.584399000	1.390120000	-1.852905000
H	5.589719000	1.061663000	-0.116697000
H	6.374567000	-0.064873000	-1.238022000
H	4.899523000	-1.386287000	-2.889229000
H	3.218661000	0.894095000	0.322846000
H	2.727665000	-2.571303000	-3.167644000
C	-4.342790000	-0.043495000	-0.459289000
H	-4.363661000	0.977894000	-0.864605000
H	-3.639777000	-0.610558000	-1.106576000
C	-2.431273000	0.592764000	1.089441000
H	-2.401503000	1.648270000	0.795795000
H	-2.000411000	0.484706000	2.089775000
C	-3.831001000	-0.003245000	0.992443000
H	-3.841433000	-1.022981000	1.404225000
H	-4.528890000	0.582649000	1.612014000
H	2.917512000	-1.207900000	1.945457000
S	0.178315000	2.858360000	-1.145838000
O	0.838278000	1.802351000	-1.898297000
O	0.503090000	4.277575000	-1.574202000
O	-1.340441000	2.820797000	-1.147784000
Li	-1.339226000	4.548474000	-1.873220000
C	-5.715453000	-0.703707000	-0.636423000
H	-5.928149000	-0.787286000	-1.709986000
H	-5.670943000	-1.732534000	-0.253565000
C	-6.858613000	0.049362000	0.043681000
H	-7.819791000	-0.435338000	-0.161851000
H	-6.737587000	0.085640000	1.132057000
H	-6.923537000	1.082847000	-0.319613000

73

A3(P1)

Pd	-2.724094000	0.167517000	-0.265743000
P	-0.529765000	-0.311428000	-0.135550000
C	0.251263000	0.936373000	0.966529000
C	-0.157601000	2.283025000	0.974185000
S	-1.388068000	2.956079000	-0.168333000
O	-2.669259000	2.244179000	0.259624000
O	-0.950764000	2.535739000	-1.511799000
O	-1.454002000	4.394488000	0.103395000

C	0.387281000	3.161952000	1.905256000
C	1.325876000	2.718771000	2.835034000
C	1.197565000	0.514599000	1.904702000
C	0.282522000	-0.449906000	-1.782215000
C	-0.609758000	-0.633584000	-2.849817000
H	-1.675244000	-0.560865000	-2.655295000
C	-0.162991000	-0.877641000	-4.143437000
H	-0.881970000	-1.008591000	-4.947530000
C	1.203698000	-0.936844000	-4.393574000
H	1.575002000	-1.127927000	-5.397244000
C	2.099203000	-0.718829000	-3.352389000
H	3.167865000	-0.724134000	-3.548417000
C	1.672759000	-0.460512000	-2.041986000
C	-0.264072000	-1.927278000	0.679048000
C	0.352930000	-2.995848000	0.024650000
H	0.788026000	-2.837754000	-0.955306000
C	0.431625000	-4.256796000	0.617188000
H	0.915157000	-5.073425000	0.089057000
C	-0.115526000	-4.457760000	1.879415000
H	-0.059194000	-5.434996000	2.352503000
C	-0.751832000	-3.413781000	2.551083000
H	-1.183047000	-3.588386000	3.530864000
C	-0.839626000	-2.154613000	1.952670000
O	-1.465259000	-1.095408000	2.513037000
C	-2.074917000	-1.241973000	3.781217000
H	-2.863940000	-2.004401000	3.760016000
H	-2.513534000	-0.270099000	4.009961000
H	-1.336565000	-1.500386000	4.550921000
C	1.748418000	1.389013000	2.847821000
C	2.777151000	0.898531000	3.832000000
H	2.959276000	1.635967000	4.620302000
H	2.462215000	-0.036433000	4.310065000
H	3.731494000	0.699754000	3.329351000
H	1.729942000	3.419071000	3.563388000
H	1.519666000	-0.523156000	1.904201000
H	0.049243000	4.193525000	1.892464000
C	-5.499024000	-0.646032000	-0.643680000
H	-6.465191000	-0.558948000	-1.153750000
H	-5.685694000	-0.990716000	0.380080000
H	-5.109815000	0.386485000	-0.582458000
C	-3.207502000	-1.754873000	-0.696220000
H	-3.298228000	-2.246753000	0.282385000
H	-2.485335000	-2.312901000	-1.298969000
C	-4.555375000	-1.602180000	-1.391114000
H	-4.411031000	-1.241066000	-2.418406000

H	-5.042595000	-2.586508000	-1.481540000
C	2.751261000	-0.128365000	-1.064243000
C	3.051087000	1.228391000	-0.826842000
C	3.604414000	-1.108576000	-0.532395000
C	4.139847000	1.592823000	-0.025193000
C	4.686489000	-0.754384000	0.283271000
C	4.937307000	0.594209000	0.523089000
H	4.366643000	2.636123000	0.160772000
H	5.337868000	-1.508627000	0.709061000
H	5.786799000	0.872550000	1.142346000
O	3.315780000	-2.394013000	-0.878759000
O	2.233190000	2.103427000	-1.441649000
C	4.153895000	-3.420477000	-0.391912000
H	4.128504000	-3.479200000	0.704107000
H	3.762041000	-4.350019000	-0.809219000
H	5.191681000	-3.286086000	-0.724426000
C	2.437061000	3.492171000	-1.262048000
H	1.618579000	3.973593000	-1.796948000
H	2.385119000	3.769066000	-0.202506000
H	3.402127000	3.808690000	-1.680424000

79

A3(Coor3)

Pd	2.647935000	-0.448795000	-0.118023000
P	0.367001000	0.215723000	-0.003011000
C	-0.647675000	-1.062919000	0.847366000
C	-0.386968000	-2.433810000	0.679388000
S	0.879996000	-3.043961000	-0.445091000
O	2.167590000	-2.552006000	0.211308000
O	0.638844000	-2.367828000	-1.733694000
O	0.788797000	-4.507208000	-0.421408000
C	-1.094267000	-3.373360000	1.424310000
C	-2.056143000	-2.963783000	2.344281000
C	-1.618202000	-0.675849000	1.776291000
C	-0.320413000	0.717579000	-1.637895000
C	0.670380000	1.004743000	-2.589964000
H	1.707088000	0.803529000	-2.337313000
C	0.357347000	1.514930000	-3.844802000
H	1.150590000	1.720818000	-4.558431000
C	-0.974185000	1.742720000	-4.175133000
H	-1.242669000	2.141632000	-5.150144000
C	-1.969071000	1.427353000	-3.256403000
H	-3.013457000	1.565814000	-3.522294000
C	-1.677235000	0.903866000	-1.988513000
C	0.196312000	1.698826000	1.060513000
C	-0.319010000	2.911378000	0.599564000

H	-0.734962000	2.965728000	-0.400191000
C	-0.321211000	4.048871000	1.409544000
H	-0.725713000	4.982785000	1.029946000
C	0.199477000	3.977062000	2.696283000
H	0.202117000	4.855459000	3.336967000
C	0.732472000	2.781591000	3.180127000
H	1.142334000	2.742837000	4.183744000
C	0.743201000	1.648579000	2.364164000
O	1.262476000	0.453972000	2.733749000
C	1.811641000	0.311302000	4.028951000
H	2.661216000	0.989344000	4.181250000
H	2.154282000	-0.722175000	4.091196000
H	1.055724000	0.492750000	4.803628000
C	-2.334637000	-1.608424000	2.533204000
C	3.222420000	1.530259000	-0.222552000
H	2.345927000	2.154794000	-0.420981000
H	3.533496000	1.735561000	0.812732000
C	-3.391416000	-1.154392000	3.505087000
H	-3.090931000	-0.238833000	4.026640000
H	-4.330913000	-0.936033000	2.982270000
H	-3.599158000	-1.921860000	4.258045000
H	-2.592508000	-3.709941000	2.927324000
H	-1.826174000	0.381367000	1.915715000
H	-0.863573000	-4.423911000	1.276786000
C	4.568120000	-1.347110000	-0.921535000
H	4.944488000	-0.682083000	-1.692762000
H	4.176015000	-2.308364000	-1.240834000
C	4.753379000	-1.084210000	0.409773000
H	4.527931000	-1.843475000	1.154506000
H	5.291328000	-0.202183000	0.747314000
C	-2.860067000	0.509377000	-1.167238000
C	-3.278912000	-0.836100000	-1.186322000
C	-3.681612000	1.460050000	-0.540799000
C	-4.461147000	-1.228854000	-0.546735000
C	-4.857440000	1.074843000	0.115195000
C	-5.229438000	-0.266926000	0.099952000
H	-4.780543000	-2.264425000	-0.559515000
H	-5.486116000	1.803658000	0.613211000
H	-6.150563000	-0.566633000	0.594235000
O	-3.264187000	2.754093000	-0.633599000
O	-2.473254000	-1.669232000	-1.872402000
C	-4.065939000	3.757306000	-0.047728000
H	-4.141503000	3.632740000	1.040542000
H	-3.568115000	4.704145000	-0.265913000
H	-5.074589000	3.773209000	-0.481497000

C	-2.794603000	-3.045081000	-1.943271000
H	-1.965975000	-3.506049000	-2.480539000
H	-2.865287000	-3.490049000	-0.943664000
H	-3.735682000	-3.205396000	-2.486777000
C	4.328976000	1.897844000	-1.203053000
H	5.259195000	1.365450000	-0.965582000
H	4.052712000	1.596616000	-2.223263000
C	4.616528000	3.404346000	-1.194073000
H	4.921019000	3.741590000	-0.195732000
H	5.420531000	3.659839000	-1.895180000
H	3.726367000	3.977500000	-1.479271000

79

A3(Coor4)

Pd	-2.659365000	-0.534801000	-0.540257000
P	-0.149610000	-0.365925000	-0.215274000
C	0.269515000	1.110340000	0.804126000
C	-0.484582000	2.290444000	0.673099000
S	-1.754232000	2.478949000	-0.588313000
O	-2.868051000	1.526205000	-0.123973000
O	-1.144419000	2.009701000	-1.844627000
O	-2.212238000	3.867773000	-0.519412000
C	-0.270467000	3.359525000	1.537295000
C	0.687554000	3.265189000	2.545004000
C	1.227564000	1.042597000	1.817426000
C	0.854070000	-0.461945000	-1.763898000
C	0.075200000	-0.756290000	-2.894769000
H	-1.005709000	-0.761718000	-2.794781000
C	0.644062000	-0.997263000	-4.140876000
H	0.007006000	-1.214373000	-4.994137000
C	2.026427000	-0.941242000	-4.280417000
H	2.494468000	-1.131445000	-5.243068000
C	2.809919000	-0.602565000	-3.182512000
H	3.886664000	-0.510730000	-3.295841000
C	2.254686000	-0.340476000	-1.921757000
C	0.401610000	-1.792177000	0.803578000
C	1.241698000	-2.795552000	0.316544000
H	1.700816000	-2.674985000	-0.657926000
C	1.518153000	-3.940157000	1.067680000
H	2.175084000	-4.705440000	0.664325000
C	0.950937000	-4.090129000	2.327445000
H	1.162888000	-4.973240000	2.925061000
C	0.092926000	-3.113657000	2.836420000
H	-0.352097000	-3.246751000	3.816799000
C	-0.195747000	-1.979168000	2.074409000
O	-1.057100000	-1.007542000	2.463117000

C	-1.682064000	-1.104313000	3.727642000
H	-2.301170000	-2.007891000	3.802554000
H	-2.316869000	-0.221516000	3.810472000
H	-0.942515000	-1.101476000	4.538586000
C	1.451820000	2.106847000	2.699650000
C	2.496952000	1.991681000	3.777669000
H	2.518354000	2.883590000	4.412068000
H	2.311244000	1.124632000	4.423319000
H	3.494225000	1.860637000	3.341089000
H	0.838750000	4.104251000	3.221224000
H	1.817032000	0.135880000	1.927885000
H	-0.868353000	4.256277000	1.407676000
C	-4.700105000	-0.431553000	-0.805067000
H	-4.766175000	0.374451000	-1.545123000
H	-5.073690000	-1.365046000	-1.245632000
C	-2.953785000	-2.625248000	-0.052479000
H	-3.986551000	-2.729213000	0.268265000
H	-2.195299000	-2.885385000	0.680713000
C	-2.625814000	-2.533027000	-1.395838000
H	-1.611638000	-2.739292000	-1.726272000
H	-3.392916000	-2.555402000	-2.166145000
C	3.208083000	0.149236000	-0.883079000
C	3.288297000	1.534992000	-0.639417000
C	4.167225000	-0.690874000	-0.292475000
C	4.253964000	2.057090000	0.230361000
C	5.125325000	-0.180531000	0.592396000
C	5.153374000	1.188949000	0.840038000
H	4.310265000	3.122350000	0.421692000
H	5.851588000	-0.829579000	1.067569000
H	5.906455000	1.590046000	1.514231000
O	4.107992000	-2.002325000	-0.654888000
O	2.398350000	2.285025000	-1.320594000
C	5.063811000	-2.889119000	-0.113242000
H	4.969205000	-2.969997000	0.977530000
H	4.858524000	-3.861966000	-0.564447000
H	6.087537000	-2.584452000	-0.367733000
C	2.381819000	3.685597000	-1.122652000
H	1.560682000	4.055166000	-1.737681000
H	2.188018000	3.936938000	-0.073397000
H	3.325396000	4.145053000	-1.446973000
C	-5.422012000	-0.076032000	0.480889000
H	-4.961410000	0.817464000	0.916147000
H	-5.313933000	-0.885638000	1.217124000
C	-6.915604000	0.187446000	0.244875000
H	-7.422784000	0.441848000	1.183624000

H	-7.059452000	1.022792000	-0.450189000
H	-7.416148000	-0.691677000	-0.180064000

79

A3,E(TS2)

Pd	2.502874000	0.440880000	-0.608450000
P	0.183215000	0.407114000	-0.229295000
C	-0.284744000	-0.970145000	0.898968000
C	0.370394000	-2.212654000	0.826165000
S	1.593161000	-2.593228000	-0.442470000
O	2.742423000	-1.641825000	-0.103970000
O	0.961576000	-2.249986000	-1.728180000
O	1.988926000	-3.988595000	-0.229859000
C	0.095556000	-3.199689000	1.767918000
C	-0.820828000	-2.962697000	2.790360000
C	-1.202117000	-0.756624000	1.930746000
C	-0.817456000	0.425451000	-1.779907000
C	-0.058678000	0.708400000	-2.926491000
H	1.022087000	0.757598000	-2.832766000
C	-0.647223000	0.889598000	-4.173203000
H	-0.025920000	1.099869000	-5.039685000
C	-2.028188000	0.783171000	-4.296453000
H	-2.511205000	0.924431000	-5.260120000
C	-2.789918000	0.459135000	-3.179114000
H	-3.864424000	0.330708000	-3.276986000
C	-2.215529000	0.259108000	-1.915557000
C	-0.302820000	1.930421000	0.673261000
C	-1.146462000	2.896153000	0.122751000
H	-1.635231000	2.694766000	-0.823189000
C	-1.383797000	4.108315000	0.774768000
H	-2.043648000	4.844247000	0.324359000
C	-0.770721000	4.363175000	1.995383000
H	-0.949404000	5.301115000	2.515393000
C	0.090214000	3.423280000	2.564409000
H	0.570181000	3.640002000	3.512817000
C	0.336137000	2.217952000	1.903316000
O	1.186585000	1.267380000	2.358807000
C	1.841899000	1.464698000	3.595750000
H	2.488063000	2.351778000	3.571548000
H	2.452770000	0.574695000	3.750929000
H	1.121050000	1.560718000	4.417831000
C	-1.485848000	-1.738824000	2.886315000
C	-2.496835000	-1.477879000	3.971493000
H	-2.348281000	-0.493977000	4.431389000
H	-3.515668000	-1.493525000	3.565044000
H	-2.438720000	-2.233215000	4.761911000

H	-1.016771000	-3.740580000	3.525794000
H	-1.713766000	0.200023000	1.995843000
H	0.621135000	-4.146219000	1.687887000
C	2.520625000	2.465257000	-0.997652000
H	2.289153000	2.999853000	-0.077348000
H	1.853973000	2.693827000	-1.827521000
C	3.882621000	2.154080000	-1.283538000
H	4.648389000	2.541696000	-0.616620000
C	-3.149029000	-0.215659000	-0.851268000
C	-3.222770000	-1.598497000	-0.588317000
C	-4.097430000	0.631612000	-0.255367000
C	-4.174969000	-2.110935000	0.301531000
C	-5.042384000	0.131145000	0.649198000
C	-5.066059000	-1.235696000	0.913043000
H	-4.226508000	-3.173833000	0.506999000
H	-5.762662000	0.785252000	1.126656000
H	-5.809671000	-1.629020000	1.602235000
O	-4.042108000	1.939176000	-0.633078000
O	-2.339416000	-2.352020000	-1.271510000
C	-4.986473000	2.833293000	-0.084215000
H	-4.875015000	2.923183000	1.004291000
H	-4.785287000	3.801927000	-0.546037000
H	-6.015050000	2.529772000	-0.320130000
C	-2.313801000	-3.751190000	-1.065304000
H	-1.485368000	-4.115742000	-1.672930000
H	-2.123154000	-3.995384000	-0.013707000
H	-3.253636000	-4.218197000	-1.389921000
C	4.751279000	0.237546000	-0.832662000
H	5.503314000	0.676216000	-1.489084000
H	4.463335000	-0.728916000	-1.259667000
H	4.178277000	2.109319000	-2.328529000
C	5.249329000	0.127216000	0.595432000
H	5.397791000	1.128353000	1.025504000
H	4.501814000	-0.383222000	1.211155000
C	6.568763000	-0.652531000	0.661918000
H	7.352238000	-0.167093000	0.066643000
H	6.927443000	-0.723314000	1.695686000
H	6.437075000	-1.671581000	0.282233000

79

A3,E(P2)

Pd	2.404604000	-0.139644000	-0.101941000
P	0.200044000	0.321610000	-0.084523000
C	-0.626883000	-0.928749000	0.980658000
C	-0.212899000	-2.273664000	1.002731000
S	1.077884000	-2.934617000	-0.078638000

O	2.331376000	-2.220927000	0.421216000
O	0.710889000	-2.507761000	-1.441293000
O	1.134942000	-4.374719000	0.187170000
C	-0.797060000	-3.158442000	1.903887000
C	-1.778999000	-2.721868000	2.791029000
C	-1.615295000	-0.512993000	1.877046000
C	-0.530289000	0.449196000	-1.770303000
C	0.412890000	0.640428000	-2.791753000
H	1.467674000	0.578381000	-2.543175000
C	0.029853000	0.876399000	-4.107038000
H	0.787284000	1.013952000	-4.873891000
C	-1.322943000	0.918437000	-4.426757000
H	-1.644881000	1.102892000	-5.448559000
C	-2.267574000	0.691618000	-3.431967000
H	-3.324935000	0.683637000	-3.681860000
C	-1.905343000	0.441746000	-2.100637000
C	-0.115255000	1.938221000	0.711222000
C	-0.704866000	3.001722000	0.024156000
H	-1.087638000	2.839222000	-0.976671000
C	-0.819464000	4.264069000	0.607951000
H	-1.280630000	5.076469000	0.053939000
C	-0.335758000	4.471711000	1.894676000
H	-0.420624000	5.449809000	2.361714000
C	0.273232000	3.433320000	2.599529000
H	0.655098000	3.613516000	3.598700000
C	0.397273000	2.172929000	2.010191000
O	1.001631000	1.119382000	2.603842000
C	1.549205000	1.273868000	3.899036000
H	2.331235000	2.043587000	3.913474000
H	1.985239000	0.306395000	4.150294000
H	0.772997000	1.526815000	4.632462000
C	-2.205286000	-1.393086000	2.790648000
C	-3.279870000	-0.910578000	3.728779000
H	-3.480019000	-1.642993000	4.517425000
H	-2.999874000	0.034654000	4.208163000
H	-4.217433000	-0.733841000	3.187569000
H	-2.214174000	-3.426504000	3.496923000
H	-1.938652000	0.524329000	1.866377000
H	-0.455203000	-4.188839000	1.902497000
C	5.178031000	0.678415000	-0.314341000
H	5.283922000	1.069311000	0.707762000
H	4.712270000	-0.325516000	-0.207072000
C	2.895495000	1.784392000	-0.511989000
H	2.929687000	2.282539000	0.466686000
H	2.205085000	2.334253000	-1.157743000

C	4.278795000	1.624335000	-1.129588000
H	4.191225000	1.242831000	-2.156903000
H	4.777292000	2.605045000	-1.212003000
C	-3.029098000	0.099860000	-1.178733000
C	-3.330374000	-1.259261000	-0.957020000
C	-3.916973000	1.072781000	-0.692492000
C	-4.456852000	-1.632276000	-0.213372000
C	-5.037646000	0.710116000	0.065202000
C	-5.290096000	-0.640390000	0.292080000
H	-4.684626000	-2.677363000	-0.039038000
H	-5.716712000	1.459216000	0.455397000
H	-6.168552000	-0.925700000	0.866078000
O	-3.621639000	2.360226000	-1.024852000
O	-2.475600000	-2.128454000	-1.528758000
C	-4.491033000	3.380412000	-0.582053000
H	-4.525211000	3.437469000	0.513844000
H	-4.082878000	4.313033000	-0.976152000
H	-5.508584000	3.240098000	-0.970150000
C	-2.677724000	-3.518175000	-1.355671000
H	-1.830608000	-3.995232000	-1.848335000
H	-2.676187000	-3.791147000	-0.293824000
H	-3.618694000	-3.843257000	-1.819961000
C	6.560667000	0.465528000	-0.932277000
H	7.041459000	1.447304000	-1.053397000
H	6.442460000	0.055514000	-1.944745000
C	7.457538000	-0.453391000	-0.106844000
H	7.008768000	-1.447759000	0.004865000
H	8.437943000	-0.581829000	-0.579149000
H	7.622675000	-0.048694000	0.899263000

61

A1,12(Coor3)

Pd	-1.391744000	0.543293000	-0.274795000
P	0.857286000	-0.007351000	0.134155000
C	1.483563000	-1.375631000	-0.928710000
C	0.653335000	-2.434653000	-1.333878000
S	-1.087009000	-2.512349000	-0.873421000
O	-1.643229000	-1.236288000	-1.507160000
O	-1.110545000	-2.436486000	0.598803000
O	-1.637811000	-3.709588000	-1.509825000
C	1.151414000	-3.456596000	-2.139287000
C	2.477968000	-3.433639000	-2.561616000
C	2.809980000	-1.358186000	-1.376345000
C	1.147808000	-0.468351000	1.878181000
C	0.041920000	-0.502366000	2.734616000
H	-0.935906000	-0.257468000	2.332347000

C	0.167416000	-0.881533000	4.068270000
H	-0.707901000	-0.905594000	4.709949000
C	1.416357000	-1.250247000	4.555802000
H	1.531617000	-1.555629000	5.592765000
C	2.533789000	-1.246473000	3.722882000
H	3.497254000	-1.547188000	4.119409000
C	2.406542000	-0.863276000	2.384377000
O	3.447706000	-0.834060000	1.516438000
C	4.703594000	-1.324637000	1.942553000
H	5.123220000	-0.711188000	2.750496000
H	5.356948000	-1.265926000	1.070579000
H	4.633099000	-2.368043000	2.273625000
C	1.949105000	1.413999000	-0.235742000
C	2.694714000	2.078476000	0.740111000
H	2.688302000	1.703593000	1.758359000
C	3.443797000	3.213480000	0.428435000
H	4.019758000	3.712810000	1.202145000
C	3.449169000	3.693418000	-0.877332000
H	4.034358000	4.572575000	-1.134788000
C	2.699266000	3.061932000	-1.869576000
H	2.704195000	3.454468000	-2.880744000
C	1.939290000	1.933846000	-1.549667000
O	1.153749000	1.270915000	-2.429473000
C	1.109135000	1.697302000	-3.777743000
H	0.721539000	2.720795000	-3.860780000
H	0.430222000	1.007998000	-4.280801000
H	2.100206000	1.641392000	-4.245391000
C	3.305513000	-2.378517000	-2.184588000
C	-1.093234000	2.256817000	0.844715000
H	-1.835572000	2.214428000	1.653035000
H	-0.107193000	2.232551000	1.318586000
H	2.860390000	-4.234123000	-3.189852000
H	3.459608000	-0.539621000	-1.084974000
H	0.475060000	-4.254448000	-2.429271000
H	4.340067000	-2.346068000	-2.517377000
C	-3.633084000	1.056963000	-0.077822000
H	-3.675075000	1.979614000	0.493088000
C	-3.293995000	1.066834000	-1.400571000
H	-3.037959000	1.999050000	-1.895711000
H	-3.406864000	0.178001000	-2.014159000
C	-4.340430000	-0.082025000	0.603401000
F	-4.352727000	-1.206462000	-0.109626000
F	-5.625596000	0.281024000	0.827011000
F	-3.797838000	-0.341432000	1.808577000
C	-1.230116000	3.528493000	0.019803000

H	-2.231043000	3.598243000	-0.429466000
H	-0.517497000	3.506508000	-0.814955000
C	-0.979431000	4.789234000	0.856992000
H	0.033212000	4.784118000	1.276330000
H	-1.088090000	5.696620000	0.250495000
H	-1.686714000	4.856781000	1.692590000

65

A1,12(Coor4)

Pd	-1.617603000	0.155884000	-0.027308000
P	0.876725000	-0.137261000	0.112610000
C	1.804803000	1.335310000	-0.494762000
C	1.327804000	2.088334000	-1.580662000
S	-0.230652000	1.691218000	-2.383847000
O	-1.228783000	1.850817000	-1.220694000
O	-0.136176000	0.287403000	-2.815699000
O	-0.462562000	2.717935000	-3.398973000
C	2.029285000	3.199586000	-2.043234000
C	3.213743000	3.584345000	-1.420675000
C	2.985179000	1.749655000	0.132333000
C	1.566812000	-1.585195000	-0.769362000
C	0.672527000	-2.359575000	-1.515414000
H	-0.370498000	-2.067321000	-1.541113000
C	1.099659000	-3.463573000	-2.249435000
H	0.383260000	-4.044385000	-2.822095000
C	2.449854000	-3.793707000	-2.254322000
H	2.803655000	-4.648118000	-2.825911000
C	3.370578000	-3.030069000	-1.538083000
H	4.421001000	-3.298415000	-1.563683000
C	2.939378000	-1.923584000	-0.800205000
O	3.778126000	-1.137740000	-0.081176000
C	5.168662000	-1.382441000	-0.152654000
H	5.427033000	-2.366342000	0.260171000
H	5.638984000	-0.605084000	0.451761000
H	5.534891000	-1.311110000	-1.184333000
C	1.384879000	-0.321892000	1.865269000
C	1.936128000	-1.497744000	2.379014000
H	2.167150000	-2.313912000	1.701953000
C	2.192613000	-1.643033000	3.743029000
H	2.625135000	-2.566054000	4.118252000
C	1.895114000	-0.598115000	4.611444000
H	2.096275000	-0.695303000	5.675254000
C	1.326951000	0.582431000	4.131431000
H	1.089868000	1.385271000	4.821309000
C	1.059330000	0.716804000	2.766653000
O	0.474504000	1.804071000	2.210173000

C	0.156864000	2.913405000	3.028705000
H	-0.574893000	2.645067000	3.801443000
H	-0.276867000	3.657487000	2.360000000
H	1.055729000	3.327208000	3.503006000
C	3.685947000	2.862653000	-0.326448000
H	3.759827000	4.451113000	-1.784048000
H	3.358355000	1.190700000	0.983912000
H	1.621441000	3.752995000	-2.883081000
H	4.603536000	3.163465000	0.173310000
C	-3.543609000	1.013466000	-0.180103000
H	-4.361739000	0.295147000	-0.215600000
H	-3.420657000	1.486695000	-1.155812000
C	-2.883034000	-1.386006000	0.883229000
C	-1.599316000	-1.462268000	1.405928000
H	-0.912598000	-2.232488000	1.066602000
H	-1.369201000	-0.999926000	2.361387000
H	-3.665694000	-0.901081000	1.457046000
C	-3.407025000	-2.430767000	-0.061319000
F	-4.025899000	-3.402320000	0.647783000
F	-2.433135000	-3.023508000	-0.774909000
F	-4.312453000	-1.949127000	-0.927640000
C	-3.678985000	2.017676000	0.944150000
H	-3.702124000	1.510807000	1.920684000
H	-2.807108000	2.682804000	0.949740000
C	-4.953569000	2.862193000	0.790578000
H	-5.850989000	2.232090000	0.798741000
H	-5.043550000	3.588457000	1.607515000
H	-4.941855000	3.416349000	-0.154503000

65

65

A1,12(TS2)

P	-0.944418000	-0.158451000	-0.085515000
C	-1.861260000	1.385470000	0.324202000
C	-1.297964000	2.369327000	1.155963000
S	0.344083000	2.196520000	1.885496000
O	1.249836000	2.115294000	0.650942000
O	0.322159000	0.921743000	2.621984000
O	0.598535000	3.424714000	2.640037000
C	-1.999363000	3.539118000	1.441601000
C	-3.262468000	3.751764000	0.895408000
C	-3.125221000	1.621578000	-0.228365000
C	-1.377902000	-1.416094000	1.168019000
C	-0.333263000	-2.010527000	1.883047000
H	0.684098000	-1.695498000	1.677775000
C	-0.577018000	-2.957960000	2.874105000

H	0.252304000	-3.400045000	3.417484000
C	-1.889283000	-3.308137000	3.171199000
H	-2.098936000	-4.040449000	3.946852000
C	-2.953683000	-2.720044000	2.489619000
H	-3.970408000	-3.000228000	2.742210000
C	-2.706298000	-1.772313000	1.492155000
O	-3.688204000	-1.163064000	0.783022000
C	-5.034962000	-1.405334000	1.138334000
H	-5.312970000	-2.454685000	0.974338000
H	-5.633924000	-0.768793000	0.485067000
H	-5.227144000	-1.134837000	2.183965000
C	-1.651259000	-0.747581000	-1.670212000
C	-2.196918000	-2.023087000	-1.827478000
H	-2.287711000	-2.670738000	-0.961461000
C	-2.620809000	-2.480515000	-3.075613000
H	-3.044879000	-3.475723000	-3.173226000
C	-2.498954000	-1.652221000	-4.186260000
H	-2.831441000	-1.993454000	-5.163363000
C	-1.942183000	-0.378827000	-4.063821000
H	-1.844614000	0.252535000	-4.940481000
C	-1.507741000	0.069900000	-2.814074000
O	-0.924847000	1.272815000	-2.599553000
C	-0.765322000	2.167458000	-3.683399000
H	-0.123910000	1.739955000	-4.464897000
H	-0.287584000	3.053962000	-3.264866000
H	-1.734718000	2.444491000	-4.116758000
C	-3.822874000	2.793397000	0.054222000
H	-3.803011000	4.667295000	1.121993000
H	-3.565669000	0.876954000	-0.883426000
H	-1.529112000	4.272397000	2.089023000
H	-4.804820000	2.954159000	-0.384114000
C	3.459994000	0.910512000	-0.922087000
C	2.986938000	-1.239297000	-0.976589000
C	1.637838000	-1.544730000	-1.357011000
H	3.730927000	-1.157326000	-1.762344000
H	1.369717000	-1.423067000	-2.405860000
C	4.870759000	0.826031000	-0.376683000
H	4.840265000	0.667731000	0.706052000
H	5.428622000	-0.011543000	-0.809514000
C	5.619233000	2.133139000	-0.670402000
H	5.121972000	2.988960000	-0.200847000
H	6.642466000	2.082002000	-0.280595000
H	5.680066000	2.326917000	-1.748153000
H	3.418006000	0.965573000	-2.012574000
H	2.968269000	1.783881000	-0.481697000

C	3.548182000	-1.960510000	0.244165000
F	4.866657000	-2.192548000	0.096031000
F	2.961026000	-3.164019000	0.416264000
F	3.382019000	-1.288782000	1.396724000
H	1.129479000	-2.366855000	-0.859621000
Pd	1.346940000	0.201781000	-0.329328000

65

A1,12(P2)

Pd	-0.999419000	-0.943491000	-0.026966000
P	0.890421000	0.244219000	0.184635000
C	2.027619000	-0.057723000	-1.231179000
C	2.116694000	-1.307684000	-1.869281000
S	1.107613000	-2.729184000	-1.390336000
O	-0.313645000	-2.204236000	-1.598929000
O	1.397996000	-2.958297000	0.036627000
O	1.426065000	-3.800589000	-2.334307000
C	3.000168000	-1.492649000	-2.930873000
C	3.795868000	-0.441014000	-3.377989000
C	2.823654000	0.993947000	-1.701561000
C	1.799925000	-0.150153000	1.719356000
C	1.290062000	-1.151917000	2.551852000
H	0.375981000	-1.655214000	2.254977000
C	1.952719000	-1.538299000	3.713541000
H	1.538690000	-2.323300000	4.338659000
C	3.156098000	-0.925848000	4.045418000
H	3.690081000	-1.218867000	4.945826000
C	3.701849000	0.060008000	3.225209000
H	4.647223000	0.517371000	3.495280000
C	3.035318000	0.446945000	2.058962000
O	3.505753000	1.394563000	1.211586000
C	4.785589000	1.947618000	1.450174000
H	4.811544000	2.511182000	2.391766000
H	4.974665000	2.628490000	0.618796000
H	5.559267000	1.170279000	1.466307000
C	0.562002000	2.041749000	0.195420000
C	0.775615000	2.834491000	1.325367000
H	1.223399000	2.389413000	2.207949000
C	0.417631000	4.182274000	1.339723000
H	0.596121000	4.781926000	2.227463000
C	-0.164503000	4.747710000	0.209416000
H	-0.442077000	5.798708000	0.204870000
C	-0.408624000	3.975971000	-0.926350000
H	-0.872970000	4.428742000	-1.795746000
C	-0.060800000	2.622079000	-0.933212000
O	-0.289945000	1.784430000	-1.967720000

C	-0.887369000	2.285125000	-3.149314000
H	-1.890727000	2.682394000	-2.951613000
H	-0.960022000	1.431895000	-3.824317000
H	-0.266496000	3.064612000	-3.608585000
C	3.702579000	0.805879000	-2.765256000
H	4.480085000	-0.595319000	-4.208439000
H	2.754529000	1.967482000	-1.227864000
H	3.036588000	-2.471441000	-3.398446000
H	4.311702000	1.636625000	-3.112798000
C	-3.828568000	0.920899000	-0.058387000
H	-3.166114000	1.795839000	-0.067752000
H	-3.603202000	0.367792000	-0.980527000
C	-3.429795000	0.073110000	1.165985000
C	-1.921049000	0.117224000	1.455029000
H	-1.687924000	-0.388131000	2.399891000
H	-1.600747000	1.159224000	1.531091000
H	-3.967081000	0.455820000	2.048906000
C	-3.861354000	-1.373259000	1.031573000
F	-3.622456000	-2.082655000	2.144035000
F	-3.142035000	-1.990313000	0.021306000
F	-5.151841000	-1.543473000	0.715558000
C	-5.280759000	1.406367000	-0.081324000
H	-5.964801000	0.551468000	-0.090760000
H	-5.488201000	1.958544000	0.846722000
C	-5.570576000	2.304724000	-1.281845000
H	-5.403265000	1.771094000	-2.225581000
H	-6.609818000	2.651749000	-1.277677000
H	-4.924022000	3.191422000	-1.280994000

65

A1,21(Coor3)

Pd	1.416765000	-0.237434000	-0.448674000
P	-0.866756000	0.183732000	0.015709000
C	-1.587267000	-1.174251000	1.034777000
C	-1.254675000	-2.523748000	0.818768000
S	-0.058665000	-3.047876000	-0.426637000
O	1.224409000	-2.363910000	0.042789000
O	-0.520934000	-2.485012000	-1.708855000
O	0.043313000	-4.504607000	-0.321984000
C	-1.827487000	-3.525681000	1.600231000
C	-2.726278000	-3.201527000	2.612929000
C	-2.482814000	-0.865286000	2.065398000
C	-1.943818000	0.356935000	-1.451592000
C	-1.364341000	0.194389000	-2.713848000
H	-0.302478000	-0.019654000	-2.769918000
C	-2.123989000	0.263191000	-3.878113000

H	-1.650069000	0.128641000	-4.845542000
C	-3.494187000	0.480668000	-3.782905000
H	-4.104722000	0.531096000	-4.680965000
C	-4.106119000	0.623989000	-2.539194000
H	-5.177699000	0.781234000	-2.485442000
C	-3.340534000	0.556350000	-1.370891000
O	-3.866788000	0.683428000	-0.128308000
C	-5.270955000	0.774901000	0.011347000
H	-5.662979000	1.691667000	-0.447993000
H	-5.461243000	0.801485000	1.085411000
H	-5.773235000	-0.097257000	-0.424965000
C	-1.065995000	1.704155000	1.009601000
C	-1.758847000	2.828889000	0.557589000
H	-2.282139000	2.783476000	-0.391700000
C	-1.782579000	4.008209000	1.301948000
H	-2.330531000	4.870908000	0.934343000
C	-1.101513000	4.067857000	2.513886000
H	-1.116496000	4.979813000	3.105258000
C	-0.382294000	2.967747000	2.980501000
H	0.156182000	3.034016000	3.919662000
C	-0.353020000	1.791891000	2.226616000
O	0.340887000	0.682449000	2.570400000
C	1.119085000	0.690854000	3.753918000
H	1.892415000	1.467540000	3.715277000
H	1.590807000	-0.290858000	3.801787000
H	0.489359000	0.838314000	4.640242000
C	-3.048471000	-1.867655000	2.849473000
C	1.762671000	1.768654000	-0.743966000
H	1.334188000	2.270650000	0.130440000
H	2.850780000	1.858179000	-0.683878000
H	-3.166613000	-3.989428000	3.218643000
H	-2.742414000	0.171860000	2.250266000
H	-1.543271000	-4.553903000	1.400698000
C	3.144224000	-0.768757000	-1.804930000
C	3.589028000	-0.947238000	-0.522316000
H	3.414542000	-1.890091000	-0.009579000
H	2.631197000	-1.577721000	-2.318153000
H	-3.742107000	-1.602059000	3.643445000
H	3.453037000	0.089278000	-2.394215000
C	4.611327000	-0.073198000	0.140187000
F	5.730284000	-0.788443000	0.368820000
F	4.964624000	0.992856000	-0.603679000
F	4.189142000	0.388036000	1.333700000
C	1.256890000	2.397428000	-2.032514000
H	1.619922000	1.835329000	-2.904323000

H	0.162484000	2.356379000	-2.078082000
C	1.705911000	3.858957000	-2.159039000
H	1.336529000	4.306523000	-3.089712000
H	1.326492000	4.458670000	-1.323142000
H	2.799615000	3.940743000	-2.157405000

65

A1,21(Coor4)

Pd	-1.633969000	0.160859000	-0.007725000
P	0.885920000	-0.106819000	0.054819000
C	1.760260000	1.502229000	-0.148180000
C	1.213941000	2.511540000	-0.960437000
S	-0.361192000	2.304251000	-1.810390000
O	-1.361496000	2.166354000	-0.649966000
O	-0.267687000	1.033298000	-2.555692000
O	-0.602335000	3.533220000	-2.564524000
C	1.862708000	3.735681000	-1.109990000
C	3.058469000	3.978289000	-0.439020000
C	2.951605000	1.771887000	0.534261000
C	1.595916000	-1.264031000	-1.174999000
C	0.705830000	-1.851935000	-2.081002000
H	-0.345746000	-1.592132000	-2.023536000
C	1.143686000	-2.727600000	-3.071925000
H	0.430446000	-3.165419000	-3.763489000
C	2.500422000	-3.012905000	-3.173651000
H	2.862470000	-3.688723000	-3.944397000
C	3.416195000	-2.431094000	-2.298198000
H	4.471256000	-2.660243000	-2.400291000
C	2.973429000	-1.555111000	-1.302272000
O	3.805992000	-0.957595000	-0.414640000
C	5.199473000	-1.153140000	-0.551265000
H	5.475806000	-2.205154000	-0.402466000
H	5.663118000	-0.546883000	0.228687000
H	5.556604000	-0.816528000	-1.532495000
C	1.403790000	-0.745624000	1.693943000
C	1.959207000	-2.014613000	1.878530000
H	2.197455000	-2.619082000	1.008889000
C	2.213897000	-2.517247000	3.155145000
H	2.649257000	-3.505521000	3.271949000
C	1.913392000	-1.741851000	4.270074000
H	2.114532000	-2.118040000	5.269859000
C	1.342653000	-0.477237000	4.120776000
H	1.105074000	0.113671000	4.998971000
C	1.074974000	0.014464000	2.840563000
O	0.485528000	1.208724000	2.592485000
C	0.145055000	2.052804000	3.675621000

H	-0.581587000	1.573911000	4.344737000
H	-0.303985000	2.939218000	3.226662000
H	1.034726000	2.342144000	4.249143000
C	3.597445000	2.997716000	0.390841000
H	3.561851000	4.934193000	-0.558848000
H	3.375146000	1.010397000	1.180571000
H	1.408134000	4.484896000	-1.750235000
H	4.524610000	3.183468000	0.927604000
C	-3.630661000	0.669861000	-0.184270000
H	-4.257262000	-0.220460000	-0.106844000
H	-3.624161000	1.034543000	-1.217362000
C	-2.232084000	-1.160732000	1.582208000
H	-3.268547000	-0.992630000	1.855286000
C	-1.902283000	-1.929652000	0.471814000
H	-1.488684000	-0.983897000	2.354459000
H	-0.913547000	-2.374773000	0.398445000
C	-2.910087000	-2.686486000	-0.351339000
F	-2.668588000	-4.009057000	-0.233549000
F	-2.832929000	-2.388320000	-1.661465000
F	-4.181128000	-2.481162000	0.038389000
C	-4.025459000	1.753838000	0.798718000
H	-3.991399000	1.371601000	1.829168000
H	-3.309908000	2.580669000	0.736093000
C	-5.437370000	2.284533000	0.510566000
H	-6.186377000	1.485612000	0.573085000
H	-5.715890000	3.064871000	1.229191000
H	-5.493163000	2.718404000	-0.494294000

65

A1,21(TS2)

Pd	-1.622427000	0.111224000	0.062885000
P	0.748168000	-0.044546000	0.031831000
C	1.545377000	1.536892000	0.550519000
C	1.033124000	2.781832000	0.147953000
S	-0.453008000	2.924420000	-0.858864000
O	-1.507984000	2.239103000	0.019839000
O	-0.199966000	2.157717000	-2.088734000
O	-0.741198000	4.353833000	-0.984282000
C	1.652319000	3.966694000	0.541189000
C	2.784967000	3.930139000	1.349948000
C	2.677268000	1.519467000	1.373578000
C	1.502261000	-0.496312000	-1.569826000
C	0.647845000	-0.676671000	-2.661452000
H	-0.419668000	-0.561790000	-2.510812000
C	1.140636000	-0.969021000	-3.929734000
H	0.455328000	-1.100519000	-4.761317000

C	2.514473000	-1.069878000	-4.118307000
H	2.917639000	-1.291428000	-5.103349000
C	3.393840000	-0.880926000	-3.053657000
H	4.462310000	-0.957380000	-3.222452000
C	2.896134000	-0.589874000	-1.780020000
O	3.689971000	-0.397803000	-0.696969000
C	5.091713000	-0.375022000	-0.876737000
H	5.471422000	-1.348764000	-1.212892000
H	5.513545000	-0.144000000	0.102834000
H	5.390539000	0.400853000	-1.592488000
C	1.317939000	-1.279900000	1.259342000
C	1.993759000	-2.446696000	0.897465000
H	2.253422000	-2.605282000	-0.143507000
C	2.328503000	-3.412259000	1.846574000
H	2.856222000	-4.311353000	1.542048000
C	1.982019000	-3.213543000	3.179064000
H	2.241766000	-3.955989000	3.929367000
C	1.289068000	-2.067079000	3.568311000
H	1.016067000	-1.930105000	4.609157000
C	0.946123000	-1.107760000	2.611682000
O	0.244418000	0.018939000	2.884386000
C	-0.118619000	0.299665000	4.222350000
H	-0.784584000	-0.472794000	4.628484000
H	-0.645384000	1.254063000	4.190106000
H	0.766905000	0.392312000	4.863709000
C	3.293323000	2.703701000	1.771105000
H	3.263066000	4.857495000	1.655019000
H	3.081965000	0.567443000	1.700734000
H	1.219855000	4.905321000	0.209827000
H	4.172499000	2.664027000	2.409635000
C	-3.785311000	0.787248000	0.150932000
C	-3.189584000	-1.377378000	0.603156000
H	-3.970245000	-1.628100000	-0.104156000
C	-1.898247000	-1.924673000	0.374418000
H	-3.518708000	-1.226086000	1.626503000
H	-1.285196000	-2.222621000	1.222853000
C	-1.744407000	-2.847092000	-0.797961000
F	-2.320622000	-4.045275000	-0.540092000
F	-0.456178000	-3.100134000	-1.090460000
F	-2.333952000	-2.368550000	-1.913477000
C	-5.232572000	0.343710000	0.078862000
H	-5.408457000	-0.256795000	-0.823717000
H	-5.515620000	-0.277486000	0.938797000
C	-6.148046000	1.575020000	0.038110000
H	-5.925290000	2.207794000	-0.827914000

H	-7.198967000	1.269283000	-0.026252000
H	-6.028370000	2.188542000	0.938222000
H	-3.578426000	1.386016000	1.041135000
H	-3.514973000	1.348174000	-0.746794000

65

A1,21(P2)

Pd	-1.158666000	0.570847000	-0.799658000
P	0.762344000	-0.150716000	0.157157000
C	1.749426000	1.308966000	0.694221000
C	1.815372000	2.488022000	-0.069850000
S	0.944379000	2.696030000	-1.641085000
O	-0.524445000	2.534922000	-1.233636000
O	1.391548000	1.584582000	-2.495966000
O	1.215088000	4.062265000	-2.088327000
C	2.575974000	3.569556000	0.370606000
C	3.269663000	3.499617000	1.576016000
C	2.444922000	1.258210000	1.908224000
C	1.824723000	-1.171051000	-0.916197000
C	1.392470000	-1.444055000	-2.217028000
H	0.432340000	-1.058531000	-2.539660000
C	2.180167000	-2.173907000	-3.102144000
H	1.824237000	-2.370378000	-4.108579000
C	3.427843000	-2.627214000	-2.688364000
H	4.058378000	-3.192872000	-3.369714000
C	3.892386000	-2.358546000	-1.401989000
H	4.870876000	-2.715579000	-1.100421000
C	3.099069000	-1.628528000	-0.512682000
O	3.477034000	-1.332015000	0.755511000
C	4.783877000	-1.674697000	1.173309000
H	4.930337000	-2.762521000	1.185412000
H	4.882629000	-1.287442000	2.188654000
H	5.542612000	-1.209730000	0.531946000
C	0.419030000	-1.119043000	1.667211000
C	0.754890000	-2.470670000	1.772181000
H	1.288634000	-2.947653000	0.957237000
C	0.406702000	-3.214690000	2.898150000
H	0.680163000	-4.263804000	2.960682000
C	-0.291117000	-2.602830000	3.934922000
H	-0.563867000	-3.170597000	4.820840000
C	-0.659462000	-1.260558000	3.848579000
H	-1.214170000	-0.801317000	4.659653000
C	-0.319618000	-0.520395000	2.712810000
O	-0.665179000	0.774203000	2.520432000
C	-1.334492000	1.471878000	3.554229000
H	-2.313885000	1.026303000	3.770291000

H	-1.470868000	2.487966000	3.182377000
H	-0.732927000	1.495355000	4.471480000
C	3.199831000	2.342759000	2.347739000
H	3.857787000	4.349831000	1.911881000
H	2.397149000	0.357550000	2.511423000
H	2.600664000	4.461127000	-0.247746000
H	3.732325000	2.279380000	3.293484000
C	-4.500275000	0.186238000	0.285988000
H	-4.957415000	-0.739856000	0.661612000
H	-3.918684000	0.608219000	1.117123000
C	-3.545371000	-0.180290000	-0.851023000
H	-3.181286000	0.799146000	-1.303562000
C	-2.351756000	-1.023192000	-0.441732000
H	-4.083123000	-0.628812000	-1.695874000
H	-2.348276000	-1.314151000	0.611804000
C	-2.151539000	-2.240704000	-1.291982000
F	-3.212140000	-3.083727000	-1.209229000
F	-1.068539000	-2.952797000	-0.926007000
F	-2.014678000	-1.935280000	-2.603225000
C	-5.592192000	1.170810000	-0.130865000
H	-5.124507000	2.092796000	-0.504631000
H	-6.157042000	0.750234000	-0.974744000
C	-6.552087000	1.510629000	1.007065000
H	-7.321557000	2.218325000	0.679800000
H	-7.061406000	0.613305000	1.379312000
H	-6.020525000	1.965713000	1.851864000

64

A2,12(Coor3)

Pd	-1.406447000	0.439044000	0.247964000
P	0.982592000	0.386178000	0.351985000
C	1.765804000	-1.279188000	0.306507000
C	1.135971000	-2.422137000	0.826048000
S	-0.528085000	-2.386555000	1.496480000
O	-1.350797000	-1.789209000	0.322930000
O	-0.529819000	-1.444278000	2.624478000
O	-0.926314000	-3.770924000	1.738166000
C	1.789842000	-3.651095000	0.811808000
C	3.071417000	-3.757589000	0.280571000
C	3.045267000	-1.417053000	-0.241646000
C	1.678622000	1.151067000	1.866561000
C	0.841501000	1.942185000	2.660836000
H	-0.196987000	2.070655000	2.371893000
C	1.325186000	2.531566000	3.827068000
H	0.663056000	3.138217000	4.439116000
C	2.647460000	2.327429000	4.215753000

H	3.022977000	2.780301000	5.129636000
C	3.483928000	1.525123000	3.439413000
H	4.510773000	1.347425000	3.747891000
C	3.003103000	0.935154000	2.273244000
C	1.745555000	1.269700000	-1.076247000
C	2.673605000	2.288765000	-0.820721000
H	2.943150000	2.516770000	0.205249000
C	3.264063000	3.018890000	-1.851826000
H	3.980199000	3.800716000	-1.613289000
C	2.932896000	2.739898000	-3.171036000
H	3.386350000	3.296821000	-3.986350000
C	2.001694000	1.741076000	-3.449768000
H	1.716551000	1.514170000	-4.471430000
C	1.400240000	1.011809000	-2.424610000
C	3.715864000	-2.644476000	-0.263932000
C	5.077417000	-2.763435000	-0.895048000
H	4.988982000	-3.024888000	-1.957387000
H	5.634213000	-1.822538000	-0.836007000
H	5.674974000	-3.544429000	-0.413574000
H	3.570063000	-4.724133000	0.277731000
H	3.531640000	-0.550879000	-0.683318000
H	1.274492000	-4.517950000	1.212913000
C	-3.147373000	0.446335000	1.588645000
H	-3.105072000	1.397286000	2.106750000
C	-3.616902000	0.367198000	0.289433000
H	-3.945786000	1.251500000	-0.248936000
H	3.655892000	0.293098000	1.689274000
S	0.165444000	-0.193876000	-3.000415000
O	0.161544000	-0.085290000	-4.457606000
O	-1.140080000	0.183799000	-2.338283000
O	0.536934000	-1.539493000	-2.448860000
Li	-1.220375000	-1.729362000	-1.554806000
C	-1.392676000	2.469978000	-0.121747000
H	-1.709943000	2.426549000	-1.171432000
H	-0.351039000	2.804938000	-0.098115000
C	-2.252886000	3.416028000	0.698053000
H	-3.310267000	3.123145000	0.673349000
H	-1.949603000	3.403175000	1.753974000
C	-2.137929000	4.853197000	0.171783000
H	-2.461710000	4.917058000	-0.873434000
H	-2.761160000	5.536197000	0.760823000
H	-1.103238000	5.211596000	0.222908000
H	-3.027905000	-0.446695000	2.195143000
C	-4.181327000	-0.904251000	-0.291682000
F	-5.451400000	-0.680191000	-0.679674000

F	-4.187509000	-1.926679000	0.562086000
F	-3.521460000	-1.318210000	-1.416134000

64

A2,12(Coor4)

Pd	-1.632426000	0.201988000	-0.331583000
P	0.822212000	-0.411432000	0.353755000
C	1.924847000	-0.294679000	-1.128993000
C	1.468622000	-0.684161000	-2.399685000
S	-0.190383000	-1.328010000	-2.650888000
O	-1.072227000	-0.107666000	-2.374320000
O	-0.360151000	-2.359265000	-1.609088000
O	-0.282565000	-1.739534000	-4.051827000
C	2.306076000	-0.590440000	-3.507811000
C	3.601152000	-0.102396000	-3.370382000
C	3.232240000	0.191914000	-1.017405000
C	1.239636000	-2.043777000	1.094632000
C	0.780727000	-2.348976000	2.384681000
H	0.221841000	-1.611582000	2.955296000
C	1.043039000	-3.589567000	2.958429000
H	0.680857000	-3.806848000	3.960042000
C	1.755599000	-4.552171000	2.243564000
H	1.954391000	-5.524819000	2.686297000
C	2.203185000	-4.262915000	0.956706000
H	2.749377000	-5.010425000	0.387347000
C	1.949589000	-3.017262000	0.384160000
C	1.691033000	0.761001000	1.538840000
C	2.624360000	0.234628000	2.449318000
H	2.817003000	-0.831961000	2.444352000
C	3.323162000	1.031211000	3.352727000
H	4.035805000	0.569363000	4.030946000
C	3.122517000	2.407026000	3.370412000
H	3.670935000	3.042872000	4.059714000
C	2.219226000	2.965471000	2.474711000
H	2.081817000	4.041194000	2.439255000
C	1.509564000	2.160022000	1.580313000
C	4.083435000	0.299334000	-2.122203000
H	4.245122000	-0.035024000	-4.244841000
H	3.609756000	0.497448000	-0.045999000
H	1.920714000	-0.910323000	-4.470870000
C	-3.198282000	1.162013000	-1.276616000
C	-3.172881000	-0.451059000	1.001702000
C	-2.306483000	0.395102000	1.695656000
H	-4.129701000	-0.079214000	0.649356000
H	-2.550799000	1.440971000	1.848197000
C	-3.813288000	2.331764000	-0.539799000

H	-4.368268000	2.005417000	0.350488000
H	-3.020215000	3.002015000	-0.186998000
C	-4.772844000	3.107322000	-1.455071000
H	-5.590849000	2.467109000	-1.805279000
H	-5.216939000	3.960180000	-0.926689000
H	-4.250153000	3.489770000	-2.339283000
H	-2.638493000	1.478992000	-2.160326000
H	-3.921120000	0.391500000	-1.564100000
C	5.474655000	0.856098000	-1.968956000
H	6.185288000	0.345649000	-2.628147000
H	5.500606000	1.923161000	-2.226417000
H	5.836357000	0.758860000	-0.940103000
H	2.289620000	-2.811722000	-0.624605000
S	0.369637000	3.047085000	0.515355000
O	-0.873111000	3.340399000	1.342245000
Li	-0.533096000	5.177343000	1.106519000
O	0.975329000	4.413635000	0.273878000
O	0.145143000	2.239234000	-0.683504000
H	-1.556522000	-0.018357000	2.362667000
C	-3.209609000	-1.940048000	1.190667000
F	-4.129851000	-2.241190000	2.140932000
F	-3.584761000	-2.569775000	0.069942000
F	-2.041144000	-2.453326000	1.596733000

64

A2,12(TS2)

Pd	-1.361517000	-0.017405000	-0.301112000
P	0.795660000	-0.359158000	0.496869000
C	2.048096000	-0.631992000	-0.818433000
C	1.715938000	-1.364442000	-1.970023000
S	0.077058000	-2.071472000	-2.231812000
O	-0.839835000	-0.846670000	-2.224771000
O	-0.168516000	-2.929715000	-1.059490000
O	0.102819000	-2.701401000	-3.553125000
C	2.680372000	-1.586412000	-2.949013000
C	3.969894000	-1.086884000	-2.796258000
C	3.347981000	-0.132942000	-0.686139000
C	0.950412000	-1.849347000	1.558770000
C	-0.191535000	-2.368526000	2.178188000
H	-1.160707000	-1.918381000	1.989610000
C	-0.100024000	-3.496456000	2.990869000
H	-0.996615000	-3.896268000	3.457059000
C	1.129395000	-4.123599000	3.182527000
H	1.197825000	-5.009840000	3.808270000
C	2.268571000	-3.624647000	2.551188000
H	3.226438000	-4.122496000	2.678682000

C	2.181061000	-2.495331000	1.740798000
C	1.512867000	1.000868000	1.556695000
C	2.023926000	0.655643000	2.818067000
H	1.995953000	-0.381664000	3.130911000
C	2.579400000	1.598066000	3.681049000
H	2.963983000	1.276931000	4.645338000
C	2.654793000	2.932006000	3.299707000
H	3.107042000	3.673888000	3.951973000
C	2.143008000	3.310985000	2.063149000
H	2.211360000	4.342552000	1.734040000
C	1.561875000	2.370281000	1.210904000
C	4.324150000	-0.348593000	-1.664113000
C	5.708275000	0.225800000	-1.512690000
H	5.809146000	1.158902000	-2.082193000
H	5.938419000	0.454268000	-0.466860000
H	6.471846000	-0.466199000	-1.884767000
H	4.710746000	-1.271947000	-3.571450000
H	3.612113000	0.448575000	0.193681000
H	2.395906000	-2.155772000	-3.828310000
C	-3.315475000	0.291181000	-1.409086000
H	-3.025185000	-0.661656000	-1.862131000
H	-4.370429000	0.241136000	-1.154842000
C	-2.012698000	0.886928000	1.422573000
H	-1.582487000	1.884547000	1.470414000
C	-3.256236000	0.744340000	0.727624000
H	-3.752394000	1.659970000	0.417458000
H	3.069427000	-2.125734000	1.235933000
S	0.845859000	3.048427000	-0.294147000
O	0.902922000	2.029119000	-1.333272000
O	1.598754000	4.332389000	-0.582873000
O	-0.549865000	3.525889000	0.080150000
Li	-0.022094000	5.269662000	-0.367062000
C	-2.990092000	1.500465000	-2.261921000
H	-3.188826000	2.426218000	-1.703463000
H	-1.928441000	1.508639000	-2.527987000
C	-3.831509000	1.486614000	-3.546056000
H	-3.605849000	2.364509000	-4.162958000
H	-3.614592000	0.593813000	-4.141999000
H	-4.905935000	1.495609000	-3.324639000
H	-1.829954000	0.258915000	2.292777000
C	-4.215930000	-0.271464000	1.304093000
F	-4.523801000	0.064528000	2.576974000
F	-3.696041000	-1.510893000	1.337777000
F	-5.385007000	-0.336181000	0.637734000

64

A2,12(P2)

Pd	-1.123341000	-0.224379000	-0.491413000
P	0.871920000	-0.258949000	0.496257000
C	2.228397000	-0.555984000	-0.699229000
C	2.048935000	-1.402027000	-1.806134000
S	0.500939000	-2.272987000	-2.156202000
O	-0.516791000	-1.141510000	-2.307362000
O	0.237015000	-3.077113000	-0.950037000
O	0.705074000	-2.977863000	-3.422049000
C	3.114300000	-1.628056000	-2.673555000
C	4.348409000	-1.024219000	-2.455293000
C	3.475271000	0.046722000	-0.497722000
C	1.031241000	-1.627186000	1.706409000
C	-0.114113000	-2.214922000	2.253615000
H	-1.097663000	-1.879390000	1.942245000
C	0.003703000	-3.259879000	3.167610000
H	-0.893310000	-3.715828000	3.577627000
C	1.261849000	-3.733277000	3.533945000
H	1.350703000	-4.555822000	4.238972000
C	2.407234000	-3.163589000	2.978164000
H	3.390898000	-3.541936000	3.243931000
C	2.295016000	-2.118160000	2.065402000
C	1.349423000	1.263312000	1.452987000
C	1.709060000	1.095936000	2.799585000
H	1.703865000	0.099980000	3.227268000
C	2.085859000	2.167612000	3.606495000
H	2.359677000	1.985276000	4.642073000
C	2.124611000	3.453003000	3.081079000
H	2.438296000	4.295181000	3.691561000
C	1.755791000	3.654156000	1.754701000
H	1.798921000	4.646174000	1.317713000
C	1.352862000	2.585529000	0.952868000
C	4.549320000	-0.174310000	-1.364860000
H	5.166357000	-1.213872000	-3.147310000
H	3.617830000	0.717275000	0.346102000
H	2.949682000	-2.284031000	-3.522517000
C	-3.744296000	1.407788000	-0.605665000
C	-3.482472000	0.735720000	0.761782000
C	-1.981660000	0.746112000	1.082729000
H	-4.015558000	1.310446000	1.536588000
H	-1.617168000	1.777277000	1.073555000
C	-5.210070000	1.654972000	-1.017244000
H	-5.838634000	1.720076000	-0.119516000
H	-5.267952000	2.640828000	-1.496630000
C	-5.788698000	0.619370000	-1.982267000

H	-5.787916000	-0.385540000	-1.552737000
H	-6.822887000	0.868351000	-2.246730000
H	-5.206785000	0.586222000	-2.911294000
H	-3.205143000	2.359684000	-0.561308000
H	-3.242843000	0.838727000	-1.407914000
C	5.872398000	0.511185000	-1.146074000
H	6.711199000	-0.161491000	-1.356805000
H	5.977261000	1.379751000	-1.809124000
H	5.976031000	0.870331000	-0.116954000
H	3.191404000	-1.696160000	1.619711000
S	0.800559000	3.041360000	-0.701120000
O	-0.657271000	3.449291000	-0.565805000
Li	-0.182475000	5.165243000	-1.165524000
O	1.508779000	4.339732000	-1.035642000
O	1.062443000	1.924014000	-1.596694000
H	-1.774937000	0.301338000	2.059847000
C	-4.045097000	-0.670594000	0.863603000
F	-5.392075000	-0.694338000	0.843308000
F	-3.619454000	-1.457242000	-0.157654000
F	-3.670761000	-1.281154000	2.008749000

64

A2,21(Coor3)

Pd	1.362502000	-0.509689000	-0.026471000
P	-0.793330000	0.211148000	0.579266000
C	-1.478087000	1.550357000	-0.468695000
C	-0.631564000	2.543216000	-0.986179000
S	1.139846000	2.553669000	-0.651471000
O	1.621182000	1.247734000	-1.276214000
O	1.239232000	2.504077000	0.821284000
O	1.687217000	3.722433000	-1.340570000
C	-1.162809000	3.567005000	-1.764384000
C	-2.527482000	3.612467000	-2.032331000
C	-2.846615000	1.609537000	-0.751615000
C	-0.858354000	0.895508000	2.280503000
C	0.188029000	0.619355000	3.167096000
H	1.034968000	0.029358000	2.828876000
C	0.169142000	1.137374000	4.459869000
H	0.992861000	0.925415000	5.136261000
C	-0.888431000	1.944804000	4.874673000
H	-0.897301000	2.356684000	5.880583000
C	-1.924643000	2.241840000	3.989725000
H	-2.740543000	2.889214000	4.300700000
C	-1.909421000	1.723794000	2.696894000
C	-2.102447000	-1.118881000	0.583564000
C	-2.828121000	-1.323313000	1.768133000

H	-2.614072000	-0.703620000	2.631080000
C	-3.825279000	-2.290629000	1.874180000
H	-4.359554000	-2.405362000	2.813427000
C	-4.140649000	-3.087492000	0.780627000
H	-4.931909000	-3.829538000	0.840741000
C	-3.426934000	-2.924151000	-0.401679000
H	-3.669722000	-3.521186000	-1.274474000
C	-2.409452000	-1.973530000	-0.498974000
C	-3.391200000	2.633765000	-1.532882000
C	-4.863151000	2.661470000	-1.851240000
H	-5.057072000	2.235987000	-2.844566000
H	-5.444149000	2.080250000	-1.127649000
H	-5.252645000	3.685438000	-1.854731000
H	-2.926700000	4.421066000	-2.641242000
H	-3.510036000	0.838721000	-0.366794000
H	-0.484508000	4.321500000	-2.150469000
C	3.543401000	-1.047507000	-0.469112000
H	3.836977000	-1.651849000	0.381398000
C	2.730163000	-1.548463000	-1.451630000
H	2.584176000	-0.998885000	-2.377503000
H	2.348453000	-2.563473000	-1.406566000
H	-2.708276000	1.978122000	2.005589000
S	-1.511699000	-1.981703000	-2.058933000
O	-0.941743000	-0.657307000	-2.264703000
O	-2.496910000	-2.454203000	-3.110074000
O	-0.504977000	-3.118427000	-1.970440000
Li	-1.378613000	-3.940780000	-3.413854000
C	0.989525000	-2.240403000	1.061021000
H	0.472390000	-2.878758000	0.334577000
H	0.273255000	-2.000442000	1.855933000
C	2.170015000	-2.973312000	1.686714000
H	2.846479000	-3.359921000	0.912712000
H	2.762840000	-2.293645000	2.314983000
C	1.702054000	-4.155788000	2.544465000
H	1.125561000	-4.871386000	1.945849000
H	2.553754000	-4.690436000	2.982394000
H	1.059285000	-3.817103000	3.365890000
C	4.441935000	0.148413000	-0.650354000
F	5.723184000	-0.291890000	-0.534961000
F	4.265238000	1.071773000	0.304108000
F	4.330032000	0.729689000	-1.844137000

64

A2,21(Coor4)

Pd	1.653692000	-0.219557000	0.360833000
P	-0.769506000	0.067925000	-0.560971000

C	-1.900174000	-1.255314000	0.044271000
C	-1.391092000	-2.525951000	0.360156000
S	0.370136000	-2.880935000	0.314245000
O	0.942222000	-2.010567000	1.425601000
O	0.867686000	-2.338414000	-0.985181000
O	0.526675000	-4.314953000	0.550119000
C	-2.247515000	-3.543275000	0.771191000
C	-3.614223000	-3.308039000	0.880991000
C	-3.276024000	-1.041071000	0.168805000
C	-0.798578000	-0.224630000	-2.382949000
C	0.343346000	0.078836000	-3.133050000
H	1.238214000	0.442225000	-2.639811000
C	0.361331000	-0.124507000	-4.511780000
H	1.260412000	0.107840000	-5.076459000
C	-0.759186000	-0.643041000	-5.157463000
H	-0.742325000	-0.810286000	-6.231469000
C	-1.896864000	-0.963073000	-4.417103000
H	-2.769392000	-1.383964000	-4.910573000
C	-1.917028000	-0.757090000	-3.039472000
C	-1.776289000	1.641482000	-0.412433000
C	-2.226331000	2.261161000	-1.591763000
H	-2.011639000	1.795201000	-2.546213000
C	-2.954456000	3.448513000	-1.581686000
H	-3.281108000	3.880809000	-2.523747000
C	-3.276028000	4.061633000	-0.376011000
H	-3.864409000	4.974628000	-0.353314000
C	-2.832515000	3.486990000	0.809224000
H	-3.087903000	3.934844000	1.764191000
C	-2.079035000	2.311547000	0.794097000
C	-4.148788000	-2.050279000	0.590529000
H	-4.272526000	-4.112694000	1.201377000
H	-3.687103000	-0.061019000	-0.061256000
H	-1.822379000	-4.515219000	1.000944000
C	3.509210000	-0.954612000	0.866532000
C	2.340561000	1.663854000	1.068958000
H	3.294607000	1.577668000	1.579203000
C	2.306120000	1.707627000	-0.330237000
H	1.506610000	2.039308000	1.658036000
H	1.449631000	2.155296000	-0.828719000
C	3.543664000	1.813772000	-1.176113000
F	4.687006000	1.699684000	-0.473723000
F	3.572955000	3.022852000	-1.779634000
F	3.586405000	0.892093000	-2.160447000
C	3.714958000	-1.188159000	2.347585000
H	3.763986000	-0.233426000	2.888498000

H	2.865946000	-1.746934000	2.751486000
C	5.012583000	-1.971290000	2.598036000
H	5.888343000	-1.435376000	2.211757000
H	5.163836000	-2.134846000	3.671733000
H	4.977815000	-2.952732000	2.111553000
H	3.432486000	-1.890235000	0.300267000
H	4.272372000	-0.310718000	0.427190000
C	-5.620792000	-1.775318000	0.751358000
H	-6.222197000	-2.659312000	0.513596000
H	-5.853152000	-1.490620000	1.785961000
H	-5.951426000	-0.955094000	0.105567000
H	-2.803725000	-1.023764000	-2.471767000
S	-1.474394000	1.774603000	2.395273000
O	-0.286867000	2.673316000	2.718380000
Li	-1.215944000	3.273091000	4.235514000
O	-2.517001000	2.179600000	3.416760000
O	-1.167052000	0.353639000	2.312271000

64

A2,21(TS2)

Pd	1.526744000	0.614637000	0.243334000
P	-0.663532000	-0.247329000	0.421988000
C	-1.935729000	0.835090000	-0.351892000
C	-1.867292000	2.229686000	-0.204983000
S	-0.525780000	3.043714000	0.678747000
O	0.718041000	2.573766000	-0.083212000
O	-0.549111000	2.503267000	2.047109000
O	-0.723045000	4.483519000	0.502682000
C	-2.865248000	3.034859000	-0.747112000
C	-3.933415000	2.467632000	-1.434374000
C	-3.014501000	0.285490000	-1.052016000
C	-1.332830000	-0.455496000	2.120773000
C	-0.468149000	-0.387479000	3.216823000
H	0.588866000	-0.209443000	3.060216000
C	-0.964670000	-0.515319000	4.512098000
H	-0.282281000	-0.453376000	5.355511000
C	-2.328679000	-0.700880000	4.725640000
H	-2.714815000	-0.793497000	5.737641000
C	-3.201180000	-0.747189000	3.638427000
H	-4.269344000	-0.870224000	3.798247000
C	-2.708089000	-0.618074000	2.342770000
C	-0.932687000	-1.910259000	-0.382798000
C	-1.483122000	-2.937493000	0.399455000
H	-1.721831000	-2.736844000	1.437067000
C	-1.741083000	-4.207381000	-0.110953000
H	-2.170724000	-4.965411000	0.538297000

C	-1.464761000	-4.492405000	-1.442608000
H	-1.685684000	-5.470102000	-1.861696000
C	-0.894703000	-3.505581000	-2.239301000
H	-0.684955000	-3.698207000	-3.286178000
C	-0.608804000	-2.241687000	-1.718150000
C	-4.022713000	1.083549000	-1.602070000
C	-5.155413000	0.466066000	-2.379417000
H	-4.954681000	0.501209000	-3.458189000
H	-5.305260000	-0.584421000	-2.109076000
H	-6.096857000	0.998812000	-2.206100000
H	-4.705855000	3.111406000	-1.849980000
H	-3.074662000	-0.791601000	-1.185816000
H	-2.781353000	4.109675000	-0.621513000
C	3.367192000	1.875003000	-0.146656000
H	2.855605000	2.586706000	0.507100000
H	4.396043000	1.769849000	0.195866000
C	2.519014000	-1.207462000	0.363642000
H	2.182965000	-1.819291000	-0.470308000
C	3.595137000	-0.306080000	0.130324000
H	4.033530000	-0.288232000	-0.861674000
H	-3.398431000	-0.630284000	1.503595000
S	0.255709000	-1.138960000	-2.847447000
O	-0.085216000	0.236935000	-2.512802000
O	-0.105895000	-1.590982000	-4.248420000
O	1.732028000	-1.492748000	-2.725234000
Li	1.712280000	-2.002434000	-4.530043000
C	3.287596000	2.242175000	-1.612653000
H	3.773535000	1.470302000	-2.226076000
H	2.243449000	2.304584000	-1.931308000
C	3.970723000	3.594221000	-1.861680000
H	3.936237000	3.851312000	-2.926986000
H	3.463079000	4.391213000	-1.308145000
H	5.023353000	3.579996000	-1.552077000
H	4.282121000	-0.128767000	0.951365000
C	2.510483000	-1.942632000	1.667127000
F	3.436532000	-2.931965000	1.659762000
F	1.327627000	-2.538493000	1.919584000
F	2.804521000	-1.152614000	2.722085000

64

A2,21(P2)

Pd	-1.295063000	-0.510847000	-0.402000000
P	0.725755000	-0.110047000	0.491675000
C	2.070999000	-0.659972000	-0.630371000
C	1.957785000	-1.845023000	-1.376370000
S	0.500680000	-2.914248000	-1.332572000

O	-0.633005000	-1.979472000	-1.765010000
O	0.351236000	-3.319846000	0.074332000
O	0.726589000	-3.959182000	-2.331215000
C	3.021626000	-2.256846000	-2.174380000
C	4.192901000	-1.508453000	-2.237421000
C	3.254518000	0.081835000	-0.710023000
C	1.084411000	-1.028148000	2.036463000
C	0.054667000	-1.677591000	2.722269000
H	-0.958624000	-1.633823000	2.342125000
C	0.334281000	-2.405431000	3.876562000
H	-0.473228000	-2.912488000	4.397358000
C	1.641479000	-2.499002000	4.348284000
H	1.857414000	-3.073801000	5.245293000
C	2.677044000	-1.870171000	3.656732000
H	3.702081000	-1.956070000	4.007864000
C	2.403223000	-1.143896000	2.501307000
C	1.087372000	1.669775000	0.885467000
C	1.442310000	1.976771000	2.208387000
H	1.480708000	1.180167000	2.941729000
C	1.751521000	3.273299000	2.611692000
H	2.022167000	3.459436000	3.647310000
C	1.726150000	4.312943000	1.690086000
H	1.990228000	5.324849000	1.984329000
C	1.350289000	4.045540000	0.378079000
H	1.336179000	4.840520000	-0.360077000
C	1.009879000	2.751653000	-0.020943000
C	4.327977000	-0.325040000	-1.508321000
H	5.012011000	-1.848871000	-2.867377000
H	3.347503000	1.008828000	-0.149518000
H	2.906937000	-3.172847000	-2.745124000
C	-4.026952000	-0.414639000	-0.839940000
C	-3.541496000	0.983365000	-0.399641000
H	-4.385281000	1.521654000	0.057629000
C	-2.338988000	0.960682000	0.548758000
H	-3.245679000	1.558103000	-1.284270000
H	-1.805823000	1.913196000	0.516035000
C	-2.694937000	0.697437000	1.981490000
F	-3.661186000	1.538464000	2.429497000
F	-1.637057000	0.873467000	2.806442000
F	-3.161819000	-0.557231000	2.201987000
C	-5.127895000	-0.338434000	-1.900033000
H	-5.954282000	0.263485000	-1.494127000
H	-4.747502000	0.209126000	-2.773409000
C	-5.644032000	-1.707897000	-2.332923000
H	-6.058283000	-2.262857000	-1.482692000

H	-6.433882000	-1.613692000	-3.086624000
H	-4.839943000	-2.314847000	-2.765643000
H	-3.214685000	-1.032721000	-1.288923000
H	-4.380146000	-0.980441000	0.029160000
C	5.577068000	0.510998000	-1.603151000
H	6.469110000	-0.114416000	-1.716641000
H	5.534546000	1.180161000	-2.472472000
H	5.712451000	1.137392000	-0.715222000
H	3.219836000	-0.679904000	1.954955000
S	0.396953000	2.620129000	-1.710107000
O	-1.059364000	3.051559000	-1.660197000
Li	-0.630614000	4.469802000	-2.815134000
O	1.076414000	3.725292000	-2.493338000
O	0.627611000	1.263050000	-2.185846000

82

A3,12(Coor3)

Pd	2.212731000	0.179867000	-0.532376000
P	-0.129109000	0.355620000	-0.092817000
C	-0.770485000	-1.202144000	0.643440000
C	-0.306761000	-2.454752000	0.206792000
S	0.883656000	-2.609210000	-1.131000000
O	2.139350000	-1.997078000	-0.513768000
O	0.368496000	-1.774280000	-2.233897000
O	1.049970000	-4.042334000	-1.386292000
C	-0.726913000	-3.616308000	0.846626000
C	-1.603370000	-3.544753000	1.927398000
C	-1.647297000	-1.154679000	1.729857000
C	-1.111404000	0.915725000	-1.547569000
C	-0.329713000	1.480728000	-2.566393000
H	0.750653000	1.429472000	-2.479040000
C	-0.899814000	2.074338000	-3.686674000
H	-0.262696000	2.497029000	-4.458886000
C	-2.284659000	2.106571000	-3.810057000
H	-2.753058000	2.567477000	-4.676048000
C	-3.071422000	1.513616000	-2.828865000
H	-4.152143000	1.497416000	-2.939995000
C	-2.517617000	0.899440000	-1.696198000
C	-0.418848000	1.627806000	1.194334000
C	-1.170452000	2.781547000	0.963900000
H	-1.699651000	2.891998000	0.024292000
C	-1.261740000	3.786699000	1.928633000
H	-1.850411000	4.677251000	1.728221000
C	-0.592851000	3.640396000	3.138642000
H	-0.657905000	4.415469000	3.898312000
C	0.178061000	2.505194000	3.391216000

H	0.703472000	2.409578000	4.335334000
C	0.279178000	1.507271000	2.419221000
O	1.027953000	0.389992000	2.554754000
C	1.737433000	0.172919000	3.760437000
H	2.472063000	0.967654000	3.939301000
H	2.256300000	-0.775790000	3.628552000
H	1.051732000	0.105998000	4.614578000
C	-2.076577000	-2.313940000	2.385719000
C	2.355587000	2.244076000	-0.470197000
H	1.353196000	2.675672000	-0.403094000
H	2.844926000	2.427196000	0.497805000
C	-3.032655000	-2.220118000	3.545465000
H	-2.677946000	-1.508271000	4.300344000
H	-4.017451000	-1.873391000	3.210175000
H	-3.164975000	-3.190630000	4.034209000
H	-1.916883000	-4.460406000	2.424734000
H	-2.011549000	-0.191797000	2.077065000
H	-0.343482000	-4.567740000	0.490899000
C	4.123918000	-0.247994000	-1.638618000
H	4.078041000	0.463506000	-2.456946000
H	4.021346000	-1.301944000	-1.877723000
C	4.490152000	0.151230000	-0.380968000
H	4.758978000	1.182429000	-0.173437000
C	-3.487516000	0.197718000	-0.803783000
C	-3.688969000	-1.185981000	-0.981824000
C	-4.345994000	0.895046000	0.061375000
C	-4.678136000	-1.866204000	-0.261011000
C	-5.327867000	0.220939000	0.798286000
C	-5.477990000	-1.152381000	0.624525000
H	-4.827238000	-2.931472000	-0.392744000
H	-5.980922000	0.753112000	1.480137000
H	-6.249924000	-1.675596000	1.183896000
O	-4.167531000	2.244464000	0.110288000
O	-2.884462000	-1.762748000	-1.896108000
C	-5.016200000	3.000125000	0.947938000
H	-4.891003000	2.727419000	2.003995000
H	-4.722242000	4.042397000	0.810066000
H	-6.070753000	2.883378000	0.665140000
C	-2.989404000	-3.153558000	-2.132614000
H	-2.205476000	-3.383562000	-2.854316000
H	-2.812172000	-3.727586000	-1.215495000
H	-3.972224000	-3.413675000	-2.548674000
C	3.111984000	2.919447000	-1.608483000
H	4.148126000	2.561356000	-1.675589000
H	2.644447000	2.677927000	-2.572963000

C	3.139562000	4.444173000	-1.442337000
H	3.629654000	4.729432000	-0.503647000
H	3.683733000	4.922921000	-2.265451000
H	2.123953000	4.856385000	-1.424479000
C	4.968473000	-0.789769000	0.689655000
F	6.284685000	-0.553066000	0.907107000
F	4.834590000	-2.076834000	0.380888000
F	4.343353000	-0.567884000	1.865557000

82

A3,12(Coor4)

Pd	-2.405329000	0.213900000	-0.039792000
P	0.097860000	-0.239858000	0.117502000
C	1.028219000	1.331781000	0.350495000
C	0.645040000	2.490268000	-0.344588000
S	-0.723168000	2.466849000	-1.503056000
O	-1.912013000	2.198133000	-0.562165000
O	-0.491896000	1.332604000	-2.410960000
O	-0.821655000	3.809101000	-2.078481000
C	1.278850000	3.704069000	-0.097032000
C	2.295917000	3.779142000	0.851227000
C	2.046982000	1.434416000	1.301086000
C	0.814552000	-1.260682000	-1.240681000
C	-0.165210000	-1.919194000	-1.997909000
H	-1.207190000	-1.674848000	-1.829664000
C	0.163085000	-2.846873000	-2.980622000
H	-0.625458000	-3.333594000	-3.547830000
C	1.501719000	-3.125447000	-3.232506000
H	1.783694000	-3.848520000	-3.993769000
C	2.486047000	-2.443023000	-2.525080000
H	3.534119000	-2.620812000	-2.750299000
C	2.177120000	-1.497564000	-1.536673000
C	0.459606000	-1.194449000	1.647083000
C	1.019504000	-2.473136000	1.627038000
H	1.370534000	-2.882426000	0.686702000
C	1.152125000	-3.224706000	2.797021000
H	1.589337000	-4.218142000	2.754382000
C	0.722699000	-2.694689000	4.007636000
H	0.823667000	-3.268285000	4.925611000
C	0.148290000	-1.423295000	4.059173000
H	-0.190457000	-1.024297000	5.009379000
C	0.004709000	-0.680987000	2.884976000
O	-0.577334000	0.541855000	2.829958000
C	-1.007581000	1.156076000	4.028854000
H	-1.794647000	0.570553000	4.521539000
H	-1.408312000	2.126741000	3.735204000

H	-0.170892000	1.300460000	4.724051000
C	2.694496000	2.646065000	1.564855000
C	3.808198000	2.712119000	2.576235000
H	3.589344000	2.096857000	3.456178000
H	4.745173000	2.338374000	2.145121000
H	3.981839000	3.739023000	2.914185000
H	2.779193000	4.734688000	1.044960000
H	2.349860000	0.548163000	1.851639000
H	0.948134000	4.581473000	-0.644269000
C	-4.287263000	1.184999000	-0.028226000
H	-4.085751000	1.909151000	-0.819195000
H	-5.133146000	0.557299000	-0.305417000
C	-2.564449000	-1.775908000	0.796958000
H	-2.385707000	-1.650793000	1.860467000
C	-3.802806000	-1.457642000	0.258865000
H	-4.599035000	-1.124708000	0.916462000
C	3.342264000	-0.754851000	-0.970799000
C	3.672745000	0.499093000	-1.522795000
C	4.236010000	-1.343884000	-0.062034000
C	4.836185000	1.172739000	-1.131558000
C	5.394577000	-0.671359000	0.347218000
C	5.676980000	0.578736000	-0.196571000
H	5.087742000	2.138221000	-1.554796000
H	6.078426000	-1.116784000	1.060217000
H	6.584599000	1.095358000	0.106622000
O	3.906678000	-2.595398000	0.363593000
O	2.806213000	0.957655000	-2.448555000
C	4.789601000	-3.252706000	1.247372000
H	4.883721000	-2.717175000	2.201326000
H	4.352557000	-4.236120000	1.430860000
H	5.785924000	-3.378689000	0.803176000
C	3.047092000	2.209023000	-3.061500000
H	2.196646000	2.374937000	-3.723247000
H	3.087946000	3.015075000	-2.319165000
H	3.978248000	2.194879000	-3.643983000
H	-1.889172000	-2.446967000	0.275421000
C	-4.318236000	-2.121277000	-0.987405000
F	-5.083936000	-3.180795000	-0.639893000
F	-3.334902000	-2.589576000	-1.777360000
F	-5.089312000	-1.316253000	-1.735046000
C	-4.444935000	1.830012000	1.331375000
H	-4.542204000	1.065255000	2.116676000
H	-3.549210000	2.418182000	1.562875000
C	-5.675586000	2.749368000	1.374276000
H	-6.596178000	2.192231000	1.162736000

H	-5.782281000	3.215722000	2.361224000
H	-5.588991000	3.549023000	0.630412000

82

A3,12(TS2)

Pd	-2.190148000	0.173295000	0.010158000
P	0.122953000	-0.246778000	0.106991000
C	1.054940000	1.299756000	0.461355000
C	0.654133000	2.528215000	-0.093895000
S	-0.699382000	2.657303000	-1.274663000
O	-1.915828000	2.257013000	-0.433056000
O	-0.417714000	1.665378000	-2.325089000
O	-0.778736000	4.067019000	-1.666391000
C	1.296718000	3.704083000	0.280297000
C	2.331495000	3.675065000	1.213125000
C	2.095538000	1.295429000	1.393113000
C	0.776465000	-1.158129000	-1.355935000
C	-0.225181000	-1.742245000	-2.147039000
H	-1.264251000	-1.514592000	-1.934371000
C	0.077855000	-2.578764000	-3.215979000
H	-0.724977000	-3.007349000	-3.809622000
C	1.408714000	-2.843381000	-3.519237000
H	1.668948000	-3.496509000	-4.348403000
C	2.412534000	-2.237822000	-2.771120000
H	3.454658000	-2.405732000	-3.028923000
C	2.130769000	-1.382330000	-1.696657000
C	0.476639000	-1.342052000	1.537972000
C	1.005963000	-2.625100000	1.388552000
H	1.333548000	-2.952375000	0.408517000
C	1.132783000	-3.487609000	2.479613000
H	1.545146000	-4.482326000	2.337060000
C	0.727231000	-3.065921000	3.740066000
H	0.823207000	-3.726774000	4.597978000
C	0.183343000	-1.792985000	3.919336000
H	-0.137071000	-1.479498000	4.907096000
C	0.046571000	-0.937215000	2.824294000
O	-0.502187000	0.299165000	2.897113000
C	-0.934712000	0.788793000	4.150983000
H	-1.733629000	0.164786000	4.572101000
H	-1.319410000	1.791060000	3.959100000
H	-0.103227000	0.847403000	4.864862000
C	2.747560000	2.470909000	1.783210000
C	3.869057000	2.420419000	2.787097000
H	3.551150000	1.936769000	3.718888000
H	4.716579000	1.845090000	2.396349000
H	4.227794000	3.424192000	3.036137000

H	2.817811000	4.604314000	1.503195000
H	2.413207000	0.352122000	1.829436000
H	0.961127000	4.636514000	-0.163048000
C	-4.345168000	0.891991000	0.009698000
H	-5.294617000	0.467382000	-0.303162000
H	-3.970328000	1.529659000	-0.796272000
C	-2.656364000	-1.739277000	0.588371000
H	-2.415998000	-1.864567000	1.642631000
C	-3.960641000	-1.256386000	0.254971000
H	-4.675873000	-1.155533000	1.067278000
C	3.313422000	-0.711727000	-1.079365000
C	3.654201000	0.585289000	-1.513408000
C	4.207817000	-1.395632000	-0.240551000
C	4.828565000	1.205796000	-1.070486000
C	5.377981000	-0.778203000	0.219537000
C	5.669914000	0.515603000	-0.204634000
H	5.085886000	2.205603000	-1.399983000
H	6.063393000	-1.297475000	0.879101000
H	6.585340000	0.991557000	0.138900000
O	3.866237000	-2.678075000	0.067435000
O	2.783637000	1.137415000	-2.380472000
C	4.741360000	-3.422131000	0.887936000
H	4.842302000	-2.973838000	1.885109000
H	4.292200000	-4.412437000	0.984564000
H	5.735727000	-3.520947000	0.432643000
C	3.023311000	2.442420000	-2.870747000
H	2.160261000	2.678228000	-3.493358000
H	3.086531000	3.170640000	-2.053382000
H	3.942986000	2.479269000	-3.470351000
C	-4.450543000	1.599414000	1.344539000
H	-4.703103000	0.884539000	2.141249000
H	-3.490893000	2.055760000	1.607524000
C	-5.526550000	2.693402000	1.290617000
H	-5.611414000	3.199551000	2.259459000
H	-5.275332000	3.447922000	0.537676000
H	-6.509632000	2.276621000	1.040149000
H	-2.185707000	-2.472131000	-0.063968000
C	-4.586299000	-1.843935000	-0.990253000
F	-4.708088000	-3.181715000	-0.848705000
F	-5.825919000	-1.374402000	-1.230490000
F	-3.848159000	-1.627370000	-2.092834000

82

A3,12(P2)

Pd	-1.914452000	0.834082000	-0.266490000
P	0.165165000	-0.050593000	-0.207104000

C	1.102751000	0.712312000	1.180928000
C	0.947710000	2.066683000	1.524707000
S	-0.079850000	3.204980000	0.574316000
O	-1.481262000	2.632403000	0.780866000
O	0.368018000	3.065631000	-0.822569000
O	0.061484000	4.517458000	1.208704000
C	1.592568000	2.573654000	2.648773000
C	2.386515000	1.747430000	3.441050000
C	1.910411000	-0.096837000	1.985234000
C	1.033775000	0.074788000	-1.827102000
C	0.201287000	0.423168000	-2.901446000
H	-0.821653000	0.716457000	-2.692075000
C	0.662191000	0.434665000	-4.213050000
H	-0.009903000	0.717517000	-5.018492000
C	1.984893000	0.094974000	-4.473995000
H	2.365413000	0.091408000	-5.492289000
C	2.834005000	-0.206997000	-3.414996000
H	3.880610000	-0.426294000	-3.607018000
C	2.396677000	-0.207498000	-2.082956000
C	0.099832000	-1.836427000	0.190161000
C	0.521989000	-2.816041000	-0.710435000
H	1.005044000	-2.516057000	-1.633100000
C	0.346818000	-4.173217000	-0.434384000
H	0.682868000	-4.918146000	-1.149634000
C	-0.259726000	-4.559487000	0.755392000
H	-0.398588000	-5.613406000	0.983296000
C	-0.706868000	-3.602656000	1.667363000
H	-1.187965000	-3.918490000	2.586866000
C	-0.543977000	-2.244689000	1.383711000
O	-0.983992000	-1.248748000	2.183910000
C	-1.572959000	-1.567816000	3.430220000
H	-2.495066000	-2.147985000	3.301010000
H	-1.808611000	-0.610589000	3.896383000
H	-0.875075000	-2.124479000	4.068339000
C	2.561908000	0.400463000	3.119022000
C	3.442782000	-0.496303000	3.948061000
H	2.996285000	-1.488158000	4.081478000
H	4.414744000	-0.642710000	3.460646000
H	3.626189000	-0.070532000	4.939925000
H	2.872217000	2.157241000	4.324456000
H	2.040212000	-1.144132000	1.726857000
H	1.446105000	3.621296000	2.892854000
C	-4.509354000	-1.228962000	0.532815000
H	-3.775890000	-2.002407000	0.794264000
H	-4.309699000	-0.387603000	1.210299000

C	-2.700209000	-0.786751000	-1.233061000
H	-2.254998000	-1.737454000	-0.931557000
C	-4.205970000	-0.811321000	-0.919131000
H	-4.690046000	-1.532034000	-1.598123000
C	3.462292000	-0.403320000	-1.055461000
C	4.026050000	0.737996000	-0.449165000
C	4.069555000	-1.650155000	-0.834074000
C	5.125686000	0.624977000	0.410465000
C	5.157848000	-1.775741000	0.038518000
C	5.669742000	-0.632875000	0.647236000
H	5.556259000	1.502814000	0.878001000
H	5.615991000	-2.739444000	0.228078000
H	6.526079000	-0.725276000	1.311097000
O	3.545857000	-2.694972000	-1.533511000
O	3.442811000	1.903411000	-0.787912000
C	4.148969000	-3.963604000	-1.390051000
H	4.059031000	-4.342355000	-0.363477000
H	3.609358000	-4.629575000	-2.066271000
H	5.208463000	-3.941166000	-1.677103000
C	3.928457000	3.112578000	-0.236066000
H	3.262897000	3.888058000	-0.615416000
H	3.878279000	3.099544000	0.859037000
H	4.960116000	3.308982000	-0.557707000
H	-2.534372000	-0.654901000	-2.308274000
C	-4.804468000	0.534790000	-1.273670000
F	-4.629024000	0.845998000	-2.566317000
F	-4.179791000	1.534847000	-0.548873000
F	-6.112202000	0.653377000	-1.007676000
C	-5.913433000	-1.785863000	0.786339000
H	-6.668409000	-1.023977000	0.568173000
H	-6.100805000	-2.615405000	0.089271000
C	-6.088271000	-2.279507000	2.220998000
H	-5.932896000	-1.467109000	2.941542000
H	-7.095402000	-2.678742000	2.384723000
H	-5.372898000	-3.077368000	2.458187000

82

A3,21(Coor3)

Pd	2.245417000	0.390239000	0.228898000
P	-0.139645000	0.395182000	0.042209000
C	-0.885897000	-0.949016000	1.052041000
C	-0.262708000	-2.202784000	1.165034000
S	1.227494000	-2.602562000	0.241265000
O	2.262654000	-1.677620000	0.879855000
O	0.939409000	-2.211448000	-1.150482000
O	1.523603000	-4.011502000	0.511056000

C	-0.781145000	-3.164275000	2.026768000
C	-1.916805000	-2.888909000	2.784948000
C	-2.025151000	-0.695175000	1.820693000
C	-0.702705000	0.392885000	-1.711901000
C	0.308873000	0.727190000	-2.626110000
H	1.325739000	0.841884000	-2.261739000
C	0.047981000	0.889039000	-3.981927000
H	0.857272000	1.141141000	-4.661763000
C	-1.248069000	0.708881000	-4.452944000
H	-1.475180000	0.830291000	-5.509085000
C	-2.252674000	0.340793000	-3.565065000
H	-3.259162000	0.160929000	-3.932709000
C	-2.011605000	0.163084000	-2.194938000
C	-0.828385000	1.932336000	0.765708000
C	-1.546388000	2.863800000	0.013889000
H	-1.798940000	2.633349000	-1.014671000
C	-1.960083000	4.075978000	0.569507000
H	-2.517014000	4.786007000	-0.034962000
C	-1.651898000	4.365800000	1.893482000
H	-1.970487000	5.305314000	2.338248000
C	-0.922365000	3.461153000	2.665740000
H	-0.680350000	3.704591000	3.694693000
C	-0.499558000	2.254897000	2.103352000
O	0.239244000	1.331524000	2.762688000
C	0.600796000	1.569342000	4.108891000
H	1.217628000	2.472330000	4.204517000
H	1.179579000	0.697711000	4.416580000
H	-0.285251000	1.663261000	4.749549000
C	-2.558705000	-1.652084000	2.690485000
C	2.203649000	2.432861000	-0.133419000
H	1.301154000	2.661102000	-0.708733000
H	2.047462000	2.833524000	0.878469000
C	-3.806239000	-1.354984000	3.479785000
H	-3.805686000	-0.327997000	3.861951000
H	-4.698371000	-1.465496000	2.850717000
H	-3.912093000	-2.034358000	4.331942000
H	-2.306021000	-3.646618000	3.462233000
H	-2.514674000	0.271371000	1.740909000
H	-0.271221000	-4.120291000	2.096744000
C	4.490103000	0.216185000	-0.133840000
H	4.695953000	1.029324000	-0.820010000
C	4.268752000	0.452036000	1.196968000
H	4.302778000	1.461842000	1.595014000
C	-3.168454000	-0.343614000	-1.397733000
C	-3.268433000	-1.728118000	-1.152341000

C	-4.259903000	0.476230000	-1.067329000
C	-4.402370000	-2.267525000	-0.532290000
C	-5.391340000	-0.051361000	-0.432657000
C	-5.444183000	-1.418618000	-0.175247000
H	-4.475751000	-3.331626000	-0.340480000
H	-6.227253000	0.582007000	-0.159755000
H	-6.328536000	-1.833768000	0.302410000
O	-4.143051000	1.787147000	-1.420596000
O	-2.219011000	-2.455995000	-1.579165000
C	-5.226298000	2.650189000	-1.146920000
H	-5.415263000	2.735307000	-0.068732000
H	-4.936889000	3.627474000	-1.538181000
H	-6.143759000	2.318554000	-1.650802000
C	-2.216846000	-3.856293000	-1.378240000
H	-1.249182000	-4.197956000	-1.745664000
H	-2.305994000	-4.106559000	-0.314437000
H	-3.027237000	-4.339174000	-1.941014000
C	3.388845000	3.118614000	-0.801303000
H	4.309993000	2.989716000	-0.216376000
H	3.578974000	2.681840000	-1.791864000
C	3.138193000	4.622578000	-0.970254000
H	2.965925000	5.104265000	-0.000251000
H	3.994806000	5.116304000	-1.444963000
H	2.254906000	4.807065000	-1.592817000
H	4.243362000	-0.371713000	1.904699000
C	4.901797000	-1.119638000	-0.699032000
F	6.142713000	-0.962920000	-1.229462000
F	4.101602000	-1.518881000	-1.695772000
F	4.978673000	-2.089799000	0.210569000

82

A3,21(Coor4)

Pd	-2.415206000	0.276715000	-0.029006000
P	0.094533000	-0.210624000	0.072303000
C	1.015291000	1.317216000	0.525297000
C	0.583948000	2.563581000	0.040330000
S	-0.785381000	2.709678000	-1.114838000
O	-2.006077000	2.337127000	-0.254321000
O	-0.554925000	1.694086000	-2.157362000
O	-0.853089000	4.114837000	-1.518001000
C	1.192513000	3.737512000	0.475993000
C	2.227007000	3.684842000	1.406126000
C	2.056889000	1.291243000	1.456818000
C	0.793759000	-1.031536000	-1.423752000
C	-0.194245000	-1.572758000	-2.261200000
H	-1.237579000	-1.364130000	-2.050551000

C	0.124419000	-2.342189000	-3.375116000
H	-0.669551000	-2.740238000	-4.001027000
C	1.460480000	-2.578602000	-3.679506000
H	1.734151000	-3.178528000	-4.543812000
C	2.451977000	-2.014086000	-2.884141000
H	3.497995000	-2.161134000	-3.139289000
C	2.152022000	-1.229252000	-1.761482000
C	0.447566000	-1.393989000	1.434931000
C	0.937787000	-2.682478000	1.202848000
H	1.244719000	-2.958008000	0.200185000
C	1.061180000	-3.610967000	2.238604000
H	1.441813000	-4.606507000	2.029000000
C	0.700652000	-3.251572000	3.531758000
H	0.798741000	-3.962709000	4.348121000
C	0.200001000	-1.975849000	3.796249000
H	-0.084188000	-1.708695000	4.808560000
C	0.057110000	-1.057736000	2.753760000
O	-0.465199000	0.184803000	2.909984000
C	-0.808146000	0.632525000	4.207023000
H	-1.597428000	0.012889000	4.653003000
H	-1.176099000	1.651130000	4.079042000
H	0.066346000	0.638842000	4.869845000
C	2.676523000	2.460830000	1.908909000
C	3.816737000	2.395031000	2.890167000
H	3.691493000	1.568858000	3.598484000
H	4.767928000	2.231621000	2.368135000
H	3.906938000	3.324314000	3.462622000
H	2.687694000	4.609391000	1.748501000
H	2.400465000	0.334554000	1.841301000
H	0.832472000	4.683445000	0.083370000
C	-4.354571000	0.969771000	-0.258613000
H	-5.035469000	0.129782000	-0.408047000
H	-4.219544000	1.515833000	-1.198583000
C	-2.885396000	-1.837607000	0.119693000
H	-1.933226000	-2.358878000	0.076524000
C	-3.263088000	-1.193649000	1.291971000
H	-2.586376000	-1.177895000	2.140386000
C	3.322418000	-0.598808000	-1.082621000
C	3.674277000	0.722211000	-1.423412000
C	4.196704000	-1.338265000	-0.269719000
C	4.843964000	1.306086000	-0.921338000
C	5.360697000	-0.758154000	0.250179000
C	5.666991000	0.557386000	-0.086887000
H	5.112473000	2.323384000	-1.181393000
H	6.030573000	-1.321290000	0.889451000

H	6.578793000	1.004989000	0.301538000
O	3.842293000	-2.634685000	-0.049188000
O	2.820390000	1.339110000	-2.265613000
C	4.701099000	-3.436658000	0.733077000
H	4.787935000	-3.057515000	1.759859000
H	4.246336000	-4.428829000	0.755758000
H	5.702261000	-3.509675000	0.287887000
C	3.089827000	2.667086000	-2.671112000
H	2.250172000	2.952127000	-3.305621000
H	3.138080000	3.346475000	-1.811569000
H	4.026567000	2.727195000	-3.241653000
C	-4.786026000	1.883162000	0.870625000
H	-4.891687000	1.317501000	1.807662000
H	-4.016614000	2.644390000	1.036778000
C	-6.120679000	2.572619000	0.552372000
H	-6.428706000	3.226798000	1.377003000
H	-6.035912000	3.188204000	-0.350404000
H	-6.920651000	1.840814000	0.385567000
H	-4.305550000	-0.974432000	1.495816000
C	-3.870815000	-2.382693000	-0.879679000
F	-3.731074000	-3.722392000	-0.953693000
F	-5.153161000	-2.136177000	-0.553683000
F	-3.671117000	-1.898784000	-2.121311000

82

A3,21(TS2)

Pd	-2.370274000	0.241428000	0.019524000
P	-0.004298000	-0.141739000	0.043432000
C	0.859148000	1.321669000	0.758156000
C	0.454142000	2.628861000	0.438729000
S	-0.839969000	2.959645000	-0.764470000
O	-2.079301000	2.348540000	-0.097903000
O	-0.467367000	2.229293000	-1.985080000
O	-0.973303000	4.416221000	-0.847157000
C	1.045252000	3.718617000	1.069813000
C	2.042176000	3.522734000	2.023546000
C	1.864202000	1.150811000	1.712850000
C	0.710084000	-0.628236000	-1.583814000
C	-0.266058000	-0.920049000	-2.548040000
H	-1.309276000	-0.750468000	-2.304026000
C	0.067069000	-1.399505000	-3.809093000
H	-0.717329000	-1.611791000	-4.530154000
C	1.405218000	-1.594031000	-4.132053000
H	1.689477000	-1.973138000	-5.110542000
C	2.386903000	-1.269466000	-3.202233000
H	3.435881000	-1.378016000	-3.463976000

C	2.074855000	-0.768874000	-1.930323000
C	0.394969000	-1.497935000	1.213253000
C	1.004114000	-2.684636000	0.804672000
H	1.339727000	-2.782111000	-0.220697000
C	1.195918000	-3.743182000	1.694294000
H	1.669857000	-4.657899000	1.350675000
C	0.770248000	-3.621375000	3.011515000
H	0.914589000	-4.438451000	3.713960000
C	0.141641000	-2.453517000	3.446112000
H	-0.195481000	-2.374203000	4.474032000
C	-0.057523000	-1.400253000	2.550824000
O	-0.693057000	-0.246754000	2.873689000
C	-1.144169000	-0.054990000	4.199857000
H	-1.894681000	-0.805039000	4.482083000
H	-1.597207000	0.937052000	4.216308000
H	-0.311558000	-0.087322000	4.913971000
C	2.470232000	2.236324000	2.355843000
C	3.567504000	2.007063000	3.361338000
H	3.297217000	1.224524000	4.079887000
H	4.488530000	1.682262000	2.861694000
H	3.793269000	2.919132000	3.923188000
H	2.489284000	4.383466000	2.516976000
H	2.189930000	0.145796000	1.966863000
H	0.700986000	4.714137000	0.807344000
C	-2.855201000	-1.747969000	0.413693000
H	-2.279142000	-2.062320000	1.280826000
C	-4.080948000	-1.058422000	0.602243000
H	-4.395053000	-0.810650000	1.611488000
C	3.245105000	-0.336011000	-1.108730000
C	3.619132000	1.022985000	-1.134573000
C	4.115256000	-1.255933000	-0.500169000
C	4.788239000	1.460434000	-0.499557000
C	5.276253000	-0.826304000	0.155098000
C	5.594419000	0.528847000	0.145727000
H	5.069884000	2.506897000	-0.516431000
H	5.935672000	-1.532197000	0.646200000
H	6.503894000	0.863153000	0.639380000
O	3.765263000	-2.567393000	-0.616008000
O	2.788078000	1.825890000	-1.828232000
C	4.627131000	-3.540285000	-0.065303000
H	4.704471000	-3.443389000	1.025636000
H	4.181107000	-4.506565000	-0.309160000
H	5.631091000	-3.486427000	-0.506676000
C	3.097967000	3.199519000	-1.956212000
H	2.274079000	3.627799000	-2.527672000

H	3.151483000	3.690938000	-0.977470000
H	4.044081000	3.344843000	-2.495089000
C	-4.453868000	1.136433000	0.002917000
H	-4.108510000	1.620200000	-0.913723000
H	-4.213947000	1.760446000	0.866660000
C	-5.933441000	0.824120000	-0.089423000
H	-6.143545000	0.196362000	-0.965811000
H	-6.294316000	0.278094000	0.792108000
C	-6.727865000	2.131557000	-0.216565000
H	-6.426882000	2.695449000	-1.106275000
H	-7.801302000	1.922245000	-0.294477000
H	-6.570806000	2.776920000	0.655036000
H	-4.881021000	-1.260431000	-0.098476000
C	-2.800773000	-2.750813000	-0.700518000
F	-3.508404000	-3.859086000	-0.373069000
F	-1.550366000	-3.163791000	-0.966940000
F	-3.333937000	-2.281430000	-1.848817000

82

A3,21(P2)

Pd	-1.902723000	1.136283000	-0.541697000
P	0.048901000	0.023038000	-0.143900000
C	1.129759000	1.150959000	0.830573000
C	1.191725000	2.529773000	0.559271000
S	0.337563000	3.294499000	-0.834649000
O	-1.137993000	3.101214000	-0.471469000
O	0.713920000	2.502946000	-2.016407000
O	0.699323000	4.713377000	-0.814809000
C	1.933424000	3.366111000	1.387457000
C	2.613928000	2.848393000	2.487871000
C	1.826171000	0.652632000	1.934727000
C	0.876037000	-0.641613000	-1.644321000
C	0.064243000	-0.649723000	-2.787702000
H	-0.917152000	-0.192486000	-2.740304000
C	0.492428000	-1.214441000	-3.983345000
H	-0.162780000	-1.200743000	-4.849698000
C	1.758831000	-1.783396000	-4.054624000
H	2.112071000	-2.234024000	-4.978725000
C	2.589066000	-1.747156000	-2.939440000
H	3.595338000	-2.152282000	-3.000983000
C	2.185665000	-1.171061000	-1.727017000
C	-0.268496000	-1.405791000	0.953657000
C	-0.013867000	-2.718314000	0.551910000
H	0.467145000	-2.896522000	-0.402299000
C	-0.358065000	-3.800024000	1.363004000
H	-0.152518000	-4.812687000	1.029030000

C	-0.968116000	-3.572219000	2.591221000
H	-1.237866000	-4.406955000	3.233339000
C	-1.253242000	-2.272447000	3.010793000
H	-1.740953000	-2.110669000	3.966040000
C	-0.921640000	-1.191090000	2.191221000
O	-1.203493000	0.098408000	2.495328000
C	-1.740747000	0.406802000	3.767148000
H	-2.732691000	-0.042173000	3.906069000
H	-1.827400000	1.493660000	3.794014000
H	-1.073312000	0.072438000	4.571239000
C	2.576184000	1.483226000	2.775094000
C	3.331628000	0.903086000	3.941333000
H	2.704018000	0.223935000	4.530167000
H	4.195578000	0.324091000	3.592832000
H	3.699652000	1.687938000	4.609843000
H	3.180060000	3.518632000	3.131583000
H	1.791106000	-0.411967000	2.150148000
H	1.955449000	4.426835000	1.157426000
C	-5.159556000	0.747122000	0.840307000
H	-5.714641000	1.694340000	0.866758000
H	-4.469105000	0.769574000	1.694701000
C	-3.249798000	-0.376889000	-0.477117000
H	-3.159097000	-0.953830000	0.446562000
C	-4.340789000	0.677056000	-0.452442000
H	-4.990526000	0.602564000	-1.333185000
C	3.238054000	-1.090075000	-0.670354000
C	3.986561000	0.099123000	-0.556231000
C	3.653947000	-2.215759000	0.059593000
C	5.077699000	0.181031000	0.317799000
C	4.732892000	-2.138685000	0.949635000
C	5.429445000	-0.938648000	1.063870000
H	5.647603000	1.098373000	0.409017000
H	5.044252000	-3.000306000	1.528572000
H	6.278436000	-0.881522000	1.741045000
O	2.960723000	-3.363496000	-0.177481000
O	3.579497000	1.102914000	-1.357019000
C	3.357672000	-4.535506000	0.502268000
H	3.233250000	-4.435776000	1.588565000
H	2.702829000	-5.329230000	0.137528000
H	4.399974000	-4.798246000	0.278458000
C	4.279135000	2.332228000	-1.340420000
H	3.744918000	2.976094000	-2.039275000
H	4.260494000	2.788873000	-0.343766000
H	5.319348000	2.201724000	-1.668135000
C	-6.139047000	-0.418758000	0.988713000

H	-6.834142000	-0.413170000	0.137807000
H	-5.594149000	-1.369420000	0.923418000
C	-6.927886000	-0.361834000	2.294843000
H	-7.629481000	-1.199804000	2.372035000
H	-6.261194000	-0.405354000	3.165174000
H	-7.507870000	0.566103000	2.369743000
H	-3.875399000	1.708262000	-0.594584000
C	-3.346992000	-1.320237000	-1.636670000
F	-4.515533000	-2.013583000	-1.611289000
F	-3.319724000	-0.679047000	-2.829186000
F	-2.361836000	-2.236147000	-1.645093000

71

A1,E12(Coor5)

Pd	-0.479765000	-1.406842000	-0.619413000
P	0.872476000	0.379935000	0.019545000
C	2.675699000	0.043673000	-0.159530000
C	3.224357000	-1.214972000	0.137282000
S	2.187281000	-2.604856000	0.614357000
O	1.259028000	-2.719162000	-0.596629000
O	1.458628000	-2.182591000	1.822395000
O	3.064476000	-3.772774000	0.715571000
C	4.594309000	-1.438988000	0.020934000
C	5.437460000	-0.414778000	-0.401502000
C	3.534703000	1.057017000	-0.601182000
C	0.622753000	0.955212000	1.736482000
C	-0.249725000	0.221389000	2.545411000
H	-0.748332000	-0.641679000	2.121039000
C	-0.459025000	0.557472000	3.880184000
H	-1.144129000	-0.028970000	4.483980000
C	0.228617000	1.634866000	4.425901000
H	0.082363000	1.907887000	5.468035000
C	1.121350000	2.374367000	3.651272000
H	1.655788000	3.205482000	4.097939000
C	1.327667000	2.038210000	2.310545000
O	2.178524000	2.712351000	1.497482000
C	2.974685000	3.742195000	2.048753000
H	2.360150000	4.574950000	2.414917000
H	3.609456000	4.095771000	1.234553000
H	3.605551000	3.367131000	2.864145000
C	0.546957000	1.810863000	-1.074933000
C	-0.034700000	2.992963000	-0.611787000
H	-0.236485000	3.102348000	0.448774000
C	-0.360183000	4.030947000	-1.484605000
H	-0.808469000	4.942675000	-1.100615000
C	-0.103156000	3.887189000	-2.843995000

H	-0.345375000	4.690503000	-3.535223000
C	0.459011000	2.710844000	-3.339545000
H	0.643760000	2.609480000	-4.403535000
C	0.771132000	1.668170000	-2.462988000
O	1.287991000	0.480283000	-2.852408000
C	1.572717000	0.265290000	-4.220963000
H	0.663716000	0.326573000	-4.833088000
H	1.985373000	-0.742435000	-4.280362000
H	2.313229000	0.985260000	-4.591866000
C	4.904066000	0.831866000	-0.720459000
C	-2.038322000	-0.011540000	-0.631357000
H	-1.748633000	0.824490000	0.012906000
H	6.505888000	-0.594261000	-0.490521000
H	3.126979000	2.031266000	-0.848375000
H	4.972618000	-2.429383000	0.253478000
C	-2.182123000	-2.837275000	-1.647362000
C	-0.981668000	-3.449575000	-1.762988000
H	-0.345208000	-3.308547000	-2.632249000
H	-2.843123000	-3.074865000	-0.821036000
H	5.552305000	1.634812000	-1.062753000
H	-0.633198000	-4.166838000	-1.027631000
H	-2.571523000	-2.200629000	-2.437179000
C	-3.494344000	-0.355369000	-0.282330000
H	-3.878501000	-1.149667000	-0.929946000
C	-4.370055000	0.900892000	-0.513566000
H	-4.094956000	1.291726000	-1.502205000
H	-4.077554000	1.675219000	0.207406000
H	-2.009502000	0.361346000	-1.662253000
C	-3.649654000	-0.881834000	1.133893000
F	-4.897495000	-1.346751000	1.367998000
F	-2.816804000	-1.915617000	1.399620000
F	-3.402053000	0.066624000	2.060375000
C	-5.890341000	0.709167000	-0.478463000
H	-6.213969000	0.422358000	0.526795000
H	-6.169384000	-0.123648000	-1.139420000
C	-6.631216000	1.973571000	-0.910352000
H	-7.716904000	1.828334000	-0.877069000
H	-6.389491000	2.818652000	-0.253938000
H	-6.364673000	2.263922000	-1.934313000

71

A1,E21(Coor5)

Pd	0.606189000	-1.494166000	-0.306460000
P	-0.789177000	0.400273000	0.045796000
C	-2.570919000	-0.043259000	0.204439000
C	-3.138467000	-1.102943000	-0.523891000

S	-2.166142000	-2.135833000	-1.632506000
O	-1.113227000	-2.727895000	-0.689635000
O	-1.545888000	-1.224131000	-2.610963000
O	-3.062995000	-3.175909000	-2.138115000
C	-4.490523000	-1.412393000	-0.388735000
C	-5.294318000	-0.678217000	0.479118000
C	-3.389880000	0.673659000	1.084592000
C	-0.669672000	1.617629000	-1.314817000
C	0.147941000	1.304652000	-2.404733000
H	0.667034000	0.352095000	-2.400901000
C	0.266029000	2.158444000	-3.498109000
H	0.903577000	1.887463000	-4.334048000
C	-0.460866000	3.343593000	-3.514226000
H	-0.387223000	4.020245000	-4.361870000
C	-1.302748000	3.674904000	-2.454175000
H	-1.870557000	4.598127000	-2.491573000
C	-1.417736000	2.816207000	-1.356525000
O	-2.217050000	3.071449000	-0.293115000
C	-3.064378000	4.202841000	-0.332260000
H	-2.488626000	5.137122000	-0.356020000
H	-3.651222000	4.165848000	0.586838000
H	-3.739808000	4.166146000	-1.195841000
C	-0.364017000	1.297965000	1.579997000
C	0.191025000	2.580261000	1.578505000
H	0.305887000	3.107695000	0.636971000
C	0.604085000	3.187796000	2.763381000
H	1.033251000	4.185215000	2.741295000
C	0.462540000	2.505119000	3.967772000
H	0.778512000	2.968771000	4.898770000
C	-0.072307000	1.218169000	3.997916000
H	-0.161631000	0.693812000	4.942996000
C	-0.473192000	0.607110000	2.807194000
O	-0.983070000	-0.640874000	2.724778000
C	-1.031002000	-1.445734000	3.888538000
H	-0.030485000	-1.580208000	4.315574000
H	-1.422871000	-2.409560000	3.562017000
H	-1.703065000	-1.015646000	4.642037000
C	-4.740279000	0.362218000	1.220865000
C	2.292141000	-0.359699000	0.170489000
H	1.924364000	0.647782000	0.386277000
H	-6.347983000	-0.925226000	0.580591000
H	-2.963124000	1.484022000	1.665946000
H	-4.887036000	-2.241070000	-0.966593000
C	1.649634000	-3.533878000	-0.225766000
C	1.665248000	-3.112345000	-1.526742000

H	0.841831000	-3.343605000	-2.196627000
H	2.526484000	-3.448669000	0.405813000
H	0.809195000	-4.111774000	0.150380000
H	-5.356930000	0.934772000	1.909278000
H	2.554638000	-2.673633000	-1.967660000
C	3.336798000	-0.271023000	-0.946498000
H	3.852365000	-1.232398000	-1.058140000
H	2.815239000	-0.087803000	-1.893777000
C	4.387040000	0.834765000	-0.756352000
H	4.960663000	0.652260000	0.160113000
H	3.875060000	1.797089000	-0.610886000
C	2.885864000	-0.835059000	1.479499000
F	3.785116000	-1.841599000	1.325053000
F	1.956998000	-1.290651000	2.353555000
F	3.549843000	0.153006000	2.127881000
C	5.347416000	0.945165000	-1.942186000
H	5.851013000	-0.020549000	-2.093000000
H	4.772470000	1.134978000	-2.860071000
C	6.394770000	2.041808000	-1.760190000
H	7.008520000	1.858985000	-0.869760000
H	7.068255000	2.099735000	-2.622980000
H	5.922282000	3.024423000	-1.639441000

71

A1,E12(Coor6)

Pd	1.006284000	-0.350544000	-0.542785000
P	-1.459496000	0.112208000	-0.228843000
C	-2.075213000	-0.522863000	1.389095000
C	-1.564473000	-1.714506000	1.932246000
S	-0.282949000	-2.670533000	1.102734000
O	0.906585000	-1.693462000	1.076283000
O	-0.775212000	-2.933222000	-0.261258000
O	0.014984000	-3.816146000	1.961389000
C	-2.018346000	-2.189615000	3.161040000
C	-2.979980000	-1.477547000	3.873045000
C	-3.028040000	0.188077000	2.126929000
C	-2.588790000	-0.559804000	-1.507941000
C	-2.021235000	-1.362065000	-2.503957000
H	-0.959206000	-1.576738000	-2.450762000
C	-2.793959000	-1.936610000	-3.510452000
H	-2.327018000	-2.561864000	-4.265464000
C	-4.167427000	-1.721162000	-3.517313000
H	-4.788588000	-2.164511000	-4.291580000
C	-4.769237000	-0.949815000	-2.524531000
H	-5.844121000	-0.806491000	-2.538305000
C	-3.990440000	-0.376198000	-1.514284000

O	-4.510740000	0.375561000	-0.513506000
C	-5.914623000	0.516962000	-0.427211000
H	-6.321319000	1.048163000	-1.297706000
H	-6.099252000	1.107514000	0.471593000
H	-6.409444000	-0.457347000	-0.329571000
C	-1.769571000	1.918969000	-0.185161000
C	-2.502614000	2.598246000	-1.161579000
H	-3.007623000	2.029361000	-1.935597000
C	-2.591539000	3.990772000	-1.163645000
H	-3.170806000	4.496312000	-1.931005000
C	-1.937808000	4.720851000	-0.176037000
H	-2.004514000	5.805795000	-0.162438000
C	-1.179823000	4.073751000	0.800054000
H	-0.663831000	4.655544000	1.556268000
C	-1.082668000	2.679677000	0.789308000
O	-0.342093000	1.961368000	1.662897000
C	0.406133000	2.634491000	2.659214000
H	1.140530000	3.315541000	2.213082000
H	0.928034000	1.852821000	3.210821000
H	-0.251891000	3.190743000	3.338681000
C	-3.479087000	-0.283588000	3.357550000
H	-3.330802000	-1.851635000	4.831456000
H	-3.419067000	1.118672000	1.729018000
H	-1.596571000	-3.113761000	3.543055000
H	-4.222149000	0.284572000	3.911898000
C	2.984006000	-0.963960000	-0.598250000
C	1.289186000	0.409382000	-2.566067000
C	1.299945000	1.478981000	-1.687585000
C	4.147137000	0.023702000	-0.588820000
H	4.035919000	0.777731000	-1.380469000
C	5.468099000	-0.740361000	-0.849505000
H	5.304106000	-1.338402000	-1.755818000
H	5.620032000	-1.461984000	-0.036780000
C	4.198045000	0.799004000	0.718587000
F	5.092745000	1.813223000	0.674722000
F	3.010267000	1.374597000	1.025007000
F	4.533269000	0.016964000	1.759726000
H	2.227328000	1.922193000	-1.340857000
H	0.408789000	2.080921000	-1.536689000
H	0.385161000	0.155338000	-3.113462000
H	2.211743000	-0.010621000	-2.956170000
H	3.039029000	-1.596448000	-1.491860000
H	3.008566000	-1.609572000	0.283847000
C	6.737412000	0.095212000	-1.048013000
H	6.548505000	0.877247000	-1.797185000

H	6.999275000	0.616222000	-0.121661000
C	7.916087000	-0.768222000	-1.495097000
H	7.705889000	-1.270447000	-2.447566000
H	8.821487000	-0.165410000	-1.628523000
H	8.140572000	-1.545447000	-0.754510000

71

A1,E21(Coor6)

Pd	1.050671000	-0.559870000	-0.165560000
P	-1.375783000	-0.048452000	-0.135220000
C	-1.793982000	1.340011000	1.001014000
C	-1.083775000	1.523494000	2.199856000
S	0.251282000	0.420636000	2.691265000
O	1.290202000	0.619442000	1.571719000
O	-0.306954000	-0.941882000	2.647750000
O	0.770564000	0.920971000	3.963050000
C	-1.383249000	2.585560000	3.050696000
C	-2.388486000	3.488025000	2.713104000
C	-2.788884000	2.266456000	0.669927000
C	-2.453606000	-1.458863000	0.313120000
C	-1.829145000	-2.661950000	0.661223000
H	-0.744705000	-2.703482000	0.665989000
C	-2.563901000	-3.780787000	1.045446000
H	-2.053078000	-4.699438000	1.317169000
C	-3.950665000	-3.695540000	1.100493000
H	-4.540936000	-4.556247000	1.404751000
C	-4.602867000	-2.506021000	0.780324000
H	-5.684585000	-2.457475000	0.840466000
C	-3.862734000	-1.384763000	0.392521000
O	-4.427730000	-0.196293000	0.067405000
C	-5.823890000	-0.040226000	0.228179000
H	-6.384412000	-0.703834000	-0.443067000
H	-6.039185000	0.997465000	-0.031745000
H	-6.129584000	-0.225421000	1.265355000
C	-1.928936000	0.498004000	-1.794542000
C	-2.799391000	-0.251148000	-2.590321000
H	-3.246609000	-1.151729000	-2.181641000
C	-3.104425000	0.136380000	-3.895245000
H	-3.786885000	-0.460742000	-4.493109000
C	-2.533728000	1.292276000	-4.417905000
H	-2.768661000	1.609820000	-5.430563000
C	-1.646353000	2.052329000	-3.655465000
H	-1.200016000	2.945090000	-4.080048000
C	-1.331857000	1.651230000	-2.354263000
O	-0.451451000	2.295967000	-1.552057000
C	0.166834000	3.481508000	-2.016020000

H	0.776747000	3.292539000	-2.908693000
H	0.809201000	3.816234000	-1.200968000
H	-0.577535000	4.256597000	-2.237731000
C	-3.086491000	3.330701000	1.517757000
H	-2.619553000	4.314997000	3.379567000
H	-3.334962000	2.148377000	-0.260161000
H	-0.810551000	2.687896000	3.966851000
H	-3.866610000	4.036076000	1.242015000
C	3.120676000	-0.776683000	0.105582000
H	3.110952000	-0.756013000	1.199749000
C	1.255380000	-0.897234000	-2.316534000
H	2.310583000	-0.823931000	-2.549242000
C	0.741881000	-2.050278000	-1.752285000
H	0.598821000	-0.166024000	-2.780507000
H	-0.325142000	-2.253115000	-1.787017000
H	1.382469000	-2.893268000	-1.519144000
C	3.720248000	-2.093407000	-0.289797000
C	3.830968000	0.458217000	-0.440215000
H	3.912158000	0.402845000	-1.534180000
H	3.192282000	1.321908000	-0.214784000
C	5.223378000	0.740698000	0.150997000
H	5.916980000	-0.067166000	-0.109655000
H	5.151846000	0.747901000	1.247452000
F	4.953648000	-2.278662000	0.239708000
F	2.978678000	-3.145389000	0.134584000
F	3.868946000	-2.241076000	-1.633346000
C	5.793780000	2.075805000	-0.330369000
H	5.102925000	2.885350000	-0.053999000
H	5.840818000	2.076030000	-1.429453000
C	7.180126000	2.373415000	0.237501000
H	7.563716000	3.334985000	-0.123310000
H	7.158019000	2.413556000	1.333285000
H	7.900920000	1.598109000	-0.049923000

71

A1,E12(TS3)

Pd	0.801543000	-0.225813000	-0.817812000
P	-1.407389000	0.114949000	-0.166287000
C	-1.791465000	-0.620919000	1.476555000
C	-1.199424000	-1.826349000	1.893667000
S	-0.020418000	-2.736146000	0.872883000
O	1.116738000	-1.723529000	0.697645000
O	-0.706838000	-3.006772000	-0.403062000
O	0.420699000	-3.884860000	1.666527000
C	-1.495733000	-2.362600000	3.145335000
C	-2.373502000	-1.703634000	4.002150000

C	-2.658811000	0.036491000	2.356276000
C	-2.634029000	-0.548994000	-1.351035000
C	-2.150382000	-1.244156000	-2.463891000
H	-1.076716000	-1.368577000	-2.564294000
C	-3.010244000	-1.811762000	-3.400714000
H	-2.607778000	-2.353232000	-4.251455000
C	-4.383774000	-1.698022000	-3.216951000
H	-5.071405000	-2.139395000	-3.934093000
C	-4.898911000	-1.030291000	-2.107237000
H	-5.973248000	-0.962238000	-1.976123000
C	-4.032416000	-0.461197000	-1.168561000
O	-4.460637000	0.199894000	-0.065566000
C	-5.846697000	0.240816000	0.209294000
H	-6.395571000	0.789715000	-0.567073000
H	-5.945843000	0.767949000	1.159532000
H	-6.264622000	-0.768648000	0.308933000
C	-1.755805000	1.905122000	-0.010474000
C	-2.635235000	2.587078000	-0.853471000
H	-3.215393000	2.027264000	-1.579811000
C	-2.772333000	3.973828000	-0.786035000
H	-3.464554000	4.483484000	-1.450118000
C	-2.017916000	4.692835000	0.135709000
H	-2.118694000	5.773272000	0.201626000
C	-1.118133000	4.041329000	0.978996000
H	-0.528808000	4.615144000	1.686041000
C	-0.977430000	2.653071000	0.901779000
O	-0.115997000	1.932534000	1.652931000
C	0.763884000	2.604649000	2.535542000
H	1.394559000	3.320524000	1.995684000
H	1.391716000	1.827439000	2.970044000
H	0.209820000	3.121242000	3.329523000
C	-2.950505000	-0.498768000	3.608447000
H	-2.598288000	-2.127649000	4.977549000
H	-3.109149000	0.976236000	2.054085000
H	-1.016221000	-3.293887000	3.429173000
H	-3.628715000	0.029012000	4.274530000
C	2.995513000	-0.664821000	-1.328820000
C	1.910354000	0.606853000	-2.662481000
C	0.655665000	1.189024000	-2.306749000
C	4.179477000	0.168911000	-0.841722000
H	4.547576000	0.815555000	-1.650781000
C	5.325148000	-0.785872000	-0.423423000
H	5.268288000	-1.669200000	-1.073234000
H	5.129925000	-1.153119000	0.592058000
C	3.808121000	1.116680000	0.286128000

F	4.875059000	1.816068000	0.732123000
F	2.900319000	2.041256000	-0.113499000
F	3.285540000	0.478337000	1.345553000
H	0.649413000	2.181542000	-1.859541000
H	-0.209686000	0.965418000	-2.928744000
H	1.909758000	-0.105989000	-3.482594000
H	2.791819000	1.237539000	-2.621279000
H	3.278073000	-1.196245000	-2.237448000
H	2.741589000	-1.428737000	-0.584194000
C	6.740702000	-0.208569000	-0.521750000
H	6.908998000	0.156188000	-1.545598000
H	6.839808000	0.660780000	0.135619000
C	7.806949000	-1.243846000	-0.169240000
H	7.754760000	-2.113938000	-0.835187000
H	8.813872000	-0.819721000	-0.252828000
H	7.682835000	-1.606630000	0.858189000
71			
A1,E21(TS3)			
Pd	0.898295000	-0.023750000	-0.442177000
P	-1.408844000	0.063950000	-0.165383000
C	-1.915231000	0.435675000	1.566682000
C	-1.162916000	-0.016617000	2.665103000
S	0.338404000	-0.999132000	2.476697000
O	1.255252000	-0.059755000	1.679523000
O	-0.047355000	-2.175959000	1.680069000
O	0.866325000	-1.220987000	3.823487000
C	-1.556456000	0.289108000	3.966556000
C	-2.695667000	1.056443000	4.194019000
C	-3.049456000	1.218142000	1.813162000
C	-2.239402000	-1.486991000	-0.664685000
C	-1.431908000	-2.548081000	-1.088196000
H	-0.354939000	-2.412515000	-1.092214000
C	-1.977863000	-3.774362000	-1.459042000
H	-1.328160000	-4.583826000	-1.777304000
C	-3.355188000	-3.952724000	-1.392826000
H	-3.798664000	-4.904924000	-1.673057000
C	-4.185464000	-2.921568000	-0.956522000
H	-5.256262000	-3.084307000	-0.902577000
C	-3.635289000	-1.691021000	-0.585405000
O	-4.378684000	-0.642732000	-0.153428000
C	-5.770308000	-0.821471000	0.020800000
H	-6.270927000	-1.038081000	-0.931937000
H	-6.144419000	0.124138000	0.416456000
H	-5.983793000	-1.624418000	0.737162000
C	-2.140539000	1.399267000	-1.183329000

C	-2.975466000	1.148748000	-2.274356000
H	-3.276942000	0.128180000	-2.486927000
C	-3.420247000	2.183344000	-3.097861000
H	-4.071449000	1.965149000	-3.939334000
C	-3.025870000	3.490219000	-2.830035000
H	-3.370766000	4.306391000	-3.459793000
C	-2.176675000	3.769633000	-1.759252000
H	-1.868191000	4.792016000	-1.568827000
C	-1.723369000	2.728434000	-0.945106000
O	-0.870108000	2.891489000	0.091895000
C	-0.401836000	4.188160000	0.407968000
H	0.154880000	4.627733000	-0.429652000
H	0.265240000	4.059053000	1.260955000
H	-1.228553000	4.853482000	0.687538000
C	-3.439497000	1.525906000	3.114249000
H	-2.995914000	1.291216000	5.212043000
H	-3.631960000	1.587926000	0.975823000
H	-0.946959000	-0.080385000	4.784998000
H	-4.326019000	2.133021000	3.280532000
C	3.195380000	-0.251740000	-0.540242000
H	2.923569000	-0.323509000	0.519866000
C	2.142514000	0.339841000	-2.353795000
H	2.817578000	-0.358505000	-2.834403000
C	0.736610000	0.150774000	-2.494088000
H	2.506293000	1.362576000	-2.327907000
H	0.119688000	1.024414000	-2.696543000
H	0.376987000	-0.774457000	-2.942781000
C	3.609904000	-1.647654000	-0.949363000
C	4.275226000	0.812903000	-0.683936000
H	4.639513000	0.862956000	-1.718215000
H	3.811944000	1.783488000	-0.461821000
C	5.472945000	0.624121000	0.260243000
H	5.984784000	-0.318803000	0.033029000
H	5.111839000	0.538452000	1.294160000
F	4.343676000	-2.242328000	0.012512000
F	2.554767000	-2.454361000	-1.187993000
F	4.371467000	-1.665251000	-2.074162000
C	6.471243000	1.778951000	0.159907000
H	5.958232000	2.723567000	0.391674000
H	6.821258000	1.868447000	-0.878697000
C	7.671179000	1.609449000	1.089391000
H	8.369513000	2.449311000	0.997982000
H	7.354818000	1.551085000	2.137807000
H	8.223658000	0.690177000	0.859898000

71

A1,E12(P3)

Pd	0.819289000	0.165487000	0.311038000
P	-1.365743000	0.115374000	-0.177088000
C	-2.274645000	-0.872101000	1.084171000
C	-1.720425000	-2.015436000	1.687637000
S	-0.046410000	-2.606659000	1.336800000
O	0.812563000	-1.416476000	1.758366000
O	0.006879000	-2.828843000	-0.120971000
O	0.173534000	-3.764565000	2.205382000
C	-2.453291000	-2.737108000	2.627987000
C	-3.734161000	-2.328403000	2.990456000
C	-3.557817000	-0.465749000	1.470067000
C	-1.759386000	-0.604268000	-1.810665000
C	-0.702038000	-1.089403000	-2.585628000
H	0.302344000	-1.015280000	-2.183214000
C	-0.921553000	-1.695834000	-3.819506000
H	-0.083147000	-2.071287000	-4.398165000
C	-2.224388000	-1.836514000	-4.284427000
H	-2.416160000	-2.312938000	-5.242589000
C	-3.302170000	-1.383245000	-3.525819000
H	-4.311450000	-1.512915000	-3.900533000
C	-3.079128000	-0.774254000	-2.287177000
O	-4.076806000	-0.312215000	-1.494664000
C	-5.417103000	-0.551419000	-1.877102000
H	-5.668046000	-0.032235000	-2.811197000
H	-6.032400000	-0.153604000	-1.068605000
H	-5.614231000	-1.624620000	-1.989353000
C	-2.098148000	1.788956000	-0.138966000
C	-2.623509000	2.405432000	-1.276966000
H	-2.681963000	1.844722000	-2.204128000
C	-3.063944000	3.728035000	-1.243843000
H	-3.471411000	4.187096000	-2.139883000
C	-2.974693000	4.449471000	-0.057414000
H	-3.317430000	5.480303000	-0.017066000
C	-2.437482000	3.866984000	1.089812000
H	-2.364008000	4.445834000	2.004146000
C	-1.988182000	2.544086000	1.050939000
O	-1.428543000	1.901969000	2.099464000
C	-1.233617000	2.597801000	3.316530000
H	-0.586885000	3.472713000	3.175008000
H	-0.745774000	1.887749000	3.984899000
H	-2.190224000	2.913185000	3.752162000
C	-4.284168000	-1.186140000	2.414884000
H	-4.295133000	-2.896893000	3.727915000
H	-3.990421000	0.423014000	1.022856000

H	-1.988940000	-3.611421000	3.072879000
H	-5.278575000	-0.850754000	2.698983000
C	3.233167000	0.249272000	-1.730997000
C	2.183843000	1.335009000	-2.049114000
C	1.149871000	1.705986000	-0.980509000
C	4.486725000	0.590748000	-0.898627000
H	5.061208000	1.361388000	-1.431459000
C	5.364565000	-0.670241000	-0.731638000
H	5.171005000	-1.323604000	-1.592234000
H	5.029292000	-1.236053000	0.147784000
C	4.161158000	1.213055000	0.445907000
F	5.199319000	1.210430000	1.297162000
F	3.758990000	2.493185000	0.334348000
F	3.156832000	0.537642000	1.092470000
H	1.521711000	2.454602000	-0.269690000
H	0.258568000	2.121999000	-1.456550000
H	1.638057000	0.971992000	-2.928830000
H	2.702984000	2.249288000	-2.380105000
H	3.625139000	-0.111709000	-2.690488000
H	2.739235000	-0.622894000	-1.277142000
C	6.871485000	-0.412612000	-0.651155000
H	7.187204000	0.137731000	-1.549612000
H	7.097329000	0.234989000	0.202716000
C	7.675056000	-1.706501000	-0.539602000
H	7.491997000	-2.364709000	-1.397849000
H	8.750946000	-1.502955000	-0.499184000
H	7.407193000	-2.263089000	0.366489000

71

A1,E21(P3)

Pd	0.898295000	-0.023750000	-0.442177000
P	-1.408844000	0.063950000	-0.165383000
C	-1.915231000	0.435675000	1.566682000
C	-1.162916000	-0.016617000	2.665103000
S	0.338404000	-0.999132000	2.476697000
O	1.255252000	-0.059755000	1.679523000
O	-0.047355000	-2.175959000	1.680069000
O	0.866325000	-1.220987000	3.823487000
C	-1.556456000	0.289108000	3.966556000
C	-2.695667000	1.056443000	4.194019000
C	-3.049456000	1.218142000	1.813162000
C	-2.239402000	-1.486991000	-0.664685000
C	-1.431908000	-2.548081000	-1.088196000
H	-0.354939000	-2.412515000	-1.092214000
C	-1.977863000	-3.774362000	-1.459042000
H	-1.328160000	-4.583826000	-1.777304000

C	-3.355188000	-3.952724000	-1.392826000
H	-3.798664000	-4.904924000	-1.673057000
C	-4.185464000	-2.921568000	-0.956522000
H	-5.256262000	-3.084307000	-0.902577000
C	-3.635289000	-1.691021000	-0.585405000
O	-4.378684000	-0.642732000	-0.153428000
C	-5.770308000	-0.821471000	0.020800000
H	-6.270927000	-1.038081000	-0.931937000
H	-6.144419000	0.124138000	0.416456000
H	-5.983793000	-1.624418000	0.737162000
C	-2.140539000	1.399267000	-1.183329000
C	-2.975466000	1.148748000	-2.274356000
H	-3.276942000	0.128180000	-2.486927000
C	-3.420247000	2.183344000	-3.097861000
H	-4.071449000	1.965149000	-3.939334000
C	-3.025870000	3.490219000	-2.830035000
H	-3.370766000	4.306391000	-3.459793000
C	-2.176675000	3.769633000	-1.759252000
H	-1.868191000	4.792016000	-1.568827000
C	-1.723369000	2.728434000	-0.945106000
O	-0.870108000	2.891489000	0.091895000
C	-0.401836000	4.188160000	0.407968000
H	0.154880000	4.627733000	-0.429652000
H	0.265240000	4.059053000	1.260955000
H	-1.228553000	4.853482000	0.687538000
C	-3.439497000	1.525906000	3.114249000
H	-2.995914000	1.291216000	5.212043000
H	-3.631960000	1.587926000	0.975823000
H	-0.946959000	-0.080385000	4.784998000
H	-4.326019000	2.133021000	3.280532000
C	3.195380000	-0.251740000	-0.540242000
H	2.923569000	-0.323509000	0.519866000
C	2.142514000	0.339841000	-2.353795000
H	2.817578000	-0.358505000	-2.834403000
C	0.736610000	0.150774000	-2.494088000
H	2.506293000	1.362576000	-2.327907000
H	0.119688000	1.024414000	-2.696543000
H	0.376987000	-0.774457000	-2.942781000
C	3.609904000	-1.647654000	-0.949363000
C	4.275226000	0.812903000	-0.683936000
H	4.639513000	0.862956000	-1.718215000
H	3.811944000	1.783488000	-0.461821000
C	5.472945000	0.624121000	0.260243000
H	5.984784000	-0.318803000	0.033029000
H	5.111839000	0.538452000	1.294160000

F	4.343676000	-2.242328000	0.012512000
F	2.554767000	-2.454361000	-1.187993000
F	4.371467000	-1.665251000	-2.074162000
C	6.471243000	1.778951000	0.159907000
H	5.958232000	2.723567000	0.391674000
H	6.821258000	1.868447000	-0.878697000
C	7.671179000	1.609449000	1.089391000
H	8.369513000	2.449311000	0.997982000
H	7.354818000	1.551085000	2.137807000
H	8.223658000	0.690177000	0.859898000

70

A2,E12(Coor5)

Pd	-0.615946000	-0.638457000	-1.168204000
P	0.895806000	-0.128102000	0.561396000
C	2.653087000	-0.166778000	0.032638000
C	3.096516000	-1.130669000	-0.886736000
S	1.983428000	-2.347607000	-1.612829000
O	0.979636000	-1.487409000	-2.377680000
O	1.338466000	-3.014212000	-0.464930000
O	2.790755000	-3.166083000	-2.519106000
C	4.438541000	-1.170910000	-1.254103000
C	5.344098000	-0.261516000	-0.717189000
C	3.577414000	0.743144000	0.556610000
C	0.848719000	-1.282460000	1.986311000
C	-0.303220000	-2.043572000	2.208667000
H	-1.138401000	-1.973336000	1.521483000
C	-0.365332000	-2.925203000	3.285703000
H	-1.263655000	-3.516270000	3.441917000
C	0.725197000	-3.063529000	4.141893000
H	0.679170000	-3.759385000	4.975792000
C	1.883910000	-2.320747000	3.915229000
H	2.745390000	-2.438577000	4.567623000
C	1.948438000	-1.435270000	2.842291000
C	0.665082000	1.547269000	1.351963000
C	0.495416000	1.591868000	2.745498000
H	0.519528000	0.666003000	3.308427000
C	0.309303000	2.786928000	3.436730000
H	0.186534000	2.765236000	4.516197000
C	0.299388000	3.993880000	2.748680000
H	0.179309000	4.936025000	3.276223000
C	0.448760000	3.983796000	1.366172000
H	0.469237000	4.916932000	0.812950000
C	0.607440000	2.783927000	0.669863000
C	4.927599000	0.712979000	0.193964000
C	5.898687000	1.721413000	0.749623000

H	6.093440000	2.519683000	0.021681000
H	5.513473000	2.194235000	1.658880000
H	6.862410000	1.258738000	0.989996000
H	6.389923000	-0.308569000	-1.013859000
H	3.244005000	1.503790000	1.258193000
H	4.751845000	-1.927624000	-1.966537000
C	-1.857366000	-1.467419000	-2.896265000
H	-2.816902000	-1.793592000	-2.509651000
H	-1.169521000	-2.243238000	-3.220135000
C	-1.579434000	-0.149906000	-3.146168000
H	-0.676716000	0.132868000	-3.681740000
H	-2.307887000	0.637201000	-2.970515000
H	2.861880000	-0.875222000	2.662618000
S	0.674124000	2.957339000	-1.121525000
O	1.383471000	1.809889000	-1.670752000
O	1.298539000	4.313240000	-1.389244000
O	-0.762517000	3.123374000	-1.592524000
Li	-0.303174000	4.826337000	-2.235028000
C	-2.119238000	0.306259000	-0.114670000
H	-1.998799000	1.345077000	-0.440233000
H	-1.903699000	0.273279000	0.959573000
C	-3.580117000	-0.111892000	-0.323270000
H	-3.803608000	-0.242993000	-1.389358000
C	-4.509908000	0.999379000	0.219431000
H	-4.171786000	1.934331000	-0.246983000
H	-4.327485000	1.116935000	1.295736000
C	-3.877774000	-1.453412000	0.325578000
C	-6.013345000	0.848701000	-0.036584000
H	-6.409000000	-0.009346000	0.515808000
H	-6.184307000	0.632822000	-1.100630000
C	-6.784199000	2.106911000	0.359747000
H	-7.858601000	1.987922000	0.179690000
H	-6.649710000	2.335951000	1.424140000
H	-6.445069000	2.980315000	-0.211505000
F	-5.095413000	-1.929923000	-0.014352000
F	-3.843774000	-1.379062000	1.674796000
F	-2.991773000	-2.411325000	-0.029372000

70

A2,E21(Coor5)

Pd	-0.508497000	-1.324380000	-1.004045000
P	0.736473000	-0.072156000	0.589118000
C	2.367814000	0.514254000	-0.009622000
C	3.133604000	-0.264098000	-0.896742000
S	2.566498000	-1.855709000	-1.518990000
O	1.253682000	-1.507787000	-2.223204000

O	2.323657000	-2.684039000	-0.324147000
O	3.573999000	-2.310992000	-2.478451000
C	4.389092000	0.177488000	-1.296523000
C	4.897478000	1.384722000	-0.820548000
C	2.895482000	1.721130000	0.454636000
C	1.160220000	-1.027723000	2.099040000
C	0.425995000	-2.177754000	2.406588000
H	-0.366751000	-2.506564000	1.743556000
C	0.726042000	-2.916277000	3.550037000
H	0.153018000	-3.811832000	3.774781000
C	1.766384000	-2.519488000	4.387384000
H	2.005944000	-3.102355000	5.273155000
C	2.510155000	-1.380080000	4.079098000
H	3.332747000	-1.074254000	4.720510000
C	2.210987000	-0.637061000	2.940436000
C	-0.116847000	1.439567000	1.267807000
C	-0.513229000	1.399692000	2.615185000
H	-0.237096000	0.543695000	3.220424000
C	-1.251698000	2.423771000	3.204199000
H	-1.533478000	2.344028000	4.250543000
C	-1.631000000	3.530111000	2.453353000
H	-2.217360000	4.329937000	2.896761000
C	-1.260782000	3.597785000	1.114194000
H	-1.579006000	4.433507000	0.499936000
C	-0.514856000	2.574894000	0.526027000
C	4.160517000	2.175158000	0.060407000
C	4.683598000	3.492891000	0.567060000
H	4.079487000	4.323714000	0.181854000
H	4.648651000	3.546860000	1.661876000
H	5.720033000	3.657473000	0.256306000
H	5.882680000	1.714610000	-1.143190000
H	2.309682000	2.338301000	1.131045000
H	4.955361000	-0.441035000	-1.985620000
C	-1.054645000	-3.269593000	-2.072853000
H	-1.723975000	-3.847722000	-1.446730000
H	-0.042463000	-3.643435000	-2.196214000
C	-1.509349000	-2.223888000	-2.832221000
H	-0.869155000	-1.766264000	-3.582375000
H	-2.559609000	-1.952792000	-2.832048000
H	2.807507000	0.237696000	2.698327000
S	-0.090341000	2.829328000	-1.202631000
O	0.060805000	1.511511000	-1.812795000
O	1.136083000	3.718647000	-1.241343000
O	-1.190544000	3.696667000	-1.782862000
Li	0.130292000	4.988153000	-2.193360000

C	-2.279910000	-0.968185000	0.031534000
H	-2.003833000	-0.578386000	1.017254000
C	-3.066284000	0.101919000	-0.733796000
H	-2.350010000	0.831952000	-1.127725000
C	-4.127044000	0.856280000	0.084122000
H	-3.653670000	1.282840000	0.980630000
H	-4.894192000	0.160207000	0.443293000
C	-4.789689000	1.976855000	-0.719491000
H	-5.263869000	1.548634000	-1.614338000
H	-4.016113000	2.665616000	-1.085772000
C	-5.832121000	2.750307000	0.085407000
H	-6.297141000	3.541331000	-0.514654000
H	-6.631734000	2.088428000	0.440308000
H	-5.381303000	3.222597000	0.967438000
H	-3.543954000	-0.345842000	-1.613461000
C	-3.095629000	-2.199646000	0.330749000
F	-4.050530000	-1.962542000	1.266557000
F	-3.748612000	-2.690756000	-0.749256000
F	-2.352322000	-3.221114000	0.828153000

70

A2,E12(Coor6)

Pd	-1.099610000	0.415835000	0.129039000
P	1.241278000	-0.421128000	0.425653000
C	2.096739000	-0.820867000	-1.149749000
C	1.361290000	-1.345814000	-2.224119000
S	-0.406783000	-1.680008000	-2.109550000
O	-1.032665000	-0.299335000	-1.865803000
O	-0.564801000	-2.540289000	-0.923131000
O	-0.815216000	-2.207989000	-3.411205000
C	2.003087000	-1.651448000	-3.421344000
C	3.371161000	-1.441747000	-3.559869000
C	3.470666000	-0.615015000	-1.310395000
C	1.357488000	-1.998163000	1.366285000
C	0.237443000	-2.434498000	2.082453000
H	-0.685266000	-1.864179000	2.037936000
C	0.280464000	-3.626904000	2.802360000
H	-0.601028000	-3.959957000	3.343729000
C	1.439029000	-4.400792000	2.804701000
H	1.469487000	-5.335386000	3.359145000
C	2.553254000	-3.985118000	2.075099000
H	3.452325000	-4.595976000	2.055341000
C	2.512550000	-2.792953000	1.356568000
C	2.421037000	0.693932000	1.353948000
C	2.954998000	0.223187000	2.567905000
H	2.703631000	-0.777479000	2.899145000

C	3.809453000	0.988329000	3.356600000
H	4.194894000	0.571934000	4.283451000
C	4.177701000	2.266622000	2.950971000
H	4.860904000	2.866403000	3.545634000
C	3.650421000	2.772353000	1.769913000
H	3.928352000	3.767179000	1.436472000
C	2.771772000	2.013798000	0.992205000
C	4.128020000	-0.922285000	-2.505744000
H	3.856738000	-1.682600000	-4.503345000
H	4.048579000	-0.192497000	-0.491700000
H	1.407426000	-2.051021000	-4.235961000
C	-3.031432000	0.932100000	-0.406844000
C	-1.219277000	2.085232000	1.527041000
C	-1.234531000	0.909274000	2.257319000
H	3.378726000	-2.486526000	0.776056000
S	2.089047000	2.887024000	-0.421707000
O	1.289051000	4.041270000	0.162898000
O	1.315594000	1.941515000	-1.215874000
O	3.245774000	3.565948000	-1.124900000
Li	2.399992000	5.220016000	-0.794239000
C	5.612848000	-0.712787000	-2.647721000
H	5.857883000	-0.246399000	-3.608559000
H	6.006146000	-0.071364000	-1.852647000
H	6.154216000	-1.666985000	-2.603830000
H	-0.327185000	0.551401000	2.736267000
H	-2.158877000	0.472581000	2.621049000
H	-0.304695000	2.652406000	1.376116000
H	-2.138000000	2.623486000	1.314741000
C	-4.182355000	0.756693000	0.574317000
H	-3.928066000	1.189620000	1.552019000
C	-5.459844000	1.503149000	0.111734000
H	-6.229341000	1.389478000	0.886407000
H	-5.203402000	2.571700000	0.089773000
C	-6.045877000	1.108929000	-1.246284000
H	-5.276842000	1.197980000	-2.023594000
H	-6.345652000	0.055437000	-1.231522000
C	-7.247539000	1.976615000	-1.615702000
H	-6.970179000	3.036525000	-1.678552000
H	-7.663744000	1.684011000	-2.586105000
H	-8.047725000	1.887919000	-0.870139000
C	-4.462171000	-0.709689000	0.869155000
H	-3.145398000	0.301089000	-1.290118000
H	-2.959730000	1.978249000	-0.726264000
F	-3.436184000	-1.293818000	1.535215000
F	-5.551590000	-0.854727000	1.657753000

F	-4.673088000	-1.439517000	-0.238453000

70

A2,E21(Coor6)

Pd	1.362502000	-0.509689000	-0.026471000
P	-0.793330000	0.211148000	0.579266000
C	-1.478087000	1.550357000	-0.468695000
C	-0.631564000	2.543216000	-0.986179000
S	1.139846000	2.553669000	-0.651471000
O	1.621182000	1.247734000	-1.276214000
O	1.239232000	2.504077000	0.821284000
O	1.687217000	3.722433000	-1.340570000
C	-1.162809000	3.567005000	-1.764384000
C	-2.527482000	3.612467000	-2.032331000
C	-2.846615000	1.609537000	-0.751615000
C	-0.858354000	0.895508000	2.280503000
C	0.188029000	0.619355000	3.167096000
H	1.034968000	0.029358000	2.828876000
C	0.169142000	1.137374000	4.459869000
H	0.992861000	0.925415000	5.136261000
C	-0.888431000	1.944804000	4.874673000
H	-0.897301000	2.356684000	5.880583000
C	-1.924643000	2.241840000	3.989725000
H	-2.740543000	2.889214000	4.300700000
C	-1.909421000	1.723794000	2.696894000
C	-2.102447000	-1.118881000	0.583564000
C	-2.828121000	-1.323313000	1.768133000
H	-2.614072000	-0.703620000	2.631080000
C	-3.825279000	-2.290629000	1.874180000
H	-4.359554000	-2.405362000	2.813427000
C	-4.140649000	-3.087492000	0.780627000
H	-4.931909000	-3.829538000	0.840741000
C	-3.426934000	-2.924151000	-0.401679000
H	-3.669722000	-3.521186000	-1.274474000
C	-2.409452000	-1.973530000	-0.498974000
C	-3.391200000	2.633765000	-1.532882000
C	-4.863151000	2.661470000	-1.851240000
H	-5.057072000	2.235987000	-2.844566000
H	-5.444149000	2.080250000	-1.127649000
H	-5.252645000	3.685438000	-1.854731000
H	-2.926700000	4.421066000	-2.641242000
H	-3.510036000	0.838721000	-0.366794000
H	-0.484508000	4.321500000	-2.150469000
C	3.543401000	-1.047507000	-0.469112000
H	3.836977000	-1.651849000	0.381398000
C	2.730163000	-1.548463000	-1.451630000

H	2.584176000	-0.998885000	-2.377503000
H	2.348453000	-2.563473000	-1.406566000
H	-2.708276000	1.978122000	2.005589000
S	-1.511699000	-1.981703000	-2.058933000
O	-0.941743000	-0.657307000	-2.264703000
O	-2.496910000	-2.454203000	-3.110074000
O	-0.504977000	-3.118427000	-1.970440000
Li	-1.378613000	-3.940780000	-3.413854000
C	0.989525000	-2.240403000	1.061021000
H	0.472390000	-2.878758000	0.334577000
H	0.273255000	-2.000442000	1.855933000
C	2.170015000	-2.973312000	1.686714000
H	2.846479000	-3.359921000	0.912712000
H	2.762840000	-2.293645000	2.314983000
C	1.702054000	-4.155788000	2.544465000
H	1.125561000	-4.871386000	1.945849000
H	2.553754000	-4.690436000	2.982394000
H	1.059285000	-3.817103000	3.365890000
C	4.441935000	0.148413000	-0.650354000
F	5.723184000	-0.291890000	-0.534961000
F	4.265238000	1.071773000	0.304108000
F	4.330032000	0.729689000	-1.844137000

70

A2,E12(TS3)

Pd	-0.865615000	0.752048000	0.064661000
P	1.133296000	-0.335166000	0.506839000
C	1.796260000	-1.324768000	-0.892627000
C	0.922764000	-2.010766000	-1.752644000
S	-0.865099000	-2.018200000	-1.513137000
O	-1.251393000	-0.541067000	-1.623651000
O	-1.058525000	-2.527557000	-0.145168000
O	-1.432196000	-2.788387000	-2.622419000
C	1.436677000	-2.752286000	-2.812207000
C	2.810188000	-2.823970000	-3.023562000
C	3.173205000	-1.408978000	-1.122216000
C	1.014036000	-1.542498000	1.883241000
C	-0.049061000	-1.438762000	2.786786000
H	-0.807300000	-0.675179000	2.641600000
C	-0.169502000	-2.347037000	3.836530000
H	-1.006404000	-2.264148000	4.524859000
C	0.762626000	-3.371981000	3.986552000
H	0.661322000	-4.086173000	4.799834000
C	1.813416000	-3.493784000	3.077358000
H	2.530794000	-4.304493000	3.176348000
C	1.937892000	-2.586333000	2.028400000

C	2.557597000	0.764727000	1.006800000
C	3.224226000	0.480092000	2.209116000
H	2.899888000	-0.367153000	2.802404000
C	4.298601000	1.241714000	2.664619000
H	4.782030000	0.975735000	3.600847000
C	4.755272000	2.318909000	1.915272000
H	5.607693000	2.906304000	2.245105000
C	4.105392000	2.639894000	0.728169000
H	4.457519000	3.463264000	0.115690000
C	3.010773000	1.893846000	0.286794000
C	3.701487000	-2.158634000	-2.178443000
H	3.193519000	-3.403601000	-3.860880000
H	3.859388000	-0.868639000	-0.474707000
H	0.737146000	-3.264125000	-3.465571000
C	-2.879592000	1.644967000	-0.532172000
C	-1.648659000	2.780466000	0.814259000
C	-0.600532000	2.161827000	1.555799000
H	2.746153000	-2.699885000	1.311043000
S	2.215863000	2.521968000	-1.202960000
O	1.239967000	3.595527000	-0.741766000
O	1.625663000	1.402419000	-1.921966000
O	3.280117000	3.303473000	-1.949745000
Li	2.232271000	4.848903000	-1.722536000
C	5.190447000	-2.256105000	-2.384657000
H	5.442364000	-2.303949000	-3.449499000
H	5.713554000	-1.396713000	-1.952490000
H	5.597762000	-3.160759000	-1.913651000
H	0.398722000	2.574819000	1.446258000
H	-0.833407000	1.749350000	2.535589000
H	-1.347681000	3.530527000	0.089265000
H	-2.591076000	2.969956000	1.315521000
C	-4.178773000	1.397623000	0.228589000
H	-4.448160000	2.298739000	0.797631000
C	-5.346664000	1.139731000	-0.756704000
H	-6.288073000	1.228013000	-0.199199000
H	-5.339340000	1.970513000	-1.476084000
C	-5.341648000	-0.188640000	-1.520634000
H	-4.372941000	-0.353975000	-2.006213000
H	-5.471256000	-1.018354000	-0.817010000
C	-6.452164000	-0.236649000	-2.568415000
H	-6.323541000	0.548956000	-3.323757000
H	-6.456735000	-1.199963000	-3.089635000
H	-7.440844000	-0.099200000	-2.111932000
C	-4.082847000	0.308744000	1.285163000
H	-2.713948000	0.860570000	-1.276497000

H	-2.946782000	2.589209000	-1.073167000
F	-3.293169000	0.696968000	2.322883000
F	-5.295275000	0.042755000	1.818006000
F	-3.581350000	-0.841580000	0.823465000

70

A2,E21(TS3)

Pd	0.939804000	0.155418000	-0.543202000
P	-1.312850000	-0.361851000	-0.404693000
C	-1.892614000	-0.789674000	1.284020000
C	-1.058209000	-1.499913000	2.162517000
S	0.601308000	-2.035553000	1.704101000
O	1.320402000	-0.718003000	1.391056000
O	0.428137000	-2.850792000	0.489004000
O	1.175913000	-2.676061000	2.888066000
C	-1.517730000	-1.835298000	3.432757000
C	-2.797943000	-1.471349000	3.838579000
C	-3.173902000	-0.428539000	1.712296000
C	-1.793330000	-1.820249000	-1.409860000
C	-0.956699000	-2.234913000	-2.451467000
H	-0.022825000	-1.710834000	-2.629516000
C	-1.291045000	-3.345726000	-3.222969000
H	-0.626603000	-3.665387000	-4.021296000
C	-2.457375000	-4.059129000	-2.953433000
H	-2.711930000	-4.932413000	-3.548601000
C	-3.286462000	-3.663909000	-1.903746000
H	-4.186361000	-4.229249000	-1.675378000
C	-2.955774000	-2.552572000	-1.132304000
C	-2.477370000	0.977269000	-0.979560000
C	-3.427859000	0.652681000	-1.960106000
H	-3.467409000	-0.361783000	-2.340008000
C	-4.333331000	1.587918000	-2.457799000
H	-5.052384000	1.284422000	-3.213948000
C	-4.323968000	2.890702000	-1.975205000
H	-5.039337000	3.624171000	-2.336676000
C	-3.382991000	3.250131000	-1.015363000
H	-3.369461000	4.255379000	-0.607648000
C	-2.458700000	2.319837000	-0.537150000
C	-3.645663000	-0.758710000	2.986784000
C	-5.016396000	-0.326025000	3.436757000
H	-4.961657000	0.605065000	4.015912000
H	-5.681627000	-0.143167000	2.586392000
H	-5.484472000	-1.080928000	4.077881000
H	-3.140738000	-1.742015000	4.835125000
H	-3.822507000	0.135758000	1.046630000
H	-0.850734000	-2.382288000	4.091573000

C	3.221303000	0.418160000	-0.532435000
C	0.760597000	1.150267000	-2.353519000
C	2.052181000	1.594961000	-1.951159000
H	-3.595067000	-2.264353000	-0.302389000
S	-1.241350000	2.973307000	0.618551000
O	-0.880474000	1.916795000	1.552713000
O	-1.863333000	4.215408000	1.226944000
O	-0.100350000	3.519996000	-0.227424000
Li	-0.469558000	5.225781000	0.463204000
C	4.019178000	1.611001000	-0.021477000
H	4.324434000	2.253250000	-0.857295000
H	3.344090000	2.203491000	0.609687000
C	5.270983000	1.253353000	0.802275000
H	5.971648000	0.678878000	0.183455000
H	5.781452000	2.198973000	1.037663000
C	4.997259000	0.500158000	2.105075000
H	4.538596000	-0.471873000	1.890254000
H	4.258762000	1.055753000	2.699598000
C	6.264874000	0.284732000	2.929575000
H	6.730685000	1.239676000	3.205364000
H	6.047054000	-0.260277000	3.854653000
H	7.008073000	-0.295954000	2.369119000
H	2.973502000	-0.227394000	0.317632000
C	3.941179000	-0.474041000	-1.517106000
F	4.800481000	-1.302076000	-0.886294000
F	3.096690000	-1.254530000	-2.222806000
F	4.680565000	0.215926000	-2.424572000
H	0.688900000	0.467218000	-3.198763000
H	-0.064260000	1.850453000	-2.248399000
H	2.902898000	1.406400000	-2.594975000
H	2.095214000	2.526768000	-1.396097000

70

A2,E12(p3)

Pd	-0.360543000	-0.396664000	-0.999215000
P	1.282511000	-0.201234000	0.532090000
C	2.909691000	-0.701206000	-0.152143000
C	3.008486000	-1.761315000	-1.069545000
S	1.577756000	-2.711936000	-1.635734000
O	0.717215000	-1.671497000	-2.346545000
O	0.930708000	-3.193605000	-0.399308000
O	2.093554000	-3.713617000	-2.570673000
C	4.258570000	-2.132191000	-1.556960000
C	5.406784000	-1.461265000	-1.147328000
C	4.074591000	-0.036149000	0.245098000
C	1.065864000	-1.260784000	2.013857000

C	-0.226246000	-1.559584000	2.459032000
H	-1.082000000	-1.190254000	1.903214000
C	-0.417740000	-2.353530000	3.587007000
H	-1.426922000	-2.584244000	3.917958000
C	0.680722000	-2.866806000	4.274714000
H	0.531581000	-3.496128000	5.148375000
C	1.971318000	-2.584898000	3.828893000
H	2.831487000	-2.996781000	4.350349000
C	2.166051000	-1.786760000	2.703713000
C	1.551522000	1.498856000	1.246354000
C	1.635046000	1.615223000	2.642534000
H	1.556354000	0.721268000	3.250594000
C	1.825551000	2.841690000	3.275891000
H	1.886547000	2.879054000	4.360202000
C	1.950827000	4.001160000	2.521059000
H	2.118858000	4.961889000	2.999810000
C	1.865094000	3.920524000	1.134455000
H	1.989793000	4.808425000	0.523874000
C	1.651261000	2.695587000	0.501053000
C	5.334010000	-0.400096000	-0.241602000
H	6.374065000	-1.766632000	-1.541233000
H	4.006302000	0.794747000	0.942981000
H	4.307247000	-2.955693000	-2.262419000
C	-3.911961000	-0.380602000	-1.118354000
C	-2.639113000	0.451840000	-1.346363000
C	-1.783996000	0.756537000	-0.143354000
H	3.174573000	-1.587492000	2.353504000
S	1.496540000	2.776671000	-1.292574000
O	0.030799000	3.044682000	-1.594656000
O	2.042298000	1.547889000	-1.852049000
O	2.189523000	4.059492000	-1.711622000
Li	0.537009000	4.672388000	-2.375583000
C	6.569413000	0.353706000	0.175677000
H	6.838553000	1.109394000	-0.573840000
H	6.421974000	0.875195000	1.127181000
H	7.429373000	-0.316086000	0.285522000
H	-1.453864000	1.790488000	-0.058641000
H	-2.168195000	0.367821000	0.801184000
H	-2.022903000	-0.167724000	-2.093007000
H	-2.832811000	1.354255000	-1.938376000
C	-5.204388000	0.412295000	-0.851777000
H	-5.309735000	1.166927000	-1.643994000
C	-6.456903000	-0.486292000	-0.920958000
H	-7.346549000	0.151200000	-0.851438000
H	-6.476603000	-0.926349000	-1.927473000

C	-6.565688000	-1.609582000	0.114209000
H	-5.683980000	-2.260798000	0.058952000
H	-6.572956000	-1.185753000	1.124954000
C	-7.824154000	-2.450522000	-0.093734000
H	-7.830512000	-2.919897000	-1.085194000
H	-7.894361000	-3.249749000	0.652269000
H	-8.730096000	-1.837422000	-0.011294000
C	-5.153786000	1.223049000	0.433514000
H	-3.729852000	-1.091618000	-0.305049000
H	-4.108172000	-0.984925000	-2.012207000
F	-4.305126000	2.269308000	0.329700000
F	-6.359558000	1.740032000	0.748018000
F	-4.749472000	0.493879000	1.497241000

70

A2,E21(p3)

Pd	-0.363520000	-1.377410000	-0.037723000
P	1.382360000	-0.114878000	0.526839000
C	2.335496000	0.459675000	-0.926659000
C	2.491993000	-0.358889000	-2.061771000
S	1.793912000	-2.023915000	-2.184074000
O	0.294141000	-1.793673000	-2.029189000
O	2.354112000	-2.747848000	-1.028096000
O	2.128684000	-2.508305000	-3.524080000
C	3.258470000	0.093445000	-3.128859000
C	3.871772000	1.344589000	-3.083043000
C	2.959145000	1.709640000	-0.898051000
C	2.610295000	-1.005508000	1.555636000
C	2.219133000	-2.156017000	2.248558000
H	1.202642000	-2.523797000	2.143039000
C	3.137345000	-2.846291000	3.036702000
H	2.826187000	-3.745172000	3.562119000
C	4.453890000	-2.399035000	3.130654000
H	5.172427000	-2.944613000	3.736970000
C	4.853555000	-1.260703000	2.430511000
H	5.883878000	-0.918931000	2.485732000
C	3.938062000	-0.566150000	1.644158000
C	0.985290000	1.404324000	1.530247000
C	1.449246000	1.419949000	2.856434000
H	2.080303000	0.609658000	3.202742000
C	1.123746000	2.438331000	3.749152000
H	1.510536000	2.401759000	4.763924000
C	0.301711000	3.482196000	3.343372000
H	0.026337000	4.275667000	4.032428000
C	-0.177920000	3.495742000	2.038024000
H	-0.848777000	4.282548000	1.710084000

C	0.156862000	2.481422000	1.138206000
C	3.733948000	2.171814000	-1.969448000
H	4.465576000	1.679786000	-3.930884000
H	2.834023000	2.354210000	-0.031551000
H	3.362642000	-0.554526000	-3.993396000
C	-3.395922000	-0.557775000	0.287711000
C	-2.570221000	-0.332190000	1.575159000
C	-1.282064000	-1.131664000	1.760488000
C	-4.746591000	0.182713000	0.387785000
H	-5.453125000	-0.437136000	0.955258000
H	-4.574234000	1.079463000	0.998007000
C	-5.353736000	0.622691000	-0.947662000
H	-5.595439000	-0.253564000	-1.559975000
H	-4.592318000	1.188471000	-1.502232000
C	-6.604160000	1.496368000	-0.795963000
H	-6.354964000	2.385096000	-0.197280000
H	-6.886976000	1.870512000	-1.789207000
H	4.262211000	0.306962000	1.084806000
S	-0.510381000	2.697163000	-0.521185000
O	-1.769609000	3.528208000	-0.356741000
O	-0.707178000	1.376216000	-1.108151000
O	0.440383000	3.614382000	-1.263303000
Li	-0.965341000	4.844687000	-1.451867000
C	4.372225000	3.534110000	-1.911217000
H	5.006300000	3.715105000	-2.784735000
H	3.607539000	4.320069000	-1.879962000
H	4.994298000	3.650563000	-1.015379000
H	-0.685902000	-0.686473000	2.562090000
H	-1.481661000	-2.178682000	2.033520000
H	-2.336331000	0.738266000	1.592563000
H	-3.229248000	-0.516972000	2.438871000
H	-2.830111000	-0.155422000	-0.563232000
C	-3.591647000	-2.030566000	-0.003433000
F	-4.427433000	-2.270310000	-1.023666000
F	-2.402227000	-2.633992000	-0.361251000
F	-4.050680000	-2.714239000	1.059930000
C	-7.805685000	0.783660000	-0.175433000
H	-7.608424000	0.466405000	0.854996000
H	-8.683835000	1.439299000	-0.151901000
H	-8.074150000	-0.110805000	-0.751227000

88

A3,E12(Coor5)

Pd	1.631644000	-1.431071000	0.311852000
P	-0.274199000	-0.005939000	0.132147000
C	-1.753771000	-0.836948000	0.842424000

C	-1.982102000	-2.208554000	0.640778000
S	-0.878586000	-3.219124000	-0.356697000
O	0.402274000	-3.213379000	0.478807000
O	-0.691764000	-2.489943000	-1.623900000
O	-1.471946000	-4.557688000	-0.418862000
C	-3.056846000	-2.836648000	1.263190000
C	-3.906057000	-2.113863000	2.096984000
C	-2.618809000	-0.132519000	1.684770000
C	-0.584124000	0.648682000	-1.560042000
C	0.542290000	0.589064000	-2.393300000
H	1.426557000	0.069362000	-2.044759000
C	0.545929000	1.154812000	-3.663361000
H	1.440024000	1.089894000	-4.276782000
C	-0.600880000	1.787066000	-4.130486000
H	-0.621762000	2.236582000	-5.120142000
C	-1.738712000	1.816884000	-3.330866000
H	-2.650569000	2.274021000	-3.705503000
C	-1.766269000	1.251047000	-2.048359000
C	-0.038711000	1.488561000	1.169652000
C	0.001375000	2.774056000	0.625219000
H	-0.218948000	2.909870000	-0.427291000
C	0.304347000	3.885352000	1.413905000
H	0.331873000	4.874719000	0.966452000
C	0.571265000	3.714663000	2.767184000
H	0.805715000	4.572037000	3.393278000
C	0.551388000	2.441421000	3.337783000
H	0.770705000	2.321916000	4.393342000
C	0.261270000	1.330028000	2.543143000
O	0.249317000	0.056316000	3.003213000
C	0.497911000	-0.183850000	4.373993000
H	1.505775000	0.141575000	4.662885000
H	0.412545000	-1.263306000	4.504542000
H	-0.243162000	0.319472000	5.007998000
C	-3.700076000	-0.750631000	2.320898000
C	2.850038000	0.232764000	0.388224000
H	2.857006000	0.442999000	1.464864000
C	-4.625316000	0.044659000	3.203743000
H	-4.076095000	0.777269000	3.805754000
H	-5.353546000	0.601166000	2.601001000
H	-5.183457000	-0.604957000	3.885951000
H	-4.736044000	-2.620153000	2.585822000
H	-2.449562000	0.927525000	1.852042000
H	-3.199550000	-3.899483000	1.093567000
C	3.250322000	-3.037542000	0.134355000
H	4.031045000	-2.654186000	-0.513554000

H	2.632246000	-3.839011000	-0.259563000
C	3.148804000	-2.671447000	1.449323000
H	2.469064000	-3.197221000	2.114954000
H	3.858203000	-1.987956000	1.909395000
C	-3.093584000	1.268794000	-1.364269000
C	-3.938125000	0.148702000	-1.497835000
C	-3.604725000	2.427287000	-0.759064000
C	-5.241837000	0.167360000	-0.987028000
C	-4.904024000	2.453073000	-0.236522000
C	-5.703256000	1.319469000	-0.359790000
H	-5.888040000	-0.697102000	-1.084492000
H	-5.298307000	3.342228000	0.241558000
H	-6.717491000	1.341530000	0.031995000
O	-2.764718000	3.499793000	-0.734197000
O	-3.396859000	-0.893131000	-2.159035000
C	-3.235306000	4.701038000	-0.162173000
H	-3.471787000	4.578097000	0.903021000
H	-2.422243000	5.421913000	-0.266617000
H	-4.121828000	5.079151000	-0.688580000
C	-4.152664000	-2.076854000	-2.325315000
H	-3.483080000	-2.779670000	-2.821082000
H	-4.458224000	-2.494510000	-1.358512000
H	-5.040335000	-1.898362000	-2.947482000
C	4.302565000	0.165868000	-0.103057000
H	4.793454000	-0.753403000	0.240335000
C	5.095895000	1.364872000	0.470182000
H	4.885125000	1.384785000	1.547820000
H	4.674185000	2.294242000	0.065761000
C	6.616885000	1.358112000	0.281430000
H	7.020550000	0.386631000	0.600491000
H	6.871328000	1.460988000	-0.777951000
C	7.290658000	2.475877000	1.075865000
H	7.098644000	2.374795000	2.151373000
H	8.376597000	2.467476000	0.929295000
H	6.921614000	3.461240000	0.765588000
H	2.359483000	1.083618000	-0.094572000
C	4.376710000	0.126367000	-1.620241000
F	5.631550000	-0.095307000	-2.067786000
F	3.954727000	1.281524000	-2.177068000
F	3.616150000	-0.859477000	-2.151667000

88

A3,E21(Coor5)

Pd	1.813923000	-1.406592000	-0.237649000
P	-0.174457000	-0.105121000	-0.077622000
C	-1.335671000	-0.867394000	1.135274000

C	-1.499039000	-2.261704000	1.222478000
S	-0.700214000	-3.408229000	0.089073000
O	0.787599000	-3.184587000	0.379184000
O	-1.056336000	-2.969463000	-1.268511000
O	-1.105975000	-4.753966000	0.502668000
C	-2.297048000	-2.809168000	2.222266000
C	-2.939727000	-1.984186000	3.142738000
C	-2.001739000	-0.060312000	2.060804000
C	-1.003192000	0.137089000	-1.707199000
C	-0.256198000	-0.329125000	-2.798950000
H	0.677385000	-0.847942000	-2.611308000
C	-0.681052000	-0.152363000	-4.109791000
H	-0.074701000	-0.529258000	-4.928597000
C	-1.880923000	0.505299000	-4.354469000
H	-2.228972000	0.663130000	-5.372236000
C	-2.654503000	0.935546000	-3.282329000
H	-3.615110000	1.409332000	-3.464795000
C	-2.256985000	0.748429000	-1.950775000
C	0.171560000	1.557218000	0.604530000
C	0.008725000	2.722836000	-0.145136000
H	-0.434563000	2.660841000	-1.131669000
C	0.399866000	3.963922000	0.359742000
H	0.267045000	4.857997000	-0.242039000
C	0.964109000	4.044966000	1.627585000
H	1.268010000	5.006978000	2.032548000
C	1.164774000	2.893281000	2.389649000
H	1.622592000	2.968792000	3.370091000
C	0.791604000	1.650433000	1.873648000
O	1.005599000	0.472818000	2.507205000
C	1.584198000	0.478358000	3.798226000
H	2.595713000	0.903490000	3.782028000
H	1.633134000	-0.567477000	4.103575000
H	0.963199000	1.037472000	4.509413000
C	-2.807979000	-0.596699000	3.071449000
C	3.016796000	0.284681000	-0.490889000
H	2.476617000	1.082107000	0.025430000
C	-3.519246000	0.312017000	4.038813000
H	-2.835228000	1.049171000	4.475718000
H	-4.315225000	0.871338000	3.532249000
H	-3.973537000	-0.253741000	4.858490000
H	-3.550592000	-2.428193000	3.926224000
H	-1.896127000	1.019073000	1.996993000
H	-2.392607000	-3.889453000	2.268041000
C	3.120314000	-2.906124000	-1.418167000
H	3.691560000	-2.341860000	-2.145764000

H	2.281715000	-3.494057000	-1.783771000
C	3.533643000	-3.019860000	-0.123091000
H	3.026862000	-3.693497000	0.561643000
H	4.459004000	-2.564073000	0.211129000
C	-3.279235000	1.122572000	-0.926739000
C	-4.140970000	0.116657000	-0.443520000
C	-3.573775000	2.459725000	-0.611283000
C	-5.213795000	0.430382000	0.400264000
C	-4.631534000	2.782195000	0.248629000
C	-5.436321000	1.759851000	0.742683000
H	-5.870386000	-0.346448000	0.774592000
H	-4.844386000	3.811497000	0.512477000
H	-6.268628000	2.009378000	1.396551000
O	-2.791216000	3.396525000	-1.214581000
O	-3.861450000	-1.127512000	-0.880633000
C	-3.078840000	4.758840000	-0.980586000
H	-2.938187000	5.027884000	0.074550000
H	-2.372001000	5.322870000	-1.592393000
H	-4.101958000	5.013215000	-1.287186000
C	-4.704184000	-2.199444000	-0.506352000
H	-4.268387000	-3.083891000	-0.971999000
H	-4.720418000	-2.335398000	0.581494000
H	-5.727527000	-2.048229000	-0.875728000
C	4.360232000	0.074929000	0.221458000
H	4.189460000	-0.577650000	1.087275000
H	5.064431000	-0.456455000	-0.433649000
C	5.022154000	1.356485000	0.754923000
H	4.302886000	1.887365000	1.396212000
H	5.254391000	2.034484000	-0.072045000
C	3.157472000	0.767516000	-1.913364000
F	4.126795000	1.709881000	-2.054909000
F	3.479194000	-0.205129000	-2.803843000
F	2.021973000	1.343625000	-2.363456000
C	6.295637000	1.066915000	1.551632000
H	6.062312000	0.380545000	2.379094000
H	7.011826000	0.534243000	0.909721000
C	6.953274000	2.328772000	2.106716000
H	6.274163000	2.867079000	2.779639000
H	7.862630000	2.092256000	2.671101000
H	7.231567000	3.017657000	1.299922000

88

A3,E12(Coor6)

Pd	-1.889321000	0.151347000	-0.568169000
P	0.630511000	-0.168750000	-0.294453000
C	1.261429000	0.808963000	1.135576000

C	0.697209000	2.061564000	1.434705000
S	-0.548179000	2.814543000	0.382080000
O	-1.769775000	1.899273000	0.591213000
O	-0.042456000	2.704474000	-0.997326000
O	-0.811367000	4.149105000	0.920235000
C	1.072097000	2.747275000	2.585493000
C	2.006140000	2.191343000	3.457151000
C	2.201069000	0.276851000	2.021008000
C	1.610178000	0.147075000	-1.829293000
C	0.802292000	0.375981000	-2.954743000
H	-0.266565000	0.495938000	-2.811217000
C	1.335058000	0.506638000	-4.232798000
H	0.677063000	0.691782000	-5.077617000
C	2.711338000	0.414562000	-4.408741000
H	3.150909000	0.506614000	-5.398737000
C	3.530812000	0.242202000	-3.298452000
H	4.609992000	0.220265000	-3.422784000
C	3.014699000	0.125477000	-1.999586000
C	0.997011000	-1.913269000	0.153144000
C	1.671856000	-2.788117000	-0.701527000
H	2.116944000	-2.402620000	-1.611355000
C	1.799395000	-4.145566000	-0.399394000
H	2.329052000	-4.803207000	-1.082539000
C	1.246975000	-4.644538000	0.774245000
H	1.344294000	-5.698110000	1.023959000
C	0.548123000	-3.801425000	1.639457000
H	0.108455000	-4.205017000	2.545164000
C	0.405514000	-2.448308000	1.324042000
O	-0.307335000	-1.571055000	2.070366000
C	-0.906304000	-2.013060000	3.272795000
H	-1.647667000	-2.800863000	3.086303000
H	-1.403112000	-1.137988000	3.693126000
H	-0.153374000	-2.381618000	3.981030000
C	2.585198000	0.949506000	3.187754000
C	3.604849000	0.341021000	4.113810000
H	3.316865000	-0.673235000	4.415507000
H	4.581737000	0.265379000	3.621351000
H	3.728126000	0.939528000	5.021984000
H	2.284145000	2.730387000	4.360618000
H	2.651400000	-0.687668000	1.800388000
H	0.614921000	3.711247000	2.786194000
C	4.026959000	0.099155000	-0.902814000
C	4.309315000	1.298066000	-0.217697000
C	4.854882000	-1.012897000	-0.673157000
C	5.340702000	1.360333000	0.727499000

C	5.875832000	-0.966297000	0.284415000
C	6.103159000	0.222892000	0.970917000
H	5.553179000	2.281799000	1.256830000
H	6.500191000	-1.830105000	0.479795000
H	6.906617000	0.267285000	1.702320000
O	4.610154000	-2.103064000	-1.451196000
O	3.537220000	2.347897000	-0.565405000
C	5.423961000	-3.244051000	-1.278681000
H	5.314996000	-3.670972000	-0.273192000
H	5.079131000	-3.971087000	-2.016593000
H	6.482002000	-3.017557000	-1.465116000
C	3.748185000	3.597716000	0.062922000
H	2.993074000	4.263658000	-0.356077000
H	3.606030000	3.528342000	1.147628000
H	4.750894000	3.989078000	-0.155714000
C	-3.866744000	0.739273000	-0.795784000
C	-2.276356000	-1.958092000	-0.878536000
C	-2.280967000	-1.339201000	-2.117112000
H	-3.199182000	-2.185538000	-0.354237000
H	-1.394811000	-2.489524000	-0.534145000
H	-3.209424000	-1.061998000	-2.608401000
H	-1.401490000	-1.384373000	-2.753493000
C	-5.004141000	-0.212048000	-0.440754000
H	-4.894172000	-1.171159000	-0.967889000
C	-5.071311000	-0.461798000	1.077297000
H	-5.448612000	0.447464000	1.563736000
H	-4.034185000	-0.569435000	1.422934000
C	-5.877876000	-1.677025000	1.542222000
H	-6.928893000	-1.565444000	1.258607000
H	-5.516606000	-2.576591000	1.020968000
C	-5.777278000	-1.890085000	3.051432000
H	-6.160471000	-1.020784000	3.599403000
H	-6.354601000	-2.765177000	3.370772000
H	-4.736839000	-2.043059000	3.364963000
C	-6.313034000	0.347351000	-0.990571000
F	-6.639516000	1.527906000	-0.434036000
F	-7.356835000	-0.488664000	-0.785853000
F	-6.241469000	0.537915000	-2.326624000
H	-3.903637000	1.055797000	-1.843728000
H	-3.876915000	1.622900000	-0.153413000

88

A3,E21(Coor6)

Pd	2.012188000	-0.317431000	-0.393350000
P	-0.454100000	0.166844000	-0.294859000
C	-1.195990000	-0.269495000	1.333829000

C	-0.741941000	-1.389334000	2.049462000
S	0.532687000	-2.456502000	1.382688000
O	1.734181000	-1.490289000	1.333482000
O	0.103266000	-2.840484000	0.028713000
O	0.768676000	-3.514475000	2.365302000
C	-1.225638000	-1.656870000	3.325746000
C	-2.163193000	-0.806911000	3.908121000
C	-2.132827000	0.569918000	1.941443000
C	-1.378544000	-0.584113000	-1.706018000
C	-0.533391000	-1.049702000	-2.726149000
H	0.536662000	-1.074019000	-2.545637000
C	-1.027288000	-1.522509000	-3.937043000
H	-0.340578000	-1.881352000	-4.698871000
C	-2.401310000	-1.544061000	-4.148459000
H	-2.811840000	-1.903673000	-5.088583000
C	-3.254738000	-1.137871000	-3.128417000
H	-4.330396000	-1.201224000	-3.268118000
C	-2.778085000	-0.672864000	-1.894079000
C	-0.771647000	1.972190000	-0.423362000
C	-1.500227000	2.556076000	-1.460668000
H	-2.006883000	1.921355000	-2.178881000
C	-1.604791000	3.944255000	-1.578044000
H	-2.176542000	4.375873000	-2.394499000
C	-0.974900000	4.763075000	-0.648422000
H	-1.051087000	5.844596000	-0.728205000
C	-0.229789000	4.208589000	0.393765000
H	0.264309000	4.858603000	1.108014000
C	-0.116940000	2.821037000	0.500427000
O	0.610386000	2.190451000	1.452732000
C	1.250114000	2.955776000	2.455660000
H	2.003909000	3.628916000	2.027807000
H	1.738301000	2.232793000	3.109821000
H	0.523295000	3.540615000	3.033550000
C	-2.629302000	0.318946000	3.225958000
C	-3.652277000	1.238543000	3.838737000
H	-3.366210000	2.290421000	3.723185000
H	-4.627442000	1.113294000	3.352640000
H	-3.780892000	1.038595000	4.907327000
H	-2.529101000	-1.018308000	4.910776000
H	-2.491919000	1.443417000	1.404277000
H	-0.841397000	-2.524220000	3.853580000
C	-3.820774000	-0.426413000	-0.854526000
C	-4.071580000	-1.436199000	0.096714000
C	-4.701483000	0.665797000	-0.919883000
C	-5.133604000	-1.323245000	1.002003000

C	-5.755633000	0.796444000	-0.007042000
C	-5.955585000	-0.203225000	0.940986000
H	-5.321745000	-2.097584000	1.736542000
H	-6.425271000	1.647785000	-0.041866000
H	-6.783565000	-0.112334000	1.640049000
O	-4.470329000	1.553184000	-1.927449000
O	-3.237471000	-2.493695000	0.035900000
C	-5.344260000	2.654353000	-2.060116000
H	-5.303949000	3.312309000	-1.182102000
H	-4.999884000	3.205511000	-2.937348000
H	-6.380360000	2.329918000	-2.223385000
C	-3.424640000	-3.575589000	0.928076000
H	-2.620316000	-4.276534000	0.703595000
H	-3.339527000	-3.252539000	1.972425000
H	-4.398113000	-4.058586000	0.768721000
C	3.999946000	-0.954433000	0.019222000
C	1.958543000	1.313133000	-1.881737000
C	2.954742000	0.440600000	-2.261370000
H	2.187127000	2.216643000	-1.322114000
H	0.989595000	1.297355000	-2.369383000
H	3.989952000	0.663718000	-2.050788000
H	2.777212000	-0.327361000	-3.009402000
C	4.912544000	-1.521273000	-1.034790000
F	5.643151000	-0.605357000	-1.729209000
F	4.253386000	-2.261287000	-1.950443000
F	5.828318000	-2.342425000	-0.463278000
C	4.672485000	-0.003506000	1.005046000
H	4.009738000	0.066538000	1.876462000
H	5.591003000	-0.493106000	1.370675000
C	5.029180000	1.408243000	0.531192000
H	4.116802000	1.919273000	0.190523000
H	5.703892000	1.359660000	-0.331890000
C	5.687619000	2.242919000	1.631470000
H	5.016988000	2.291539000	2.501678000
H	6.595181000	1.729773000	1.979500000
C	6.045361000	3.657276000	1.179697000
H	5.154648000	4.208013000	0.850870000
H	6.511873000	4.232107000	1.988050000
H	6.747900000	3.637869000	0.337573000
H	3.662176000	-1.835705000	0.569746000

88

A3,E12(TS3)

Pd	-1.670415000	-0.546419000	-0.822950000
P	0.596488000	-0.465621000	-0.213500000
C	1.003936000	1.155409000	0.553974000

C	0.438389000	2.344895000	0.060788000
S	-0.594446000	2.387369000	-1.415266000
O	-1.833630000	1.593737000	-0.982859000
O	0.160858000	1.682152000	-2.463206000
O	-0.934214000	3.793831000	-1.645117000
C	0.657566000	3.547455000	0.725651000
C	1.431273000	3.580545000	1.884004000
C	1.779821000	1.213912000	1.713737000
C	1.741499000	-0.899302000	-1.593391000
C	1.094140000	-1.454136000	-2.708407000
H	0.008515000	-1.458016000	-2.730177000
C	1.801260000	-1.958600000	-3.794042000
H	1.265451000	-2.373947000	-4.643329000
C	3.190921000	-1.909086000	-3.784102000
H	3.765127000	-2.300704000	-4.619987000
C	3.845487000	-1.320947000	-2.707381000
H	4.928854000	-1.238377000	-2.713843000
C	3.151945000	-0.794593000	-1.608099000
C	0.932995000	-1.715424000	1.089264000
C	1.767369000	-2.812315000	0.865795000
H	2.333165000	-2.868178000	-0.056597000
C	1.896864000	-3.830881000	1.812032000
H	2.550626000	-4.674752000	1.611600000
C	1.184043000	-3.757112000	3.002377000
H	1.277841000	-4.541307000	3.749495000
C	0.330297000	-2.681212000	3.249081000
H	-0.228513000	-2.641889000	4.177904000
C	0.191198000	-1.671313000	2.294134000
O	-0.645231000	-0.615097000	2.433832000
C	-1.398784000	-0.488068000	3.623755000
H	-2.081513000	-1.336745000	3.759723000
H	-1.977393000	0.429327000	3.510432000
H	-0.746365000	-0.401199000	4.501995000
C	2.008210000	2.415646000	2.393235000
C	2.871596000	2.441491000	3.626683000
H	2.632509000	1.612344000	4.302416000
H	3.931307000	2.345063000	3.359711000
H	2.749102000	3.377120000	4.181796000
H	1.584772000	4.526815000	2.399001000
H	2.220808000	0.300069000	2.102793000
H	0.203174000	4.447678000	0.323569000
C	3.988704000	-0.078223000	-0.599572000
C	4.104478000	1.323329000	-0.694367000
C	4.825009000	-0.760525000	0.299402000
C	4.983782000	2.029956000	0.135460000

C	5.693348000	-0.061192000	1.147155000
C	5.759447000	1.326050000	1.050293000
H	5.068400000	3.108040000	0.064096000
H	6.325392000	-0.582999000	1.856282000
H	6.446038000	1.869543000	1.695125000
O	4.744269000	-2.119611000	0.266981000
O	3.334264000	1.891684000	-1.642545000
C	5.572565000	-2.858481000	1.139229000
H	5.339413000	-2.650789000	2.191680000
H	5.367633000	-3.909721000	0.927707000
H	6.635796000	-2.655508000	0.955051000
C	3.384825000	3.292653000	-1.830880000
H	2.649700000	3.504851000	-2.607484000
H	3.106854000	3.829303000	-0.916035000
H	4.382495000	3.613878000	-2.159599000
C	-3.895773000	-0.461166000	-1.323034000
C	-1.706433000	-2.604088000	-0.672481000
C	-2.984542000	-2.401104000	-1.268670000
H	-1.659082000	-2.891103000	0.377265000
H	-0.907407000	-3.019796000	-1.283603000
H	-3.870264000	-2.660649000	-0.701084000
H	-3.074945000	-2.582244000	-2.336948000
C	-4.689174000	-0.238992000	-0.023508000
H	-4.208797000	-0.768394000	0.811365000
C	-4.762099000	1.261156000	0.332863000
H	-5.379726000	1.769767000	-0.419195000
H	-3.749372000	1.657666000	0.205674000
C	-5.261763000	1.611710000	1.737516000
H	-6.310885000	1.324007000	1.861665000
H	-4.695705000	1.029201000	2.479854000
C	-5.103568000	3.102476000	2.033252000
H	-5.679074000	3.708750000	1.323342000
H	-5.456288000	3.348163000	3.041694000
H	-4.055377000	3.414498000	1.954653000
C	-6.073607000	-0.861997000	-0.139394000
F	-6.839808000	-0.233249000	-1.050027000
F	-6.743194000	-0.848639000	1.030464000
F	-6.016796000	-2.166003000	-0.519719000
H	-4.475701000	-0.970605000	-2.090081000
H	-3.557322000	0.482405000	-1.762724000

88

A3,E21(TS3)

Pd	-1.800509000	0.295895000	-0.448986000
P	0.496032000	-0.144205000	-0.387686000
C	1.157283000	0.094670000	1.312348000

C	0.685598000	1.134833000	2.133242000
S	-0.492007000	2.369127000	1.553968000
O	-1.754941000	1.535158000	1.307791000
O	0.056884000	2.899403000	0.295224000
O	-0.693038000	3.305198000	2.662639000
C	1.117815000	1.229824000	3.452446000
C	2.011145000	0.294249000	3.970432000
C	2.052979000	-0.830388000	1.853225000
C	1.447707000	0.792436000	-1.658544000
C	0.646560000	1.353676000	-2.665218000
H	-0.432802000	1.321063000	-2.551920000
C	1.196526000	1.980469000	-3.777969000
H	0.544775000	2.410606000	-4.533629000
C	2.579348000	2.062101000	-3.900031000
H	3.031516000	2.544156000	-4.763105000
C	3.385626000	1.551774000	-2.888258000
H	4.465329000	1.653923000	-2.954790000
C	2.852629000	0.927736000	-1.751505000
C	0.810157000	-1.910753000	-0.776560000
C	1.510344000	-2.311533000	-1.916027000
H	1.987703000	-1.561253000	-2.535678000
C	1.617052000	-3.659815000	-2.263061000
H	2.165122000	-3.946468000	-3.155938000
C	1.017374000	-4.624907000	-1.462829000
H	1.095269000	-5.678236000	-1.720414000
C	0.299455000	-4.254513000	-0.324799000
H	-0.172428000	-5.017544000	0.284847000
C	0.182614000	-2.904203000	0.012283000
O	-0.527348000	-2.450138000	1.072353000
C	-1.135433000	-3.382642000	1.944248000
H	-1.894451000	-3.982185000	1.425531000
H	-1.612189000	-2.789027000	2.724947000
H	-0.390327000	-4.049557000	2.396480000
C	2.492122000	-0.750326000	3.179482000
C	3.451285000	-1.775420000	3.725262000
H	2.988669000	-2.769669000	3.770936000
H	4.340147000	-1.858750000	3.090034000
H	3.779735000	-1.516194000	4.736747000
H	2.334486000	0.378037000	5.006026000
H	2.424919000	-1.636475000	1.226526000
H	0.733013000	2.041849000	4.061657000
C	3.838924000	0.562531000	-0.691356000
C	4.049704000	1.470283000	0.366734000
C	4.696691000	-0.542547000	-0.808472000
C	5.050378000	1.243551000	1.319143000

C	5.690528000	-0.786180000	0.148552000
C	5.851225000	0.113387000	1.199230000
H	5.206661000	1.938016000	2.136325000
H	6.344617000	-1.646665000	0.069547000
H	6.631412000	-0.065967000	1.935129000
O	4.504426000	-1.327385000	-1.904929000
O	3.239515000	2.546412000	0.359215000
C	5.342931000	-2.449688000	-2.077795000
H	5.226573000	-3.172547000	-1.259650000
H	5.029783000	-2.915898000	-3.013877000
H	6.398391000	-2.157338000	-2.156784000
C	3.352542000	3.507475000	1.390991000
H	2.559829000	4.230392000	1.198830000
H	3.193934000	3.053762000	2.376530000
H	4.331712000	4.004677000	1.365810000
C	-4.031189000	0.845883000	-0.291290000
C	-2.085884000	-0.991963000	-2.026000000
C	-3.400768000	-0.443790000	-1.960895000
H	-1.925190000	-2.009646000	-1.672619000
H	-1.453903000	-0.707731000	-2.865734000
H	-4.215181000	-1.085681000	-1.650144000
H	-3.663020000	0.295836000	-2.707677000
C	-5.141703000	1.427827000	-1.139294000
F	-5.978841000	0.519816000	-1.693824000
F	-4.670333000	2.194038000	-2.146697000
F	-5.916636000	2.230995000	-0.376934000
C	-4.524277000	0.059676000	0.923252000
H	-3.704359000	0.038224000	1.649101000
H	-5.313360000	0.668100000	1.392218000
C	-5.064080000	-1.358495000	0.721902000
H	-4.260832000	-2.012054000	0.351467000
H	-5.855929000	-1.364125000	-0.038299000
C	-5.618811000	-1.946550000	2.021277000
H	-4.834619000	-1.926711000	2.791558000
H	-6.424180000	-1.298840000	2.394716000
C	-6.144790000	-3.370806000	1.858446000
H	-5.354328000	-4.051616000	1.517821000
H	-6.536942000	-3.763600000	2.803457000
H	-6.954645000	-3.412107000	1.119815000
H	-3.509169000	1.743140000	0.062594000

88

A3,E12(P3)

Pd	-1.524822000	0.210659000	-0.899980000
P	0.570107000	-0.316873000	-0.267127000
C	1.205547000	1.070871000	0.758432000

C	0.884845000	2.413121000	0.482219000
S	-0.055037000	2.940385000	-0.971637000
O	-1.438777000	2.344188000	-0.717275000
O	0.609227000	2.307384000	-2.124468000
O	-0.086799000	4.404624000	-0.927545000
C	1.298112000	3.412370000	1.358453000
C	2.018823000	3.095534000	2.508075000
C	1.935395000	0.776516000	1.913608000
C	1.653892000	-0.735116000	-1.695305000
C	0.958387000	-1.052811000	-2.872106000
H	-0.118632000	-0.917907000	-2.891112000
C	1.615045000	-1.507683000	-4.009803000
H	1.046115000	-1.738264000	-4.906326000
C	2.998327000	-1.648936000	-3.987966000
H	3.532111000	-2.004380000	-4.865697000
C	3.703386000	-1.303143000	-2.840346000
H	4.787448000	-1.374700000	-2.829847000
C	3.064789000	-0.832414000	-1.684378000
C	0.593870000	-1.792052000	0.812763000
C	1.235517000	-2.972643000	0.430225000
H	1.827331000	-2.984133000	-0.477488000
C	1.139243000	-4.129977000	1.203184000
H	1.645667000	-5.036534000	0.885175000
C	0.389558000	-4.112741000	2.373872000
H	0.307189000	-5.007340000	2.986025000
C	-0.276132000	-2.953729000	2.772935000
H	-0.867312000	-2.960408000	3.682146000
C	-0.189390000	-1.798790000	1.992048000
O	-0.828564000	-0.642844000	2.281848000
C	-1.617595000	-0.565556000	3.454342000
H	-2.444063000	-1.286988000	3.429434000
H	-2.018819000	0.448306000	3.471791000
H	-1.012298000	-0.733815000	4.354015000
C	2.353725000	1.772997000	2.801758000
C	3.157080000	1.417116000	4.024509000
H	2.749013000	0.535057000	4.531089000
H	4.193657000	1.183255000	3.752252000
H	3.177006000	2.242016000	4.743944000
H	2.320616000	3.890810000	3.186733000
H	2.188642000	-0.257589000	2.130673000
H	1.032070000	4.438615000	1.124898000
C	3.967898000	-0.393516000	-0.579125000
C	4.303190000	0.972352000	-0.483692000
C	4.651370000	-1.312220000	0.233703000
C	5.254930000	1.414600000	0.443531000

C	5.592216000	-0.877397000	1.175763000
C	5.880136000	0.482075000	1.264117000
H	5.509435000	2.465321000	0.518556000
H	6.110244000	-1.581131000	1.816822000
H	6.623085000	0.819881000	1.982803000
O	4.349521000	-2.623934000	0.026324000
O	3.659038000	1.773985000	-1.352558000
C	5.026605000	-3.593521000	0.797766000
H	4.809380000	-3.483520000	1.868264000
H	4.656353000	-4.561435000	0.454641000
H	6.112375000	-3.547288000	0.641132000
C	3.914986000	3.165681000	-1.337803000
H	3.234368000	3.589965000	-2.075708000
H	3.695739000	3.600252000	-0.355362000
H	4.956165000	3.380571000	-1.613917000
C	-4.224444000	-0.433254000	-1.639058000
C	-2.070666000	-1.734282000	-1.064971000
C	-3.318185000	-1.649588000	-1.941249000
H	-2.286830000	-2.072273000	-0.043299000
H	-1.319370000	-2.405767000	-1.490605000
H	-3.900910000	-2.575303000	-1.854319000
H	-3.018798000	-1.572571000	-2.994808000
C	-4.795866000	-0.289302000	-0.210625000
H	-4.038737000	-0.603216000	0.520376000
C	-5.160272000	1.184589000	0.062827000
H	-5.906440000	1.505590000	-0.676860000
H	-4.255630000	1.772114000	-0.143756000
C	-5.645966000	1.544534000	1.469810000
H	-6.619754000	1.085415000	1.669873000
H	-4.951387000	1.127243000	2.212543000
C	-5.746856000	3.057369000	1.658614000
H	-6.448562000	3.499737000	0.940880000
H	-6.101079000	3.308005000	2.665012000
H	-4.774820000	3.543142000	1.512324000
C	-5.976927000	-1.221862000	0.000048000
F	-7.053982000	-0.841433000	-0.717602000
F	-6.357572000	-1.276733000	1.294135000
F	-5.697820000	-2.495077000	-0.360117000
H	-5.054884000	-0.419290000	-2.357295000
H	-3.677159000	0.501959000	-1.868243000

88

A3,E21(P3)

Pd	1.642355000	0.120265000	-0.451483000
P	-0.493888000	-0.186267000	0.211338000
C	-1.544277000	-0.248584000	-1.295633000

C	-1.296061000	0.567765000	-2.414532000
S	0.023673000	1.801641000	-2.470664000
O	1.279446000	0.933601000	-2.394784000
O	-0.139738000	2.628324000	-1.261091000
O	-0.092798000	2.469739000	-3.769172000
C	-2.066615000	0.415188000	-3.562891000
C	-3.072294000	-0.547604000	-3.619530000
C	-2.557532000	-1.208057000	-1.374922000
C	-1.070511000	1.044661000	1.451974000
C	-0.034624000	1.669384000	2.162578000
H	0.989496000	1.483081000	1.857173000
C	-0.289267000	2.531627000	3.222817000
H	0.537476000	3.003386000	3.746785000
C	-1.605650000	2.789885000	3.589613000
H	-1.828604000	3.460462000	4.415577000
C	-2.642771000	2.204753000	2.871529000
H	-3.673673000	2.431911000	3.128762000
C	-2.410654000	1.339124000	1.793249000
C	-0.697708000	-1.813772000	1.022434000
C	-1.122766000	-1.931492000	2.348483000
H	-1.446212000	-1.045243000	2.882298000
C	-1.149014000	-3.169829000	2.990737000
H	-1.480038000	-3.237188000	4.022971000
C	-0.745365000	-4.308564000	2.302408000
H	-0.761604000	-5.279455000	2.791272000
C	-0.307617000	-4.220954000	0.980939000
H	0.010871000	-5.117154000	0.459699000
C	-0.271725000	-2.979402000	0.341674000
O	0.160613000	-2.796419000	-0.925889000
C	0.605997000	-3.912040000	-1.673579000
H	1.468151000	-4.395073000	-1.196507000
H	0.901976000	-3.514980000	-2.645159000
H	-0.197327000	-4.647424000	-1.808511000
C	-3.332629000	-1.376051000	-2.527603000
C	-4.410228000	-2.427124000	-2.572895000
H	-3.999102000	-3.428731000	-2.395872000
H	-5.167517000	-2.245190000	-1.801651000
H	-4.913435000	-2.440215000	-3.544823000
H	-3.656176000	-0.658909000	-4.530948000
H	-2.752727000	-1.845258000	-0.516970000
H	-1.848551000	1.054018000	-4.413134000
C	-3.626045000	0.886403000	1.054053000
C	-4.035145000	1.617057000	-0.079764000
C	-4.474285000	-0.113833000	1.553459000
C	-5.236714000	1.314840000	-0.731887000

C	-5.673912000	-0.429193000	0.902506000
C	-6.035392000	0.292298000	-0.232592000
H	-5.547615000	1.871185000	-1.608465000
H	-6.328509000	-1.206590000	1.278972000
H	-6.972148000	0.057747000	-0.732439000
O	-4.056027000	-0.725924000	2.696603000
O	-3.200334000	2.608179000	-0.445319000
C	-4.887660000	-1.715361000	3.264922000
H	-5.019112000	-2.572047000	2.590870000
H	-4.379599000	-2.048981000	4.171621000
H	-5.872666000	-1.309926000	3.531492000
C	-3.498512000	3.380335000	-1.593184000
H	-2.646604000	4.047876000	-1.719679000
H	-3.593087000	2.748850000	-2.484436000
H	-4.419933000	3.961372000	-1.452140000
C	4.318479000	0.942166000	0.669216000
C	2.391006000	-0.699134000	1.251200000
C	3.555795000	0.156534000	1.757520000
H	2.728520000	-1.674125000	0.867796000
H	1.680601000	-0.894513000	2.057987000
H	4.263944000	-0.490397000	2.295951000
H	3.192333000	0.882478000	2.493513000
C	5.530426000	1.632121000	1.273943000
F	6.391112000	0.769874000	1.860163000
F	5.157859000	2.513553000	2.225437000
F	6.229728000	2.317678000	0.348385000
C	4.673850000	0.133431000	-0.592770000
H	3.730297000	-0.175749000	-1.094575000
H	5.150404000	0.805685000	-1.315662000
C	5.527912000	-1.121345000	-0.406921000
H	5.060945000	-1.790106000	0.329210000
H	6.502043000	-0.840813000	0.011476000
C	5.736468000	-1.880477000	-1.718319000
H	4.757952000	-2.157771000	-2.136677000
H	6.200450000	-1.210092000	-2.455197000
C	6.596482000	-3.131655000	-1.553860000
H	6.142503000	-3.836575000	-0.846055000
H	6.726364000	-3.654830000	-2.507954000
H	7.594145000	-2.880072000	-1.174144000
H	3.680740000	1.776398000	0.341479000
